# Hydrocephalus is an independent factor affecting morbidity and mortality of ICH patients: Systematic review and meta-analysis

**DOI:** 10.1016/j.wnsx.2023.100194

**Published:** 2023-04-10

**Authors:** Petra Octavian Perdana Wahjoepramono, Aloysius Bagus Sasongko, Danny Halim, Jenifer Kiem Aviani, Patrick Putra Lukito, Achmad Adam, Yeo Tseng Tsai, Eka Julianta Wahjoepramono, Julius July, Tri Hanggono Achmad

**Affiliations:** a Department of Neurosurgery, Faculty of Medicine, Pelita Harapan University/Siloam Hospitals, Tangerang, Banten, Indonesia; b Post Graduate Program, Faculty of Medicine, Universitas Padjadjaran, Bandung, West Java, Indonesia; c Department of Neurosurgery, Faculty of Medicine, Universitas Padjadjaran/Dr. Hasan Sadikin General Hospital, Bandung, West Java, Indonesia; d Research Center for Medical Genetics, Faculty of Medicine, Universitas Padjadjaran, Bandung, West Java, Indonesia; e Division of Neurosurgery, Department of Surgery, National University Hospital, Singapore; f Department of Basic Medical Science, Faculty of Medicine, Universitas Padjadjaran, Bandung, West Java, Indonesia

**Keywords:** Stroke, Intracerebral hemorrhage, Intraventricular extension, Hydrocephalus, Mortality, Good functional outcome

## Abstract

**Background:**

Despite advances in our knowledge of the causes, preventions, and treatments of stroke, it continues to be a leading cause of death and disability. The most common type of stroke-related morbidity and mortality is intracerebral haemorrhage (ICH). Many prognostication scores include an intraventricular extension (IVH) after ICH because it affects mortality independently. Although it is a direct result of IVH and results in significant damage, hydrocephalus (HC) has never been taken into account when calculating prognostication scores. This study aimed to evaluate the significance of hydrocephalus on the outcomes of ICH patients by meta-analysis.

**Methods:**

Studies that compared the rates of mortality and/or morbidity in patients with ICH, ICH with IVH (ICH ​+ ​IVH), and ICH with IVH and HC (ICH ​+ ​IVH ​+ ​HC) were identified. A meta-analysis was performed by using Mantel-Haezel Risk Ratio at 95% significance.

**Results:**

This meta-analysis included thirteen studies. The findings indicate that ICH ​+ ​IVH ​+ ​HC has higher long-term (90-day) and short-term (30-day) mortality risks than ICH (4.26 and 2.30 higher risks, respectively) and ICH ​+ ​IVH (1.96 and 1.54 higher risks). Patients with ICH ​+ ​IVH ​+ ​HC have lower rates of short-term (3 months) and long-term (6 months) good functional outcomes than those with ICH (0.66 and 0.38 times) or ICH ​+ ​IVH (0.76 and 0.54 times). Confounding variables included vascular comorbidities, haemorrhage volume, midline shift, and an initial GCS score below 8.

**Conclusion:**

Hydrocephalus causes a poorer prognosis in ICH patients. Thus, it is reasonable to suggest the inclusion of hydrocephalus in ICH prognostication scoring systems.

## Abbreviations

CI: Confidence Interval
EVD: External Ventricular Drainage
GCS: Glasgow Coma Scale
GOS: Glasgow Outcome Scale
HC: Hydrocephalus
I^2^: Inconsistency Index
ICH: Intracerebral Hemorrhage
ICH ​+ ​IVH: Intracerebral Hemorrhage with IntraventricularHemorrhage
ICH ​+ ​IVH ​+ ​HC: Intracerebral Hemorrhage with IntraventricularHemorrhage and Hydrocephalus
IVH: Intraventricular Hemorrhage
mRS: Modified Rankin Scale
PRISMA: Preferred Reporting Items for SystematicReviews and Meta-Analyses
RR: Risk Ratio

## 1 Introduction

Spontaneous intracerebral haemorrhage (ICH) is defined as non-traumatic intracerebral bleeding. Certain conditions, such as hypertension, amyloid angiopathy, and anticoagulant consumption, have been acknowledged as predisposing factors of ICH. Although ICH constituted less than 30% of all stroke cases, its mortality rate is higher than ischemic stroke, reaching up to 50% within 30 days. Furthermore, it has been estimated that only 12–39% of the surviving patients could acquire long-term functional independence.^1^, ^2^, ^3^, ^4^, ^5^ Persistent high mortality and morbidity rates in ICH patients suggest that our current understanding of this fatal and debilitating condition remains far from ideal.

Further understanding of the modifiable factors that lead to the incidence of ICH is essential to decreasing its incidence. Ten modifiable factors that increased the risk of ICH have been identified by the findings of large-scale studies like INTERSTROKE.^6^ Interestingly, despite acknowledging these modifiable factors, the prevalence of ICH remained high or even increased steadily, particularly in low-to-middle-income countries.^2^^,^^5^

On its curative aspect, knowing the possible complications of ICH and their prompt management might be keys to improving ICH patient outcomes. A previous study suggested that the site of bleeding in 65% of spontaneous ICH cases are the basal ganglia and thalamus, of which their anatomical proximity to the cerebral ventricles carries a high risk of intraventricular extension of the haemorrhage (IVH).^7^ Thus, up to 42–52% of ICH patients developed IVH.^8^, ^9^, ^10^ The presence of blood in the ventricles might lead to hydrocephalus (HC) development.^11^^,^^12^ Previous studies indicated that the development of IVH and HC could worsen the prognosis of ICH patients.^13^, ^14^, ^15^ Surprisingly, despite being identified as a complication affecting ICH patients' prognosis, HC has never been included in any ICH prognostic scoring systems.^16^, ^17^, ^18^ This study aimed to evaluate the significance of HC in ICH patients' prognosis.

## 2 Methods

### 2.1 Literature search and identification

This meta-analysis was conducted according to the Preferred Reporting Items for Systematic Reviews and Meta-analyses (PRISMA) reporting guidelines.^19^ PubMed/MEDLINE, Embase, Ovid, Scopus, Web of Science, and Google Scholars were utilized to collect publications up to November 21, 2021. The following search terms were applied (“spontaneous intracerebral hemorrhage” AND “intraventricular extension” AND “hydrocephalus”) AND (“mortality” OR “morbidity” OR “functional outcome”). Additional studies were identified by screening the references.

### 2.2 Inclusion and exclusion criteria

Studies were included if they: 1) reported spontaneous intracerebral hemorrhage cases (ICH) in patients aged ≥18-year-old, 2) compared outcomes in patients with ICH only to ICH patients who developed intraventricular extension with/without hydrocephalus (ICH ​+ ​IVH or ICH ​+ ​IVH ​+ ​HC), 3) reported mortality and/or functional recovery.

Studies were excluded if they: 1) were not based on original data, such as reviews, systematic reviews, comments, or editorial letters; 2) did not have a control group (e.g. case reports); 3) were not written in English, 4) were unpublished data, 5) reported ICH secondary to trauma or other conditions (aneurysms, arteriovenous malformations, or tumors), 6) reported subarachnoid hemorrhage, 7) reported primary IVH, 8) did not define the end-point outcomes, 9) did not report comparisons between ICH, ICH-IVH, and ICH-IVH-HC, 10) did not report the number of patients, 11) did not report outcomes of interest.

### 2.3 Data collection and analysis

Four authors (PW, ABS, DH, JKA) independently reviewed the abstract from every article. If the abstract met the inclusion criteria, its full-text article was reviewed. Reference lists on the identified publications were screened for relevant yet previously unidentified studies. Author, country, year of publication, number of patients, inclusion and exclusion criteria for patients, reported outcomes, patients' characteristics (age, sex, comorbidity), site of haemorrhage, haemorrhage volume, mRS, midline shift, treatment, and grouping (ICH, ICH ​+ ​IVH, ICH ​+ ​IVH ​+ ​HC) were all retrieved from each article.

### 2.4 Data synthesis

Short- and long-term mortality were analyzed. Short-term mortality is all-cause mortality during the 30 days after hospital admission. Long-term mortality is all-cause mortality during the 90 days after hospital admission. During the 3-month and 6-month follow-ups, functional outcome was evaluated using the modified Rankin Scale (mRS) or Glasgow Outcome Scale (GOS). Patients were classified to have good functional recovery (without symptoms, symptomatic but no disability, and slight disability) if their mRS was less than 3 or GOS more than 4.

### 2.5 Statistical analysis

Meta-analysis was performed using Mantel-Haezel Risk Ratio (RR) with a 95% Confidence Interval (CI) for dichotomous data in RevMan version 5.3 software (Cochrane Collaboration). The inconsistency index (I^2^) test evaluated heterogeneity across studies. Values above 50% or p-value< 0.05 indicate significant heterogeneity. Regression-based Egger's, Habord's, and Peter's tests were used to evaluate small study effects and the risk of publication bias. P-values less than 0.05 indicates a significant bias. The quality of the study was assessed using the risk of bias criteria as specified on the Newcastle–Ottawa Scale (NOS).^20^ The certainty of the evidence for outcomes was evaluated using the Guideline Development Tool from GRADEpro. Five domains assess the evidence: risk of bias, inconsistency, indirectness of evidence, imprecision, and publication bias. The large magnitude of an effect, the effect of plausible residual confounding, and the dose–response gradient are three additional domains that raise confidence in the evidence. Evidence's degree of certainty was rated as very low, low, moderate, and high based on the guidance from Kirmayr et al.^21^

## 3 Results

**Fig. 1:**
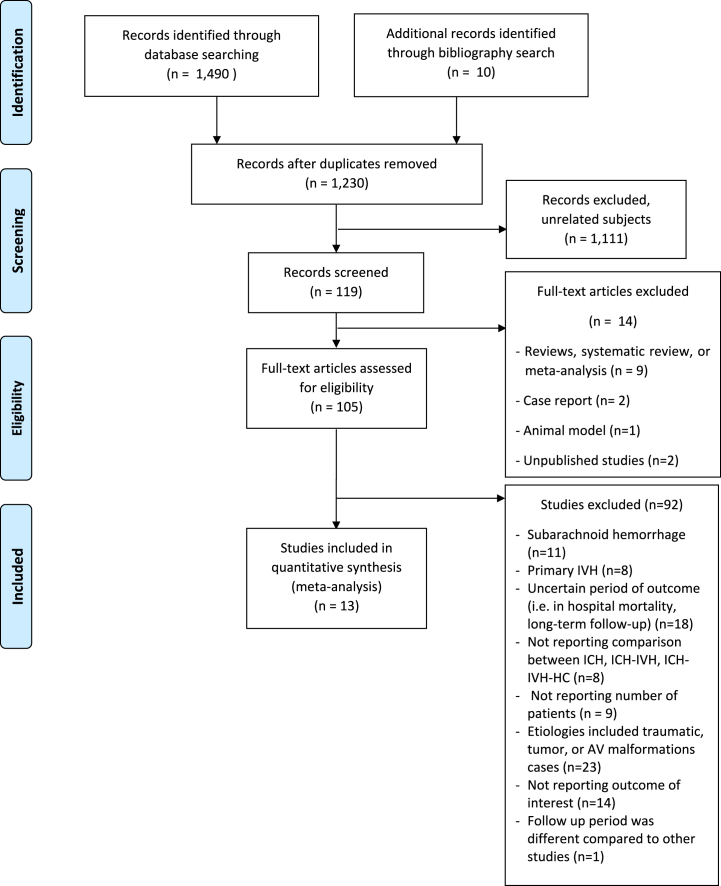
Flow diagram of study selection.

During the initial literature search, 1490 studies were found, and ten additional studies were identified during the reference screening (Fig. 1). Two hundred and seventy articles were eliminated as duplicates, and 1111 articles were excluded after reviewing the titles and abstracts because of their unrelated subject matter. Following a review of 125 records, 13 articles were included in the meta-analysis.^22^, ^23^, ^24^, ^25^, ^26^, ^27^, ^28^, ^29^, ^30^, ^31^, ^32^, ^33^, ^34^ In Supplemental Table I, characteristics of the 105 full-text articles that passed the eligibility screening are listed. Supplemental Table II shows the New Castle Ottawa scales of the included studies.

### 3.1 Intracerebral haemorrhage with intraventricular extension (ICH ​+ ​IVH) vs. intracerebral haemorrhage (ICH) only

#### 3.1.1 Mortality

**Fig. 2:**
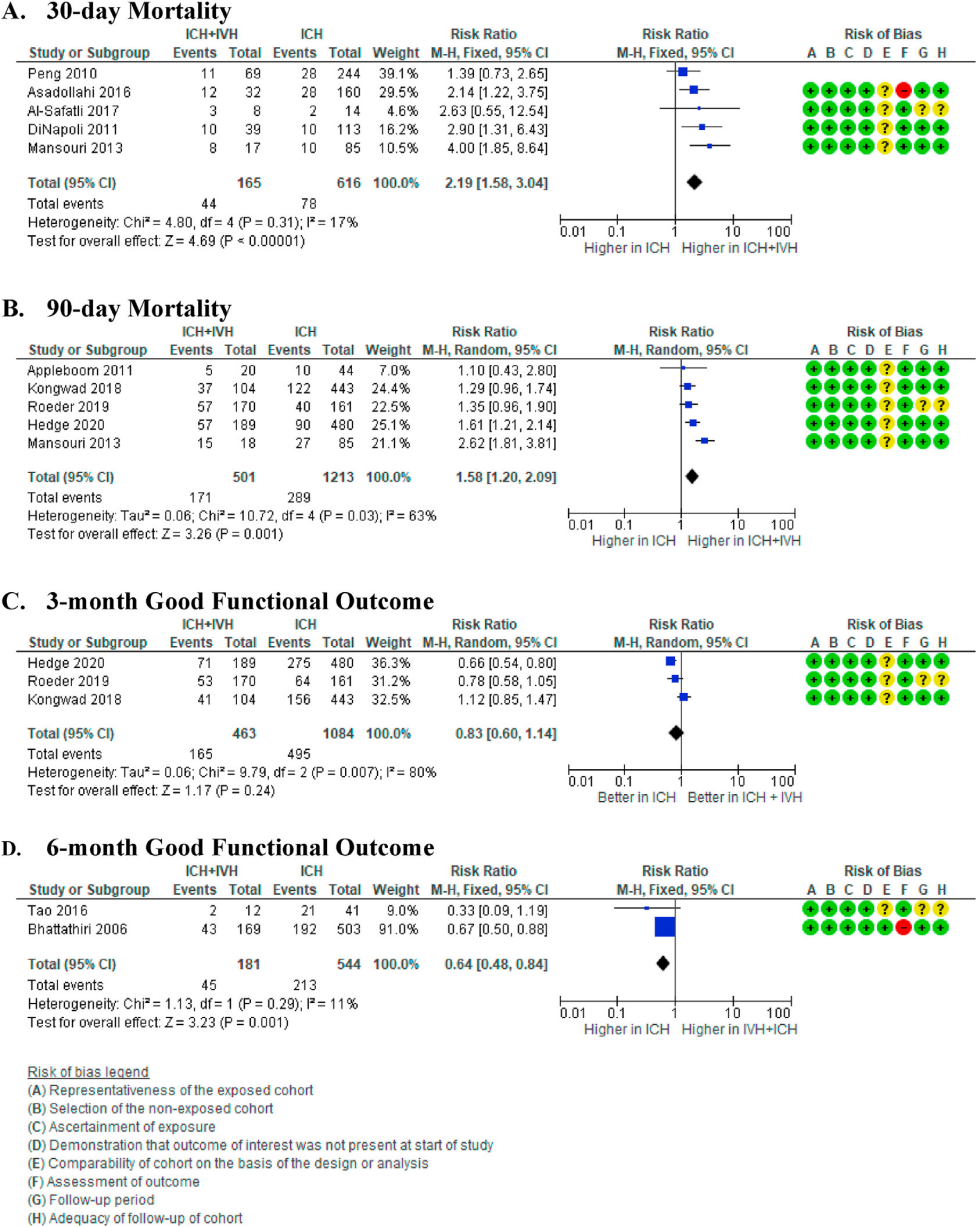
Forest plot from meta-analysis on mortality in patients with intraventricular extension. A) 30-day and B) 90-day mortality in patients with intracerebral haemorrhage and intraventricular extension (ICH ​+ ​IVH) compared to patients acquired only intracerebral haemorrhage (ICH).

Five studies compared the 30-day mortality rates in patients with ICH to those diagnosed with ICH and IVH.^22^, ^23^, ^24^, ^25^, ^26^ The rate was significantly higher in patients who were diagnosed with ICH ​+ ​IVH compared to those who only had ICH (RR 2.19 [95% CI 1.58, 3.04] (p ​< ​0.00001), I^2^ ​= ​17% (p ​= ​0.31)). The median mortality risk was 118 per 1000 in ICH cases and 258 per 1000 in ICH ​+ ​IVH cases, with moderate certainty of evidence (Fig. 2A).

A meta-analysis from 5 studies suggested that the 90-day mortality rate was also higher in the ICH ​+ ​IVH group than ICH-only group (RR 1.58 [95% CI 1.20, 2.09] (p ​= ​0.001), I^2^ = 63% (p = 0.003)).^26^, ^27^, ^28^, ^29^, ^30^ The median mortality risk was 248 per 1000 in ICH-only cases compared to 350 mortalities per 1000 in ICH ​+ ​IVH cases, with low certainty of evidence (Fig. 2B).

The meta-analysis on 30-day and 90-day mortality shows no small study effect and publication bias (p-Egger, p-Harbord, p-Peter >0.05) (Supplemental Table III). Supplemental Table IV presents the certainty of evidence.

#### 3.1.2 Good functional outcome

Three studies reported rates of good functional recovery at a 3-month follow-up.^28^, ^29^, ^30^ Pooled analysis from these studies suggested that after three months, there were fewer patients with good recovery in ICH ​+ ​IVH group compared to the ICH-only group. Nonetheless, these differences were not statistically significant (RR 0.83 [95% CI 0.60, 1.14] (p ​= ​0.24), I^2^ ​= ​80% (p ​= ​0.007). The median probability of good functional outcome was 398 per 1000 ICH cases and 310 per 1000 ICH ​+ ​IVH cases, with low certainty of evidence (Fig. 2C).

After six months, the rate of good functional recovery in ICH ​+ ​IVH patients declined further compared to patients with ICH-only (RR 0.62 [95% CI 0.47, 0.81] (p ​= ​0.0005), I^2^ = 51% (p = 0.15)).^33^^,^^34^ The median probability of good functional outcome was 447 per 1000 ICH-only cases and 286 per 1000 ICH ​+ ​IVH cases with low certainty of evidence (Fig. 2D).

Supplemental Table III shows no small study effect and publication bias in 3-month functional recovery (p-Egger, p-Harbord, p-Peter >0.05). Supplemental Table IV describes the certainty of evidence. Since only two studies were included in the meta-analysis on functional recovery at 6-month follow-up, publication bias and small study effect could not be calculated.

### 3.2 Intracerebral haemorrhage with intraventricular extension and hydrocephalus (ICH ​+ ​IVH ​+ ​HC) vs. intracerebral haemorrhage (ICH) only

#### 3.2.1 Mortality

**Fig. 3:**
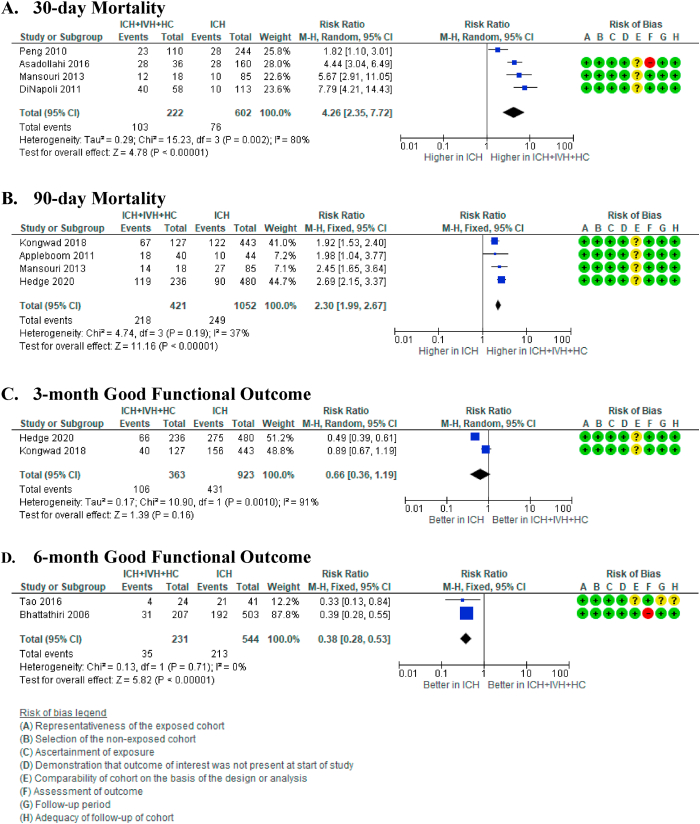
Forest plot from meta-analysis on mortality in patients with hydrocephalus. A) 30-day and B) 90-day mortality in patients with hydrocephalus after intraventricular extension of intracerebral haemorrhage (ICH ​+ ​IVH ​+ ​HC) compared to patients diagnosed with intracerebral haemorrhage (ICH). C) 30-day and D) 90-day mortality in patients with hydrocephalus after intraventricular extension of intracerebral haemorrhage (ICH ​+ ​IVH ​+ ​HC) compared to patients diagnosed with intracerebral haemorrhage with intraventricular extension (ICH ​+ ​IVH).

Four studies compared 30-day mortality between patients who developed hydrocephalus after the intraventricular extension of intracerebral hemorrhage (ICH ​+ ​IVH ​+ ​HC) and patients who were only diagnosed with intracerebral hemorrhage (ICH).^22^^,^^23^^,^^25^^,^^26^ The 30-day mortality rate was higher in patients with ICH ​+ ​IVH ​+ ​HC than in patients who were only diagnosed with ICH (RR 4.26 [95% CI 2.35, 7.72] (p ​< ​0.00001), I^2^ ​= ​80% (p ​= ​0.002)). The median mortality risk was 116 per 1000 ICH cases and 494 mortality per 1000 ICH ​+ ​IVH ​+ ​HC cases, with moderate certainty of evidence (Fig. 3A).

Based on the meta-analysis that included another four studies, the 90-day mortality rate was higher in the ICH ​+ ​IVH ​+ ​HC group compared to the ICH-only group (RR 2.30 [95% CI 1.99 2.67] (p ​< ​0.00001), I^2^ = 37% (p = 0.19)).^26^, ^27^, ^28^^,^^30^ The median mortality risk was 251 per 1000 ICH cases compared to 577 mortality per 1000 ICH ​+ ​IVH cases, with moderate certainty of evidence (Fig. 3B).

Meta-analysis on 30-day and 90-day mortality shows no small study effect and publication bias (p-Egger, p-Harbord, p-Peter >0.05) (seeSupplemental Table III). The certainty of the evidence is shown in Supplemental Table IV.

#### 3.2.2 Good functional outcome

Two studies reported rates of good functional outcomes at 3-month follow-up in ICH ​+ ​IVH ​+ ​HC patients compared to ICH-only patients. The result indicated fewer patients with good functional recovery in ICH ​+ ​IVH ​+ ​HC patients compared to ICH-only patients.28,30 However, this difference was not statistically significant (RR 0.66 [95% CI 0.36, 1.19] (p ​= ​0.16), I^2^ ​= ​91% (p ​= ​0.0010)). The median probability of a good functional outcome is 463 per 1000 ICH cases and 306 per 1000 ICH ​+ ​IVH ​+ ​HC cases, with very low certainty of evidence (Fig. 3C).

After 6-months, the rate of good functional recovery in ICH ​+ ​IVH ​+ ​HC patients declined further compared to ICH-only patients (RR 0.38 [95% CI 0.28, 0.53] (p ​< ​0.00001), I^2^ ​= ​0% (p ​= ​0.71)).33,34 The median probability of a good functional outcome is 447 per 1000 ICH cases and 170 per 1000 ICH ​+ ​IVH ​+ ​HC cases, with moderate certainty of evidence (Fig. 3D). Since only two studies could be included in the meta-analyses for 3- and 6-months of good functional recovery, publication bias and small study effect could not be calculated. The analysis of the certainty of the evidence is described in Supplemental Table IV.

### 3.3 Intracerebral haemorrhage with intraventricular extension and hydrocephalus (ICH ​+ ​IVH ​+ ​HC) vs. intracerebral haemorrhage with intraventricular extension (ICH ​+ ​IVH)

#### 3.3.1 Mortality

**Fig. 4:**
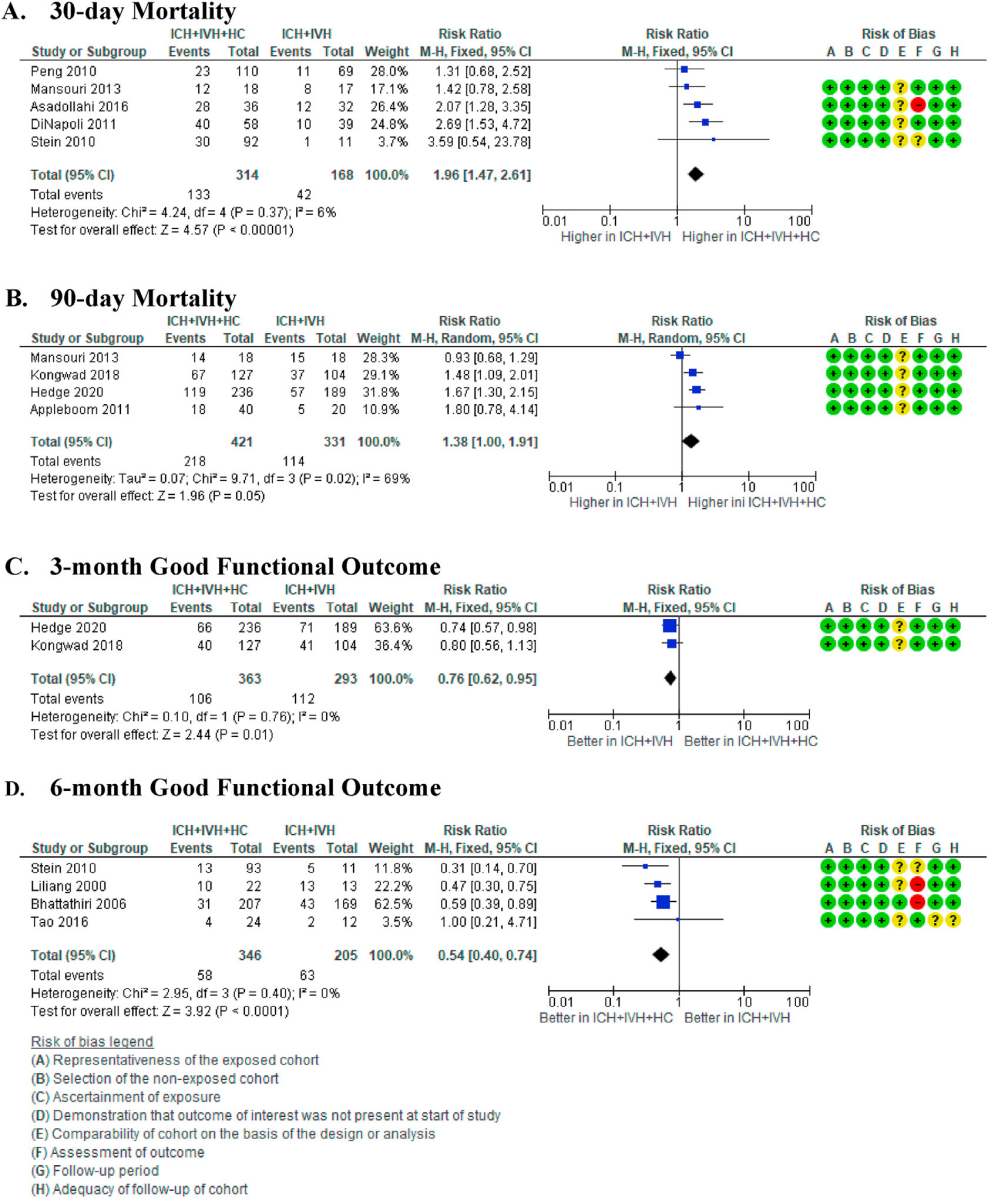
Forest plot from meta-analysis on functional recovery. A) 3-month and B) 6-month functional recovery in patients with intracerebral haemorrhage and intraventricular extension (ICH ​+ ​IVH) compared to intracerebral haemorrhage only (ICH). C) 6-month functional recovery in patients with hydrocephalus after intraventricular extension of intracerebral haemorrhage (ICH ​+ ​IVH ​+ ​HC) compared to intracerebral haemorrhage only (ICH). D) 6-month functional recovery in patients with hydrocephalus after intraventricular extension of intracerebral haemorrhage (ICH ​+ ​IVH ​+ ​HC) compared to intracerebral haemorrhage with extraventricular extension (ICH ​+ ​IVH).

Five studies compared the 30-day mortality rate in ICH ​+ ​IVH ​+ ​HC patients to the rate in ICH ​+ ​IVH patients.^22^^,^^23^^,^^25^^,^^26^^,^^31^ The result showed that the 30-day mortality rate in ICH ​+ ​IVH ​+ ​HC patients is significantly higher than in ICH ​+ ​IVH patients (RR 1.96 [95% CI 1.47, 2.61] (p ​< ​0.00001), I^2^ ​= ​6% (p ​= ​0.37)). The median mortality risk is 256 per 1000 ICH ​+ ​IVH cases and 502 mortality per 1000 ICH ​+ ​IVH ​+ ​HC cases, with moderate certainty of evidence (Fig. 4A).

Based on the meta-analysis of 4 studies, the 90-day mortality rate was also higher in ICH ​+ ​IVH ​+ ​HC patients compared to ICH ​+ ​IVH patients (RR 1.54 [95% CI 1.25, 1.90] (p ​< ​0.0001), I^2^ = 85% (p = 0.001)).^26^, ^27^, ^28^^,^^30^ The median mortality risk was 329 per 1000 ICH ​+ ​IVH cases compared to 454 per 1000 ICH ​+ ​IVH ​+ ​HC cases, with low certainty of evidence (Fig. 4B).

Meta-analysis on 30-day and 90-day mortality shows no small study effect and publication bias (p-Egger, p-Harbord, p-Peter >0.05) (see Supplemental Table III). The certainty of the evidence is shown in Supplemental Table IV.

#### 3.3.2 Good functional outcome

Based on the meta-analysis that included two studies, at 3-month follow-up, there are fewer patients with good functional recovery among ICH ​+ ​IVH ​+ ​HC patients compared to patients with ICH ​+ ​IVH (RR 0.76 [95% CI 0.62, 0.95] (p ​= ​0.01), I^2^ = 0% (p = 0.76)).^28^^,^^30^ The median probability of a good functional outcome is 385 per 1000 ICH ​+ ​IVH cases and 293 per 1000 ICH ​+ ​IVH ​+ ​HC cases with very low certainty of evidence (Fig. 4C).

At the 6-month follow-up, the rate of good functional recovery in ICH ​+ ​IVH ​+ ​HC patients declined further when compared to the same rate in ICH ​+ ​IVH patients (RR 0.54 [95% CI 0.40, 0.74] (p ​< ​0.0001), I^2^ = 0% (p = 0.40)).^17^^,^^21^^,^^25^^,^^26^ The median probability of a good functional outcome is 355 per 1000 ICH ​+ ​IVH cases and 192 per 1000 ICH ​+ ​IVH ​+ ​HC cases, with low certainty of evidence (Fig. 4D).^31^, ^32^, ^33^, ^34^

Meta-analysis on 6-month good functional recovery shows no small study effect and publication bias (p-Egger, p-Harbord, p-Peter >0.05) (see Supplemental Table III). Since there are only two studies in the meta-analysis on functional recovery at 3-month follow-up, publication bias and small study effect are unavailable. Supplemental Table IV shows the certainty of evidence.

#### 3.3.3 Potential confounding factors

Some of the studies included in this meta-analysis had moderate to high heterogeneity (I^2^ > 50%); hence we performed a meta-analysis to identify other potential factors than IVH and HC that could affect the rates of mortality and good functional outcome in all ICH patients (Table 1).

**Table 1:** Confounding factors affecting 30-day and 90-day mortality.

Factors	30-days mortality					90-days mortality				
	No. of studies	Dead	Alive	Risk Ratio [95% CI], p-value	Heterogeneity (I^2^), p-value	No. of studies	Dead	Alive	Risk Ratio [95% CI], p-value	Heterogeneity (I^2^), p-value
Sex										
Male	5^22^^,^^23^^,^^25^^,^^26^^,^^31^	163	521	0.84 [0.69, 1.02], 0.07	0%, 0.62	3^26^^,^^27^^,^^30^	243	523	1.05 [0.87, 1.27], 0.60	0% 0.88
Female		119	282				112	251		
**Comorbidities**										
Hypertension										
Yes	4^22^^,^^23^^,^^25^^,^^26^	174	480	1.13 [0.90, 1.42], 0.28	34%, 0.21	3^26^^,^^27^^,^^30^	219	448	1.11 [0.93, 1.32], 0.26	0%, 0.59
No		77	250				136	326		
Diabetes										
Yes	4^22^^,^^23^^,^^25^^,^^26^	94	135	1.87 [1.52, 2.29], **<0.00001**	74%, **0.009**	3^26^^,^^27^^,^^30^	105	200	1.13 [0.94, 1.36], 0.19	92%, <0.00001
No		157	595				250	574		
Coagulopathy										
Yes	3^23^^,^^25^^,^^26^	85	79	1.99 [1.57, 2.53] **<0.00001**	56%, 0.11	2^26^^,^^30^	67	62	1.70 [1.36, 2.12] **<0.00001**	55% 0.14
No		104	290				255	641		
Smoking										
Yes	3^23^^,^^25^^,^^26^	71	104	1.32 [1.04, 1.66], **0.02**	67%, 0.05	2^26^^,^^30^	67	107	1.20 [0.97, 1.50], 0.10	0%, 0.390
No		118	265				255	596		
Alcohol abuse										
Yes	2^23^^,^^25^	26	43	1.24 [0.86, 1.80], 0.25	42%, 0.19	–	–	–	–	–
No		133	236				–	–		
**Location of hemorrhage**										
Infratentorial	3^22^^,^^23^^,^^25^	32	1	0.78 [0.56, 1.08], 0.13	0%, 0.73	2^27^^,^^30^	42	70	1.27 [0.98, 1.65], 0.07	0% 0.36
Supratentorial		204	535				254	610		
**Site of hemorrhage**										
Lobar										
Lobar	3^22^^,^^23^^,^^25^	89	202	1.29 [1.03, 1.62], **0.02**	0% 0.40	–	–	–	**-**	–
Other		132	438				–	–		
Basal ganglia										
Basal ganglia	3^22^^,^^23^^,^^25^	66	206	0.82 [0.65, 1.05], 0.12	70%, **0.04**	–	–	–	**-**	–
Other		155	434				–	–		
Thalamus										
Thalamus	3^22^^,^^23^^,^^25^	39	112	0.96 [0.72, 1.28], 0.78	0%, 0.82	–	–	–	**-**	–
Other		182	528				–	–		
Cerebellar										
Cerebellar	3^22^^,^^23^^,^^25^		66	0.93 [0.65, 1.35], 0.72	0%, 0.93	–	–	–	**-**	–
Other		189	585				–	–		
Pons										
Pons	3^22^^,^^23^^,^^25^	10	51	0.71 [0.40, 1.26], 0.24	0%, 0.83	–	–	–	**-**	–
Other		211	589				–	–		
**Initial GCS score**										
≤ 8	2^22^^,^^31^	70	118	5.20 [3.28, 8.24], **<0.00001**	0%, 0.37	–	–	–	**-**	–
>8		23	311				–	–		
Score mean difference ± st.dev (number of patients)	2^23^^,^^26^	8.53 ± 2.64 (126)	14.34 ± 1. (2)	−5.81 [-6.30, −5.33], <**0.00001**	59%, 0.12	2^26^^,^^28^	8.60 ± 3.31 (185)	12.85 ± 2.18 (410)	−4.25 [-4.79, −3.72], **<0.00001**	42%, 0.19
**ICH volume**										
>30	4^23^^,^^25^^,^^31^	171	292	2.31 [1.83, 2.92], **<0.00001**	89%, **<0.00001**	–	–	–	**-**	–
≤30		80	421				–	–		
**Midline Shift**										
Absent	3^22^^,^^23^^,^^26^	73	131	2.06 [1.63, 2.60], **<0.00001**	67%, 0.05	–	–	–	**-**	–
Present		115	438				–	–		
**Surgery**										
EVD										
Yes	–	–	–	**-**	–	2^27^^,^^30^	50	44	2.08 [1.64, 2.63]. **<0.00001**	0% 0.99
No		–	–				249	666		
Hematoma evacuation										
Yes	–	–	–	**-**	–	2^27^^,^^30^	48	115	0.99 [0.77, 1.29], 0.96	39% 0.20
No		–	–				251	595		

∗References of the studies are written in superscript inside the square brackets [].

#### 3.3.4 Confounding factors on mortality

Diabetes mellitus significantly increases 30-day mortality risk (RR 1.87 [95% CI 1.52, 2.29], p < 0.00001, I^2^ = 74% (p = 0.009)), but its impact on 90-day mortality risk is statistically insignificant (RR 1.13 [95% CI 0.94, 1.36], p = 0.19, I^2^ = 92% (p < 0.0001)). Coagulopathy significantly increases both 30-day (RR 1.99 [95% CI 1.57, 2.53], p < 0.00001, I^2^ = 56% (p = 0.11)) and 90-day mortality risks (RR 1.70 [95% CI 1.36, 2.12], p < 0.00001, I^2^ = 55% (p = 0.14)). Smoking history significantly increases 30-day (RR 1.32 [95% CI 1.04, 1.66], p = 0.020, I^2^ = 67% (p = 0.05)) mortality, but not 90-day mortality (RR 1.20 [95% CI 0.97, 1.50], p = 0.10, I^2^ = 0% (p = 0.390)).

Likewise, hypertension increases both 30-day (RR 1.13 [95% CI 0.90, 1.42], p = 0.07, I^2^ = 34% (p = 0.21)) and 90-day mortality risks (RR 1.11 [95% CI 0.93, 1.32], p = 0.26, I^2^ = 0% (p = 0.59)), but these increases are not statistically significant.

Other factors identified to increase the risk of 30-day mortality in ICH patients are lobar hemorrhage location (RR 1.29 [95% CI 1.03, 1.62], p = 0.02, I^2^ = 0%, p = 0.40), initial Glasgow Coma Score (GCS) ≤ 8 (RR 5.20 [95% CI 3.28, 8.24], p < 0.0001, I^2^ = 0%, p = 0.37), ICH volume >30 ml (RR 2.31 [95% CI 1.83, 2.92], p < 0.00001, I^2^ = 89%, p < 0.00001) and the presence of midline shift (RR 2.06 [95% CI 1.63, 2.60], p < 0.00001, I^2^ = 67%, p = 0.05). Interestingly, patients who underwent External Ventricular Drainage (EVD) placement surgery had a 2.08 times higher risk of 90-day mortality than those who did not. Conversely, there is no increase in the mortality risk in patients with hematoma evacuation (RR 0.99 [95% CI 0.77, 1.29], p = 0.96, I^2^ = 39%, p = 0.20).

Initial GCS score is significantly lower in patients who died within 30-day and 90-day periods, with a mean initial GCS score of 8.60. Patients who survived within 30-day and 90-day periods had 5.81 and 4.25 higher GCS points, respectively.

#### 3.3.5 Confounding factors on good functional outcome

Meta-analysis identified one confounding factor that significantly affected the 6-month good functional outcome, while no confounding factors affecting the 3-month good functional outcome were identified (Table 2). On the rate of good functional outcome at 6-month follow-up, initial GCS score ≤8 significantly decreases the probability of obtaining good functional outcome (RR 0.48 [95% CI 0.24, 0.97], p = 0.040, I^2^ = 0%, p = 0.34).

**Table 2:** Confounding factors affecting 3-month and 6-month good functional outcome.

Factors	3-month Good Functional Outcome					6-month Good Functional Outcome				
	No. of studies	Good	Poor	Risk Ratio [95% CI], p-value	Heterogeneity (I^2^), p-value	No. of studies	Good	Poor	Risk Ratio [95% CI], p-value	Heterogeneity (I^2^), p-value
Sex										
Male	–	–	–	–	–	2^31^^,^^33^	29	113	1.04 [0.60, 1.80], 0.88	0% 0.71
Female		–	–				14	60		
**Initial GCS score**										
≤ 8	–	–	–	–	–	2^31^^,^^33^	7	20	0.48 [0.24, 0.97], **0.040**	0%, 0.34
>8		–	–				35	25		

## 4 Discussion

Despite advanced medical and surgical strategies in treating spontaneous ICH, mortality and morbidity of ICH patients remained high. This suggests the existence of essential factors outside standard parameters, such as the ICH score, that could influence the outcome of patients. Among many suspected factors, the intraventricular extension of hemorrhage (IVH) and hydrocephalus (HC) have been independently associated with ICH patient outcomes in separate studies.^35^, ^36^, ^37^ Regardless, no meta-analysis has ever been published to suggest a consensual conclusion on how these ICH complications would affect the mortality and morbidity of ICH patients. Moreover, it is interesting to notice that although HC can be considered a direct consequence of IVH, no ICH prognostic scoring system includes HC as one factor influencing ICH patient outcomes. For example, the most extensively used ICH score, also known as the original ICH (oICH) score, includes GCS at admission, ICH volume, presence of IVH, infratentorial hemorrhage, and age. Dynamic ICH (dICH) score only adds hematoma expansion and IVH growth. Another ICH prognostic scoring system, the ultra-early ICH (uICH) score, adds CT imaging characteristics into factors considered in the original ICH score, including blend sign, black hole sign, and island sign.^17^ Additionally, another ICH prognostic scoring system called the maximally treated ICH (Max-ICH) score adds the NIHSS score, use of oral anticoagulant, lobar, and nonlobar ICH volume into factors included in the ICH score.^16^ Thus, in addition to obtaining conclusions about the significance of IVH and HC in the prognosis of ICH patient outcomes, this study also aimed to provide the scientific foundation to consider the inclusion of HC in future ICH prognostic scoring systems.

Through robust filtering processes according to PRISMA guidelines, 13 studies were selected for this meta-analysis. Mortality rates were evaluated at 30 days and 90 days after hospital admission, and functional outcomes were evaluated at three months and six months after hospital admission. Separate meta-analysis was performed for every comparison, including ICH vs ICH ​+ ​IVH, ICH vs ICH ​+ ​IVH ​+ ​HC, and ICH ​+ ​IVH vs ICH ​+ ​IVH ​+ ​HC.

The results of this study indicate that ICH ​+ ​IVH patients have a 2.19 and 1.58 times higher risk of mortality at 30-day and 90-day follow-ups, respectively, compared to patients who are only diagnosed with ICH. In line with the commonly used original ICH score, these findings reinforce the statement that the presence of IVH is an independent predictor of mortality.

When ICH progresses into HC in ICH patients, the mortality risks are 4.26 times and 2.30 times higher at 30-day and 90-day follow-ups, respectively, compared to ICH-only patients. To independently evaluate the effect of hydrocephalus on ICH and IVH patients, the mortality rate of patients with ICH ​+ ​IVH was compared to patients with ICH ​+ ​IVH ​+ ​HC. As expected, the mortality risk in hydrocephalus patients is 1.96 times higher at 30-day follow-up and 1.38 times higher at 90-day follow-up than in patients with ICH ​+ ​IVH. It is important to acknowledge that these data partially agree with a previous study's conclusion that the presence of IVH per se does not influence mortality or functional outcome but rather through the development of hydrocephalus.^38^

Due to the heterogeneity of studies, we performed further analysis on the potential impact of comorbidities, clinical presentation, and surgical intervention on the risks of mortality and good functional outcome. The association of diabetes mellitus, coagulopathy, and smoking history with ICH patient mortality was significant.^39^, ^40^, ^41^, ^42^ We speculate that these factors might have a role in damaging the cerebral blood vessels, increasing the severity of the ICH, or that the impaired vasculature might delay the recovery process in these patients. Furthermore, results from the meta-analyses also showed that the initial GCS score <8, ICH volume >30 ml, and midline shift increase the risks of 30-day mortality. This indicates that the initial GCS score reliably predicts short- and long-term patient survival. The large volume of ICH (>30 ml) directly correlates to the presence of midline shift, as a larger volume directly causes higher intracranial pressure. Investigation on how these potential confounding factors affect the risks of mortality among ICH patients is beyond the scope of this study; however, it is safe to hypothesize that larger ICH volume causes higher intracranial pressure, leading to the presence of midline shift, causing lower initial GCS, thus increasing the risk of mortality.

Moreover, it is interesting to notice that the mortality risk in ICH patients who underwent surgery to place external ventricular drainage (EVD) is 2.08 times higher than in those who did not. We speculate that the fatal outcome was not due to the EVD placement but to hydrocephalus requiring emergency surgery. This hypothesis is in line with the main findings of this study that suggest the significance of hydrocephalus in ICH patients' mortality rates.

IVH reduced the likelihood of good functional recovery to 0.83 times (at 3 months) and 0.64 times (at 6 months). In contrast, patients diagnosed with ICH ​+ ​IVH ​+ ​HC experienced a rate of good functional recovery 0.66 times (at 3 months) and 0.38 times (at 6 months) lower than those who were only diagnosed with ICH. Unfortunately, due to the insufficient number of included studies, it was impossible to calculate publication bias and small study effects, and the quality of the available evidence was either very low or very low.

To independently evaluate the effect of hydrocephalus on the rates of good functional recovery in ICH patients, a meta-analysis comparing the rates of good functional recovery of patients with ICH ​+ ​IVH to patients with ICH ​+ ​IVH ​+ ​HC was performed. Patients with hydrocephalus had 0.76 times (at 3 months) and 0.54 times (at 6 months) lower probability of gaining good functional recovery than patients with ICH ​+ ​IVH. Moderate data heterogeneities were acknowledged in the analyses at 3 months and 6 months outcomes, suggesting that other confounding factors might play roles in determining functional recovery in these patients. Thus, separate meta-analyses were conducted. From studies reporting the rates of good functional outcomes in ICH patients, pooled analysis can only be performed on two confounding factors, including sex and initial GCS score. Of these 2, only initial GCS <8 was shown to be significantly affecting the rates of good functional outcomes in ICH patients.

Interestingly, we acknowledged that the definition of hydrocephalus was not always mentioned in every included publication. We assumed that the simple definition of hydrocephalus as a progressive enlargement of ventricles was applied in these studies. However, we also noticed that there might be trivial differences in the details of hydrocephalus characteristics between studies. For example, in one study, hydrocephalus diagnosis was based on ventricle enlargement, trans ependymal edema, and basal cistern effacement.24 In contrast, in 2 studies, hydrocephalus is defined as the enlargement of lateral ventricles without extension of cisternal or sulcal spaces.^23^^,^^26^

The possible impact of these differences on the actual prognosis of patients with ICH ​+ ​IVH ​+ ​HC could not be dismissed; however, it is safe to say that the identification of enlarged ventricles in patients who are also diagnosed with ICH ​+ ​IVH suggests worse prognosis. Thus, preliminary anticipation of treating hydrocephalus as early as possible might offer better outcomes in these patients. Although it sounds logical to accept HC as one of the factors that influence the outcome of ICH patients, the exact mechanism of how HC leads to higher mortality and morbidity remains elusive. We speculate that two possible mechanisms might underlie this. First, HC might directly worsen the condition of ICH ​+ ​IVH patients. Secondly, it is also possible that the presence of HC represents other parameters worse in patients with ICH ​+ ​IVH ​+ ​HC than in patients with ICH or ICH ​+ ​IVH. For example, it is intriguing to hypothesize that the volume of hematoma in ICH ​+ ​IVH patients who developed HC was higher than in ICH ​+ ​IVH patients who did not develop HC.

Several experimental studies might hint at how IVH and HC after IVH lead to a worse prognosis. Erythrocytes, a significant blood component, are released into the ventricular system following IVH. Some neurotoxic components, including Hemoglobin (Hb), iron, peroxiredoxin-2 (Prx2), and carbonic anhydrase-1, are released into the CSF when they lyse. These elements, along with plasma proteins like thrombin, can contribute to inflammation that results in secondary brain injury and possibly PHH, as seen in ICH, SAH, and IVH models.^43^, ^44^, ^45^, ^46^ Hb from lysed erythrocytes is a strong pro-inflammatory stimulant. Through Fenton reactions, the iron released when Hb breaks down can harm nearby tissues through oxidative stress. Through iron-induced JNK signaling pathways, structural damage was observed in the nearby choroid plexus and hippocampus after IVH.^47^ Iron may also contribute to fibrosis of the ventricles and CSF hypersecretion, in addition to causing brain damage via reactive oxygen species. According to Qing et al, iron caused ICH-induced brain edema by upregulating aquaporin4 (AQP4).^48^ In an adult IVH model, deferoxamine, an iron chelator, significantly decreased the risk of hydrocephalus in 20% of rats receiving whole blood injections and 10% receiving iron injections.^49^ Koduri et al have outlined several animal-based and human-based studies which demonstrate the effect of iron and iron chelation therapies in stroke patients, which is a very promising avenue of study for the future.^50^

The coagulation cascade is immediately activated following a hemorrhage. Thrombin, also known as factor IIa, causes blood clotting by cleaving fibrinogen into fibrin. A single intraventricular thrombin injection significantly increased hydrocephalus, damaged the ventricular wall and disrupted the blood–brain barrier. Poor functional outcome at six weeks and six months was associated with increased thrombin activity and concentration.^51^

The brain's resident immune cells, microglia, and invading macrophages become active after hemorrhage. The surrounding brain tissues can be harmed by the release of pro-inflammatory cytokines, extracellular proteases, and oxidative species by resident microglia and recruited macrophages in addition to the phagocytosis of erythrocytes and their debris.^52^ From these studies, we can conclude that the primary mechanism of poor prognosis after IVH and HC is due to increase inflammation and brain tissue necrosis.

Increased intracranial pressure causes decreased brain compliance and changes in the brain's cardiac-induced pulsatility, which are the two factors that contribute to HC's worse prognosis. Lack of nutrients, an accumulation of metabolic waste, and oxygen deprivation of the neurons are all consequences of reduced pulsatility.^53^

## 5 Conclusion

The presence of IVH in ICH patients decreases patient survival and good functional outcome. Furthermore, the direct consequence of IVH: hydrocephalus, worsens the ICH patient's outcome. Thus, it is logical to suggest the inclusion of hydrocephalus in future ICH prognostic scoring systems. Separate evaluation using multivariate logistic regression with the addition of IVH Graeb score^54^ and/or Diringer HC score^55^ in the widely used ICH prognostic score warrants further study.

## Statements

### Statement of ethics

An ethics statement is not applicable because this study is based exclusively on published literature.

### Conflict of interest statement

The authors declare no competing interest.

### Funding sources

This research did not receive grants from public, commercial, or not-for-profit funding agencies.

### Author contributions

POW: conceptualization, data curation, validation, supervision, writing-review, editing, and funding acquisition; ABS: conceptualization, validation, supervision, writing-review, and editing; DH: conceptualization, methodology, formal analysis, data curation, writing-original draft, writing-review, and editing; JKA: conceptualization, methodology, formal analysis, data curation, writing-original draft, visualization, writing-review, and editing; PPL: statistical-analysis, writing-original draft, writing-original draft; AA: conceptualization, validation, supervision, writing-review, and editing; YTT: conceptualization, validation, supervision, writing-review, and editing; EJP: conceptualization, validation, supervision, writing-review, and editing; JJ: conceptualization, validation, supervision, writing-review, and editing; THA: conceptualization, validation, supervision, writing-review, and editing.

### Data availability statement

This article and its supplementary material files include all data generated or analyzed during this study. Further inquiries can be directed to the corresponding author.
