# Supplementary material for: Hydrocephalus is an independent factor affecting morbidity and mortality of ICH patients: Systematic review and meta-analysis
Source: World Neurosurg X. 2023 Apr 10;19:100194. doi: 10.1016/j.wnsx.2023.100194 (PMC10288487; doi:10.1016/j.wnsx.2023.100194)
Supplement: Multimedia component 1 [file mmc1.docx]

**Supplemental Table I.** Characteristic of Eligible Studies

| **Author, country, year** | **Grouping**  **(No. of patients)** | **Type** | **Inclusion and Exclusion Criteria** | **Reported Outcomes** | **Comorbidities** | **Intervention** | **Site of hemorrhage** | **Other variables** | **Author conclusion** | **Reason for Exclusion** |
| --- | --- | --- | --- | --- | --- | --- | --- | --- | --- | --- |
| Al Safatli 2017^1^  Germany | 50  GCS<10  GCS >10 | Single center retrospective | Inclusion: patients diagnosed with a first episode of isolated cereberal SCH  Exclusion: secondary cerebellar hemorrhage caused by trauma,  tumours, cavernomas, arteriovenous malformations or  aneurysms, hemorrhagic transformation of a cerebellar  infarct, the presence of accompanying supratentorial or  brainstem hemorrhage, incomplete medical records and  patients with initial absence of brainstem reflexes on  admission. | 30-days mortality, unfavourable outcome (mRS > 3) | Hypertension | Conservative, EVD alone, Hematoma evacuation + EVD, use of AP, use of AC | Unilateral, midline, difuse | Presence of IVH, presence of HC | Lower GCS score on admission was associated with increased 30‑day  mortality and poorer short‑term outcome in patients with SCH. For patients with a  GCS score <10 on admission, it is important to balance the possibility of survival  afforded by further therapy against the formidable risk of significant functional  disability and poor quality of life. |  |
| Asadollahi 2016^2^  Iran | Total (228)  IVH (68)  HC (36) | Multi-center study  Prospective | The inclusion criteria were: (1) **first-ever SICH**, (2)  age between 40–85 years and (3) admission within 6 hours of  symptoms onset. Patients with a secondary cause for their  haemorrhage, such as aneurysmal rupture, haemorrhagic  transformation of ischaemic stroke, head trauma, vascular  malformation, tumour, cerebral venous thrombosis or previous operation were excluded | 30-days mortality, mRS, Barthel Index, 36 months | Hypertension  Diabetes mellitus  Dyslipidemia  DVT  Peripheral vascular disease  Coronary artery disease  Smoking  Alcohol abuse  Antiplatelet | Not stated | Lobar, basal ganglia, intraventricular, thalamus, cerebellum, brain stem | Neurological state on arrival (GCS,ICH, NIHSS), hemorrhage side, hemorrhage volume, midline shift | A favourable long-term functional outcome at 36 months and short-term survival  were less likely in patients with greater volume of haematoma, presence of IVH and midline  displacement | Not reporting hydrocephalus |
| Appelboom 2011^3^ | Total (104) | Single center  Prospective | patients with spontaneous ICH diagnosed by admission CT scan were admitted to the  Columbia University Medical Center Neurological Intensive Care  Unit and prospectively enrolled in the Intracerebral Hemorrhage  Outcomes Project (ICHOP)  Exclusion: Patients 18 years;  patients with ICH due to malignancy, trauma, hemorrhagic conver sion, or another primary bleeding event; and patients with incomplete data were excluded from the study analysis. | Discharge mortality  3-mo mortality | Diabetes mellitus  Hypertension | EVD, shunt, Intrathecal tPA, hematoma evacuation | Supratentorial  Infratentorial | Admission IVH score  Admission ehmatoma size  Admission GCS  Midline shife  Admission glucose | Admission hyperglycemia after spontaneous ICH is associated with poor outcome and potentially related to  the presence and severity of intraventricular extension. |  |
| Bakhshayesh 2014^4^  Iran | Total (98)  IVH (43) | Single center Prospective | All patients presented to the emergency  department (ED) within the first 24 h of acute onset of  focal neurological deficit whose brain Computed  tomography (CT) scan on admission was compatible  with a diagnosis of ICH were included in the study.  Exclusion criteria were: (1) evidence of head trauma, (2)  concomitant epidural or subdural hematoma, (3) history  of stroke, bleeding tendency disorders, dementia, cancer,  or any other severe concomitant illness, (4) secondary  ICH (e.g., vascular malformations, aneurysm, tumor,  trauma, vacuities, etc.), (5) neurosurgical intervention,  and (6) transfer to another facility | In-hospital mortality | Coronary artery disease  Hypertension  Diabetes mellitus | Conservative | Thalamus, basal ganglia, lobar, cerebellar, brainstem | Mean GCS, NIHSS, hematoma volume, and volume of PHE | Our results indicate that older age, diabetes  mellitus, higher NIHSS, as well as larger volume of  hematoma, and smaller PHE on admission are important  predictors of in-hospital mortality in our ICH patients | Uncertain period of mortality |
| Bhatia, 2013^5^  India | Total 214  IVH (53) HC(74) | Single center  Prospective | Between June 2008 and June 2012, all consecutive patients  of spontaneous ICH admitted under stroke services of the  neurology department at All India Institute of Medical  Sciences, New Delhi, were recruited. We excluded  patients with subdural and epidural hematoma,  aneurysmal, arteriovenous malformation (AVM),  anticoagulant or coagulopathy-related hemorrhage and  patients who denied informed consent | In-hospital mortality | Hypertension  Diabetes  Dyslipidemia  Smoking  Alcohol  Past stroke | Mechanical ventilation, neurosurgical, hematoma evacuation | Supratentorial, infratentorial | Hemorrhage volume, midline shift >6mm | Low GCS, higher baseline ICH volume, presence of IVH and need for  ventilatory assistance are independent predictors of mortality. Most of the patients at  discharge were disabled. Surgery did not improve mortality or outcome. | Uncertain period of mortality |
| Bhattahiri et al., 2006^6^  UK | Total (n=1033)  No IVH ICT  no IVH ES  IVH ES  IVH ICT  IVH + HcP ES  IVH + HCP ICT | Multicenter study  Randomized trials | Patients were eligible for inclusion if they  had CT evidence of a spontaneous supratentorial  intracerebral haemorrhage that had arisen within 72 h  and if the responsible neurosurgeon was uncertain  about the benefits of either treatment (the clinical  uncertainty principle).  Patients were not eligible if: the haemorrhage was  probably due to an aneurysm or an angiographically  proven arteriovenous malformation; the haemorrhage  was secondary to a tumour or trauma; patients had a  cerebellar haemorrhage or extension of a supratentorial  haemorrhage into the brainstem; patients had severe  pre-existing physical or mental disability or severe  comorbidity that might interfere with the assessment of  outcome; surgery could not be undertaken within 24 h of  randomisation. | Favorable outcome (8-point Glasgow Outcome Score), | No comorbidities | Early surgical vs conservative | Lobar, basal ganglionic, both | Midline shift, hematoma volume, side of hematoma, primary site | The presence of IVH and hydrocephalus are independent predictors of poor outcome in spontaneous ICH. Early surgery  is of some benefit in those with IVH |  |
| Buensuceso et al., 2007^7^ | Total (21)  IVH, subarachnoid, hydrocephalus | Retrospective Single center | upon admission, cranial CT scan done revealed  findings of hypertensive intracerebral hemorrhage.  Included were all charts of patients in this study reveal  admitting diagnosis of hypertensive intracerebral  hemorrhage either managed medically or with surgical  intervention. Excluded were the following: Patients  with intracerebral bleed secondary to causes other than  hypertension such as trauma, ruptures AV malformation/  aneurysm, tumoral bleed, or impaired coagulation;  patients who were initially admitted at our hospital and  with cranial CT scan done but eventually transferred to  another hospital for further management and patients who  were discharged against medical advice. | In-hospital mortality | Not specified | Medical and surgical | Not stated | Midline shift, volume hematoma | The presence of subarachnoid hemorrhage in the CT scan findings of patient admitted due to HICH  gives three times higher risk of dying to the patient, thus a good predictor of mortality. | Uncertain period of mortality |
| Celikbilek et al., 2013  Turkey^8^ | Total (106)  IVH (39) | Single center  Retrospective | those with ICH secondary to primary or  secondary intracranial tumor, those with traumatic  or hemorrhagic ischemic (arterial/venous) etiologies  and those with subarachnoid or subdural hemorrhages  were excluded from the study. | In-hospital mortality | Smoking, alcohol, DM, HL, HT | Medical and surgical (no data stratification) | Not stated | Bleeding volume, ventricular extension of hemorrhage, midline shift | The results of this study suggest that eﬀective control of blood pressure  is important in the prevention of spontaneous ICH; clinical and radiological fndings with treatment modalities  inﬂuencing mortality should be carefully managed | Uncertain period of mortality |
| Chan et al., 2015^9^  International | Total (2613)  ICH with IVH (740)  ICH without IVH (1873) | Randomized controlled trial  International | In brief, 2839 patients with computed  tomographic–confrmed spontaneous ICH within 6 hours of onset  and elevated systolic blood pressure (systolic BP, 150–220 mm Hg)  were randomly assigned to receive intensive (target systolic BP, <140  mmHg within 1 hour) or guideline-recommended (target systolic BP,  <180 mm Hg) BP-lowering therapy using locally available agents according to standardized protocols.  Patients were excluded if there was a structural cerebral cause for the intracerebral hemorrhage, if they were in a deep coma (defined as a score of 3 to 5 on the Glasgow Coma Scale [GCS], if they had a massive hematoma with a poor prognosis, or if early surgery to evacuate the he-matoma was planned. | 90-days mortality, 90 days major disability  Days of hospitalization  Serious adverse events (neurological deterioration, recurrent cardiovascular event, noncardiovascular) | Hypertension, heart disease, diabetes mellitus, prior ICH, ischemic or undifferentiated stroke, warfarin anticoagulation | Hemostatitc therapy, mannitol, surgical intervention (evacuation, insertion) | Not stated | Intraventricular hemorrhage volume | A strong association exists between the amount of IVH and poor outcome in intracerebral hemorrhage. An  IVH volume of 5 to 10 mL emerges as a signifcant threshold for decision making on prognosis in these patients. | Uncertain case of IVH without hydrocephalus |
| Chaturbedi et al., 2020^10^  Nepal | Total (75) | Single center  Prospective | Inclusion criteria: 1) Patients with spontaneous ICH due to chronic  arterial hypertension who presented to our hospital were enrolled in the  study 2) Patient who gave consent to participate in the study 3) A preset  data collection form has been duly filled and complete 4) Patient should have  follow up visit at 3, 6 and 12 months after ICH in outpatient setting 5) In case  patient fails to follow up, the patient or next of kin is phoned to know the condition  of the patient. They were approached with questionnaires that helps in  deciphering patient’s current functional status 6) Patients who were hospitalized in  another institute but subsequently brought to our hospital within 6 hours of  ictus was included.  Exclusion criteria: 1) Patient with mRS score >2 prior to ICH 2) Patients with  traumatic ICH 3) No previous adverse intracranial events (e.g. stroke, moderate to  severe traumatic brain injury and brain surgery for any pathology)  4) ICH related to intracranial tumors, vascular malformation (cerebral aneurysm,  arteriovenous malformation, cavernorma etc.) and cerebral venous sinus thrombosis  5) Patients who are taking blood thinners despite meeting the eligibility criteria as  they are identifiable compounding factor 6) Patients with isolated intraventricular  hemorrhage 8) Incomplete medical records or data collection sheet 8) Patient  lost to follow up. | Factor associated with major disability or death | Hypertension, hyperlipidemia, ischemic heart disease, chronic obstructive pulmonary disease, congestive heart disease, peripheral vascular disease, chronic liver disease, cancer | Craniotomy and evacuation clot, EVD, decompressive hemicraniectomy | Supratentorial, basal ganglia, thalamus, infratentorial: cerebellum, brain stem | Charlson comorbidity index, mRS before ICH, GCS, infratentorial location, hematoma volume, IVH, HC | SpICH from CAH is associated with high mortality, and about one  third of survivors end up with severe disability or death 3 months later. Predictors  of severe disability or death were functional disability prior to ICH, low GCS on  admission, larger hematoma volume, infratentorial location of ICH, persistently  elevated BP and intraventricular hematoma. | No data of associated factors to outcome |
| Chen et al., 2015^11^  China | *Total (128*  Dead (15)  Survivors (113)*)* | Single center  Retorspective | A total of 128 patients, aged ≥18 years, had  spontaneous ICH confrmed by computed tomography (CT)  within 6 h onset and elevated systolic BP ≥150‑220 mmHg.  Inclusion criteria for the study were: ≥18 years of age, spontaneous HICH within 6 h confrmed by CT, and elevated systolic  BP of ≥150 mmHg. Exclusion criteria for the study were: clear  evidence that the HICH was secondary to a structural cerebral  abnormality (e.g., arteriovenous malformation, intracranial  aneurysm, or tumour) or under treatment with the use of a  thrombolytic agent, or with a pre-planned decompressive  neurosurgical intervention. | Dead at 90 days | Not stated | Conservative | Basal ganglia, thalamus, lobar, cerebella, brain stem | Age, gender, hematoma volume, IVH, blood pressure, GCS score, NIHSS score | age and hematoma  volume may be important early predictors of death in HICH.  Proactive control and management of hematoma may reduce  the mortality of HICH. | Uncertain case of IVH without hydrocephalus |
| Cheung et al., 2003^12^  China | Total (142)  Fatal outcome (31)  Bad outcome (61)  Good outcome (49) | Single center  Prospective | Patients admitted via the emergency department with nontraumatic  ICH in 1999 were identified from our stroke database for a detailed  review of medical records and CT findings. | Patients who died before 30 days had a modified  Rankin score of 6. Good outcome was defined by a modified Rankin  score of 2 at 30 days. | Hypertension, diabetes mellitus, ischemic heart disease, atrial fibrillation, history of smoking, history of drinking | Surgical evacuation, ventricular drainage | Basal ganglia, thalamic, lobar, pontine, cerebellar, supratentorial | Age, comorbidities, GCS score, NIHSS score, admission temperature, BP, pulse, site of ICH, ICH volume, IVH, subarachnoid extension, mass effect, hydrocephalus, Hb, white cell, platelet, prothrombin, treatment | All 3 ICH Scores are simple clinical grading scales. As reliable predictors of good outcome and/or mortality,  they are useful in clinical research studies and standardization of clinical protocols. | Cause of nontraumatic ICH could also cause by structural abnormalities, not stated in the exclusion criteria |
| Chuang et al., 2009^13^  Taiwan | Total (293)  Dead (40)  Survive (253) | Single center  Retrospective | Therefore, only patients who were admitted to our hospital  for an initial evaluation of ICH were included. Patients with  hematologic or coagulation disorders were excluded to avoid  a selection bias. | 30-days mortality | HT  DM  ICD  Stroke  Dialysis | Not stated | Supratentorial (basal ganglia, thalamus, lobar, multiple), infratentorial (cerebellum, pons) | BP, pulse, location of ICH, site of ICH, age, ICH volume (30mL), IVH, pineal shift, hydrocephalus | The sICH score showed best discrimination among tested models. Also, it was easier for physicians without  special training in neurology or radiology to use this scale. With statistical power and ease of use, the sICH score is a very  suitable model for risk stratification of spontaneous ICH | Cause of nontraumatic ICH could also cause by structural abnormalities, not stated in the exclusion criteria |
| Daverati et al., 1991^14^  France | Total (166)  Dead (71)  Alive (95) | Single center  Prospective | 1)Admission < 24 hours after stroke onset  2) CT demonstration of an ICH with or without intraventricular spread of the hemorrhage  3) **absence of an external cause for the stroke such as head injury, anticoagulant therapy, or a definite intracranial source of hemorrhage that could necessitate surgery** (aneurysm, arteriovenous malformation, or tumor) | 30-day and 6-month mortality | Not stated | Conservative | Putamen thalamus lobus | Age, gener, hemorrhage size, hemorrhage location, hemorrhage size, midline shift, IVH, initial level of conciousnes, limb paresis, oral | Early (30-day) survival was correlated with morphologic parameters on the initial computed tomogram (hemorrhage size, midline shift, and intraventricular spread of the hemorrhage), while later (6-month) survival was correlated with age. Using logistic regression, we found five independent predictors of satisfactory outcome at 6 montsh: age, hemorrhage size, intraventricular spread of the hemorrhage, limb paresis, and communication disorders. Of these, age was the most important | Uncertain case of IVH without hydrocephalus |
| Di Napoli et al., 2011^15^ | Total = 210  Alive = 147  Dead = 63 | Single center  Retrospective | **Inclusion:**  + sICh patients admitted to ICU 2005-2009  + Absence of trauma  + Absence of structural lesion  **Exclusion:**  + History of acute or chronic infections | 30-day mortality | + Arterial hypertension  + Diabetes mellitus  + Alcohol  + Smoking  + Cholesterol  + Antiplatelet | Surgery and conservative | SUpratentorial infratentorial | Age, gender, surgery, ICH volume, IVH, GCS score, ICH score |  |  |
| Diringer et al., 1998^16^ | Total (81)  Hydrocephalus (40)  No HC (41) | Single center  Prospective | The database was searched to identify all patients  admitted with a primary diagnosis of supratentorial ICH over a  20-month period. Patients were excluded if the hemorrhage was  associated with trauma or subarachnoid hemorrhage or if a CT scan  performed within 24 hours of the hemorrhage was not available. | hospital disposition: dead, nursing home, rehabilitation, home  3-month follow-up FIM | Hypertension  Diabetes | Not specified | Not stated | Age, race, gemder, comorbidities, GCS score, pulse pressure, MAP | We conclude that hydrocephalus is an independent predictor of mortality after ICH | Uncertain period of hospital disposition  No available number of patients data for 3-month outcome |
| El-Saadany et al., 2012^17^  Egypt | Total (54)  HC (18) No-HC (36) | Single center | This study included 54 adult patients with computed  tomography (CT)-proven IVH admitted to the neurosurgical casualty or referred from the medical casualty  department at Alexandria main university hospital | Correlation of severity of IVH and development of HC | Hypertension, diabetes, AVM, aneurysm, coagulophaty, trauma | Not stated | Not stated | Age, primary, secondary hemorrhage | We concluded that IVH per se is not as serious as it  was presented in earlier studies compared with other types of  intracranial hemorrhages, and it seldom has a major effect on  the neurological outcome. | Mix with aneurysm and coagulopathy, trauma ethiology |
| Eslami et al., 2019^18^  USA | Total (467)  Location: thalamus (332)  Non-thalamus (135)  Caudate (219)  PLIC (188)  ALIC (108)  Gp/P (127)  Lobar (29)  EVD + IV | Multicenter, randomized placebo controlled trial | The main inclusion criteria were: (1) age 18 to 80 years, (2)  spontaneous (hypertensive) ICH with hematoma volume <30 mL, (3) obstruction of the third  and/or fourth ventricles, (4) presentation within 24 hours of symptom onset, (5) stability of  ICH, IVH, and any EVD tract hemorrhage prior to 72 hours from diagnostic non-contrast  computed tomography (CT) scan, and (6) baseline modified Rankin score (mRS) <2. | 30 and 180 days mortality  30 and 180 days functional outcome (mRS 4-6)  30 and 180 days independent living (NIHSS >4, SIS <60, BI<86)  VAS for health perception | Not stated | No comparison only EVD | Thalamuse, caudate, PLIC, ALIC, GP/Putamen, lobar | ICH volume, IVH volume, initial mRS, GCS | Acute ICH lesion topography provides important insights into anatomic correlates  of mortality and functional outcomes even in severe IVH causing obstructive hydrocephalus.  Models accounting for ICH location in addition to volumes may improve outcome prediction and  permit stratification of benefit from aggressive acute interventions | No comparison outcome between ICH and ICH+IVH. All cases = ICH+IVH |
| Giray et al., 2009^19^  Turkey | Total (24) | Single center  Retrospective | We defined PIVH  as hemorrhage detected by CT in the ventricular  system only. We excluded patients with  intraparenchymal hemorrhage, even if the  hemorrhage was small or very close to the  ventricular system, and also patients with  intracerebral hemorrhage related to trauma or with  subarachnoid hemorrhage. | In- hospital mortality, memory problem, IVH score | AVM, HT, anticoagulant | Early surgery, EVD | Not stated | Age, GCS/FOUR on admission, risk factor | Hypertension is the most common associated risk factor for PIVH  followed by vascular malformation. Spontaneous resorption and rebleeding may be seen.  The neurological status of the patients and an early developing hydrocephalus are the most  important risk factors. | Primary IVH |
| Godoy et al., 2006^20^  Argentina |  | Single center  Prospective | SICH was defined as a neurological deficit documented by a brain  computed tomography (CT) indicating the presence of an ICH in  absence of trauma or surgery.1 Admissions fulfilling 1 of the  following criteria were excluded: patients with hemorrhage secondary to brain tumors, to trauma, to hemorrhagic transformation of  cerebral infarct, or to aneurysmal or vascular malformation rupture.  Patients evaluated 24 hours after symptom onset together with  patients referred directly from another hospital after diagnosis and  initial evaluation were also excluded. | 30-Day mortality  6-month good outcome (GOS 4-5) | Not specified | Surgery vs non surgery | Supratentorial (basal ganglia, thalamic, lobar), infratentorial (pontine, cerebellar) | Age, gender, comorbidities, initial GCS, ICH volume (<30, 30-50, >50), Graeb’s score, IVH, surgery | oICH score also confirms its validity in a socially and culturally different population. Modifications of  oICH do not improve its 30-day mortality prediction but improve its ability to predict good functional outcome at 6  months. | Uncertain case of IVH without hydrocephalus |
| Grand et al., 2015^21^  USA | Total (250) | Single center  Retrospective | all cases of adult patients (.17 years of age) with  hydrocephalus in whom an ETV procedure was performed at our center  between January 2000 and June 2014 (14.5 years). The categories  consisted of aqueduct stenosis, intraventricular hemorrhage (IVH),  communicating hydrocephalus, obstruction from tumor or cyst, VPS  obstruction (diagnosis unknown), and miscellaneous | Success of surgery |  |  |  |  | Use of ETV in adult hydrocephalus has broad application with a low  complication rate and reasonably good efficacy in selected patients. | Tumor, obstruction cause of hydrocephalus |
| Guo et al., 2016^22^  China | Total (50)  Surgery (40)  Conservative (58) | Single center  Retrospective | We diagnosed patients with PIVH when computed tomography  (CT) revealed hemorrhage restricted to the ventricles. Patients with  extravasation of blood from disrupted or ruptured brain  parenchyma or discernible SAH on CT scans were excluded.  After the identification of 148 patients with PIVH, 50 cases were  excluded due to underlying causes such as moyamoya disease,  AVM, aneurysms, brain tumors, and trauma confirmed using  digital subtraction angiography (DSA), computed tomography  angiography (CTA) or magnetic resonance angiography (MRA) or  because medical information was unavailable. | In hospital mortality, poor outcome (mRS >4) | Hypertension, hyperlipidemia, diabetes, drinking, smoking, family history of stroke, coagulation disorders | Surgery vs conservative | Ventricular only | Age, gender, comorbidities, admission GCS score, Graeb score | IPIVH is rare in clinical practice, and hypertension is the most common risk factor. Furthermore, the treatment of IPIVH is still  controversial. Hematoma evacuation under a microscope or an endoscope, EVD, LD and a combination of EVD and LD could be  surgical options for the treatment of IPIVH patients. The outcomes for IPIVH patients could be relatively favorable with individualized  treatment. | Primary cause of IVH |
| Hameed et al., 2005^23^  USA | Total (15) | Single center  Retrospective | Patients with non traumatic intracerebral haemorrhage (ICH), admitted at our institution from 1988 to 2001  were identified through ICD-9 coding system.  PIVH was defined as presence of blood  confined strictly to ventricular system. | In hospital mortality | Diabetes mellitus, coagulopathy, blood in all ventricles | Not stated | Ventricle only | Comorbidities | Hypertension is most common associated risk factor for primary intraventricular haemorrhage followed by coagulopathy. Hydrocephalus is a common complication, associated with poor outcome. Diabetes mellitus, coagulopathy and panventricular blood predict early mortality | Primary IVH |
| Hedge et al., 2020^24^  India, UK | Total (1052) | Multi center | All patients  above the age of 18 years who were presented to the Emergency  Department between 1st January 2015 and 31st December 2018  with computerised tomography (CT) evidence of SICH were  included in the study. Patients with post-traumatic hematomas, intracranial space-occupying lesions with bleeds, haemorrhagic transformation of an ischemic stroke, vascular  malformations, and aneurysms were excluded from the study | 90-day outcome (mRS) | Hypertension  Diabetes  Smoking  Alcohol  Previous stroke  Coagulation | Surgery: EVD, Clot evacuation | Supratentorial, infratentorial | Initial heart rate, BP, GCS score, ICH score, blood glucose, volume (>30), IVE, hydrocephalus | SICH predominantly affects a younger population in India in comparison to the Western  society. Elderly age, poor GCS on admission, clot volume above 30 ml and intraventricular extension remain the most consistent  predictors of death and poor outcome. Further studies are needed to assess the risk of SICH among hypertensive patients and to  prognosticate the outcome after SICH using novel predictors, including biomarkers. |  |
| Hemphil et al., 2001^25^  USA | Total (152) | Single center  Retrospective | A retrospective review of medical records of patients with nontraumatic ICH treated at the University of California, San Fransisco  Since the  purpose of this study was to develop prognostic criteria for use at the  time of first evaluation, patients were only included if they presented  to the emergency department at either Moffitt-Long or San Francisco  General Hospital for initial evaluation of their ICH. Patients who  were transferred from an outside clinic or hospital were not included  because these patients would not have been candidates for acute  intervention at UCSF. | 30-day mortality outcome | Hypertension, amyloid, illicit drugs, underlying lesion | Surgery: EVD, hematoma evacuation | Supratentorial (basal ganglia, lobar, thalamus), infratentorial (cerebellum, pons) | Gender, comorbidities, age, IVH, surgical treatment | The ICH Score is a simple clinical grading scale that allows risk stratification on presentation with ICH. The  use of a scale such as the ICH Score could improve standardization of clinical treatment protocols and clinical research  studies in ICH | Mix all non-traumatic ICH, can be including cancer and aneurysm |
| Herrick et al., 2014^26^  USA | Total (178)  EVD (67)  No EVD (116) | Single center  Retrospective | All patients with a primary diagnosis of ICH  (ICD-9 code 431) were identified, and those with radiographic evidence of any IVH were included. Patients were  excluded for the following reasons: craniotomy or craniectomy, aneurismal subarachnoid hemorrhage, or ICH  related to trauma or underlying lesions, including aneurysms, brain tumors, and arterio-venous malformations | In-hospital mortality (11 + 13 days [range 1-80 days]) | Not stated | EVD vs No EVD | Infratentorial, supratentorial | Age, race, volume, pulse, Graeb score | Patients with lower GCS, higher IVH  severity, and lower ICH volume are more likely to have an  EVD placed. EVD placement is associated with reduced  mortality and improved short-term outcomes in patients  with IVH after adjusting for known severity factors. EVD  use should be protocolized in clinical trials of ICH management where IVH is included. | No exact data for number of patients died in hydrocephalus group |
| Hughes et al., 2015^27^ | Total (105)  EVD (30)  No EVD (75) | Single center  Retrospective | Inclusion criteria were  patients ≥ 18 years old with IVH after ICH, trauma, tumor, or vascular pathologies. Exclusion criteria were IVH  with more than minimal subarachnoid hemorrhage (because EVD is used commonly in our practice for patients  with only mild symptoms of hydrocephalus), layering IVH  only, patients who had only 1 CT scan at presentation and  were immediately treated with EVD, ventricular trapping,  and catastrophic hemorrhages treated solely with palliative measures. | mGS score | Hypertension, diabetes, hyperlipidemia, stroke history, spontaneous ICH, trauma, tumor, vascular anomalies | EVD vs No EVD | Caudate, basal ganglia, thalamic lobar, infratentorial | Comorbidities, used of anticoagulant | In this study population, the risk for EVD was variable, but greater with mGS > 13, coma, and a dilated  fourth ventricle. While the need for EVD occurs within the 1st day after IVH in most patients, a minority require EVD after  48 hours. | IVH after other cause than ICH |
| Huttner et al., 2006^28^  Germany | Total (40)  Hematoma volume >25mL  Hematoma volume <25mL | Single center  Retrospective | First, of 1372 patients  with ICH, 412 with primary hypertensive ganglionic hemorrhage who did not undergo surgical evacuation of the hematoma were included. Patients with warfarin-related ICH  were excluded, as were those with sub- and epidural hematomas, subarachnoid hemorrhage, and ICH caused by  trauma or tumors. Next, of these 412 patients, 116 with occlusive hydrocephalus were identified in a second step, and  76 who received an EVD were identified in a third step. | mRS score (0-1), (2-3), (4-5), dead | Not stated | EVD | Not stated | Not stated | The long-term outcome of treated patients with supratentorial ganglionic hemorrhage with ventricular involvement and occlusive hydrocephalus is comparable to that of patients with similar hematoma volumes but  no IVH. | No comparison between ICH+IVH, ICH +IVH+HC |
| Hwang et al., 2012^29^  USA | Total (73)  mRS <3 (18)  mRS >3 (55)  No EVD (34)  EVD (39) | Single center  Retrospective | All patients with spontaneous ICH admitted to the  neurological intensive care unit of Columbia University  Medical Center between February 2009 and February 2011  were offered participation in the Columbia University Intracerebral Hemorrhage Outcomes Project. The study was  approved by the hospital’s institutional review board, and  in all cases, informed consent was obtained from the patient or surrogate. The diagnosis of ICH was established by  CT scan. Patients were included in the analysis if they had  spontaneous nontraumatic ICH with evidence of IVH on  CT scans upon admission and within 24 hours of onset.11  Patients were excluded if the ICH was due to secondary  causes such as trauma, aneurysm, or arteriovenous malformation rupture, or hemorrhagic conversion of an infarct. | mRS score | Not stated | Hematoma evacuation, EVD, tPA | Not stated | Median GCS score, median ICH score, ICH volume, mean IVH volume, bleeding in lt ventricle, hydrocephalus, infratentorial | The IVH, Graeb, and LeRoux scores predict outcome well with similarly good accuracy in ICH  patients with IVH when assessed at admission and within 6 days after hemorrhage. Therefore, any of one of the  scores would be equally useful for assessing IVH severity and risk-stratifying ICH patients with regard to outcome.  These results suggest that EVD placement may be benefcial for patients with severe IVH, who have particularly poor  prognosis at admission, but a randomized clinical trial is needed to conclusively demonstrate its therapeutic value | Score at discharge |
| Inagawa et al., 2003^30^  Japan | Total (350)  Putamen (120)  Thalamus (115)  Caudate (7)  Lobar (53)  Cerebellum (25)  Brainstem (30) | Single center  Retrospective | ICH was verified by  CT scanning alone in 345 patients, by magnetic resonance  imaging alone in 3, by both methods in 1, and by CT scanning  and autopsy in 1. There were no patients with confirmed  amyloid angiopathy. | Number of incidence at the specified site | Hypertension, smoking, alcohol, diabetes mellitus, cholesterol, BMI | Craniotomy, surgery with or w.o. EVD, EVD | Putamen, thalamus, caudate nucleus, lobar, cerebellum, brainstem | Comorbidities, age, dayo of admission, GCS score on admission, Volume hematoma, IVH grade, midline shift | Marked differences were observed in the incidence rates and outcome of primary ICH in relation to the site of hemorrhage. The differences in outcome  were primarily a result of differences in the severity of bleeding for each ICH subtype. | ICH only cases |
| Ironside et al., 2019^31^  USA | Total (209)  mRS 0-2 (41)  mRS 3-6 (168) | Single center  Retrospective | The inclusion criteria for this study were (1)  age 18 years; (2) baseline modified Rankin Scale  (mRS) score of 0–2 prior to ICH;19 (3) initial computed  tomography (CT) neuroimaging within 72 h of symptom onset and (4) available 90-day follow-up functional  outcomes data. Primary intraventricular hemorrhage  (IVH) and ICH related to trauma, brain tumor, hemorrhagic transformation of cerebral infarction, vascular  abnormality, or any other suspected secondary causes  were excluded from the study. | Functional outcome (mRS) 90 days | AF, CAD, hyperlipidemia, HTN, Diabetes mellitus | Conservative | Lobar, thalamus, cerebellum, brainstem | IVH present, ethnicity, gender, age | The interaction between hematoma volume and location exerted an independent effect on outcomes.  Excellent discrimination of functional dependence and mortality was observed with incorporation of location-specific  volume thresholds into a prediction model. Therefore, the volume–location relationship plays an important role in ICH  outcome prediction. | Uncertain case of IVH without hydrocephalus |
| Kim et al., 2009^32^  Korea | Total (585) | Single center  Retrospective | Patients admitted to the center between 1st  January 2004 and the 31st July 2008 . For the  purpose of this study, patients aged 40  and older were selected. We excluded  patients younger than 40, because of  the likelihood of a secondary cause of  PICH. We also excluded patients with  infratentorial hemorrhages, because in  these patients small changes in size and  location are believed to have a greater  impact on survival than is the case for  supratentorial hemorrhages.  Patients with hemorrhage secondary to head trauma, a ruptured cerebral aneurysm, an arteriovenous malformation, a tumor, bleeding diathesis, or a  hemorrhagic infarction were excluded. Patients with a severe  neurological handicap (Modified Rankin Scale43) score 4, 5)  due to previous stroke were also excluded. | 30 days mortality  90 days functional outcome (mRS): functional recovery (mRS <3) | Smoking, alcohol, body mass index | Conservative, surgery (craniotomy, craniectomy, sterotactic aspiration, ventriculostomy, combined) | Supratentorial only (basal ganglia, thalamus, lobar) | Age, consciousness during admission, pupillary abnormalities, limb weakness, GCS score, hematoma location, midline shift, blood parameter  Time to surgery | The predictors of mortality and functional recovery after PICH identified during this analysis may assist during clinical decisionmaking, when advising patients or family members about the prognosis of PICH and when planning intervention trials. | Uncertain case of IVH without hydrocephalus |
| Kim et al., 2013^33^  Korea | Total (1,558)  S-ICH non-ESRD (1,456)  S-ICH ESRD (102) | Single center  Retrospective | We retrospectively studied consecutive patients with a diagnosis of S-ICH admitted to the Stroke Unit at our single hospital between January 2000 and December 2011. Patients aged greater  than 40 years were selected because of the likelihood of a secondary etiology in younger patients. In addition, we excluded  patients with an infratentorial hemorrhage, because small changes in hemorrhage size or location are believed to have greater  impacts on survival than supratentorial hemorrhages in these  patients. Patients with a hemorrhage secondary to head trauma, a ruptured  cerebral aneurysm, an arteriovenous malformation, a tumor,  bleeding diathesis, or a hemorrhagic infarction were excluded. | 30 days, 3 months, 6 months mortalities and Glasgow Outcome Scale | Hypertension, diabetes mellitus, hyperlipidemia, cerebral infarction, ischemic heart disease, atrial fibrillation | Conservative, surgery (craniotomy, craniectomy, sterotactic aspiration, ventriculostomy, combined) | Supratentorial only (basal ganglia, thalamus, lobar) | Age, consciousness during admission, pupillary abnormalities, limb weakness, GCS score, hematoma location, midline shift, blood parameter  Time to surgery, laboratory findings, | This retrospective study showed worse outcome after S-ICH in patients with ESRD than those without ESRD; 30-day mortality was  four times higher and the functional recovery rate was signifcantly lower in S-ICH patients with ESRD than in S-ICH patients without ESRD | Not reporting comparision of mortality or functional outcome in ICH vs IVH vs hydrocephalus |
| Kim et al., 2013^34^  Korea | Total (1,262)  ICH (919) ICH + IVH (343) | Multicenter (33 hospital)  Prospective | Between October 2002 and March 2004, 33 hospitals across the country participated. Exclusion criteria: CT scans more than 48 hours after symptom onset, poor quality CT scans, primary intraventricular hemorrhage without parenchymal hematoma. | Glasgow coma scale score  30 day mortality  Deceased at December 2006 | Hypertension diabetes | Surgery: hematoma evacuation, aspiration, EVD, others  Conservative | Supratentorial: lobar, deep structure  Infratentorial hemorrhage: brainstem, cerebellum | Factor related to extension of IVH: laboratory findings, location of hematoma, volume of hematoma, white matter lesions | We documented that the severity of white matter lesions is related to the occurrence and amount of intraventricular extension of hemorrhage in spontaneous intracerebral hemorrhage cases | Uncertain cause of ICH, might involve traumatic cause and structural anomalies |
| Koivunen et al., 2014^35^  Finland | Total (325) | Single center  Retrospective | All the patients treated in the Helsinki University Central Hospital  from January 1, 2000, to March 31, 2010, having an International  Classifcation of Diseases, 10th Revision, diagnosis code of Q28.1,  Q28.3, I60.8, I61, I67.3, I67.4, I67.5, I67.6, I67.7, I67.8, I67.9, I68,  or I78 were screened for nontraumatic frst-ever ICH.  Hypertensive microangiopathy was considered the cause of ICH if hypertension was present, and location of  hematoma was deep or infratentorial.  Other causes included cerebral venous thrombosis, vasculitis, illicit drug use, eclampsia, liver disease, and syndrome of  hemolysis, elevated liver enzymes, and low platelet count | 3-months mortality | Not stated | Evacuation of hematoma, ventriculostomy, decmpressive craniectomy | Supratentorial (lobar, deep), infratentorial (cerebellar, pontine, cerebellar and pontine), mixed | Sex, age, NIHSS score, location of hemorrhage, volume of hemorrhage, IVE, hydrocephalus, multiple hemorrhage, herniation, procedure | The predictors of short-term case fatality are alike in young and elderly patients with ICH. However,  initial hematoma evacuation was associated with lower 3-month case fatality in our young patients with ICH. | Included structural cause of hemorrhage |
| Koivunen et al., 2015^36^ | Total (131) | Single center  Retrospective | Patients included had their non-traumatic first-ever  ICH between the ages of 16 and 49 treated in HUCH  between 1 January 2000 and 31 March 2010  30-days survivors since initial diagnosis with ICH | Long-term mortality until 2013 (1 to 5 years)  mRS functional outcome  (Favorable 0-1, unfavorable 2-5) | Hypertension, diabetes mellitus, cardiac disease | Hematoma evacuation | Not specified | Gender, age, comorbidities, NIHSS score on arrival, hematoma volume, location, hydrocphelus, IVH | Of every 10 survivors of acute phase ICH at a young age, one  died within 10 years after onset, male sex and diabetes being associated with  increased mortality. Half the survivors did not achieve a favorable functional  outcome, which was predicted by increasing age, initial stroke severity and  intraventricular hemorrhage. | Uncertain cause of long-term mortality  Classificatin for mRS outcome didn’t match our criteria |
| Lai et al., 2015^37^  Taiwan | Total (296)  Ganglion (145), Lobar (74), multiple (8), posterior fossa (60), IVH (9) | Single Center  Retrospective | We retrospectively reviewed patients aged 15–45 years  at the Chung Gung Memorial Hospital between January 2000 and December 2001 who had ICH (codes  431, International Classification of Diseases, 9th  Revision). Patients with hemorrhagic  infarct or traumatic hemorrhage were excluded. | Mortality, clinical features (headache, dizziness, vomiting, disturbed consciousness, limb weakness, sensory dysfunction, speech disturbance, incontinences, seizures) | HTN, vascular anomaly, lcoagulopathy, tumor, smoking, alcohol, cryptogenic | Not stated | Ganglion, lobar, multiple, posterior fossa, IVh | Comorbidities, age, sites, cause | The most common risk factor  for mortality was hypertension (HTN) (48.7%). Bleeding was most common in the  ganglion (49.0%). Multiple hemorrhages (83.3%) caused the highest mortality, with  the most common cause of mortality being HTN (46.6%). Coagulopathy (62.5%)  caused the highest mortality based on etiologic classification. Recurrent HTN-induced  hemorrhage rate was 3.6%. In Taiwanese adults £45 years of age, ICHs mainly involve the ganglion and result from HTN. | Included tumor, vascular anomalies cause of ICH  Uncertain whether IVH is primary or secondary to ICH |
| Lee et al., 2017^38^  Korea | Total (112)  Favorable (72)  Unfavorable (40) | Single center  Retrospective | We reviewed all patients with ICH who were admitted at our  institution between January 2004 and December 2014. We analyzed patients with spontaneous IVH without evidence of ICH  or SAH. Exclusion criteria were: neonates (less than 1 month  after birth), death within 24 hours of presentation, and life expectancy less than 6 months owing to another medical condition. | Favorable outcome (GOS > 4), unfavorable outcome (GOS <4) | Hypertension, diabetes, use of anticoagulant, use of antiplatelet | EVD | Not stated | Initial GCS, simplified acute physiolocy score, gender, mechanical ventilation, laboratory findings, Graeb score, diameter of ventricle, hydrocephalus | In this study we reveal independent predictors of poor outcome in primary intraventricular hemorrhage patients, and show that nearly half of the patients in our study had predisposing vascular abnormalities. Routine  angiography is recommended in the evaluation of PIVH to identify potentially treatable etiologies, which may  enhance long-term prognosis. | Primary IVH |
| Liliang et al., 2000^39^  Taiwan | Total (36) | Single center  Retrospective | We retrospectively reviewed medical records of all patients who had  a diagnosis of spontaneous ICH at Koahsiung Chang Gang Memorial  Hospital during the period January 1995 to December 1999.  We excluded hemorrhages that resulted from cerebral  trauma, rupture of arteriovenous malformation, ruptured aneurysm. Patients with spontaneous subarachnoid hemorrhage (known previously or  later documented angiographically), tumor bleeding, or bleeding  diathesis. Patients with neurological deficits from previous head  injury, cerebral infarction, or intracranial hemorrhage were also  excluded | 6- months functional outcome (Glasgow Outcome Scale) | Not stated | EVD, medical | Not specified | Gender, initial GCS, volume of hematoma, hydrocephalus, rupture into internal capsule, IVH score, hemorrhagic dilatation of 4^th^ ventricle | Hydrocephalus is the most important predictor of poor outcome. External ventricular drainage response for  hydrocephalus was good in the present study, whereas an early decision should be made regarding preoperative  neurological condition |  |
| Lim et al., 2020^40^  Singapore | Total () | Single center  Retrospective | We conducted a retrospective review of 297 consecutive  patients with spontaneous ICH admitted to the National  University Hospital, Singapore between December 2014  and May 2016. | 30-days mortality  90-days functional outcome (mRS) | ADL dependent, dementia, previous ICH, hypertension, diabetes mellitus, dyslipidemia, dialysis, smoker, anticoagulation | Surgical | Infratentorial, supratentorial | Age, gender, race, comorbidities, median GCS, pulse pressure, infratentorial bleed, IVH, surgical evacuation | Current prognostic scores  performed acceptable-to-good in our patient cohort. Future studies may be useful  to investigate the utility of these scores in clinical decision-making. | Unspecified cause of ICH |
| Liotta et al., 2013^41^ | Total (246) | Single center  Retrospective | Patients presenting to our institution with ICH between December 2006 and July 2012 were  enrolled in a prospective observational cohort registry. | Re-admission to hospital | Alcohol abuse, atrial fibrillation, coronary artery disease, diabetes mellitus, historical hypertension, history of TIA or stroke, anticoagulant, antiplatelet | Craniotomy, extraventricular drain | Lobar, thalamus, cerebellar, lentiform nucleus, caudate, brainstem | Initial GCS, Admission NIHSS, Initial ICH score, APACHE acute physiology score, APACHe IV predicted ICU LOS, initial ICH volume, ICH location, IVH, Admission INR, ventilator free days, endoscopic gastronomy during admission, tracheostomy during admission | Severity of illness and hospital complications were not associated with 30-day  readmission. The most common indication for readmission was infection after discharge, and  readmission was associated with worse functional outcomes at three months. Preventing  readmission after ICH may depend primarily on optimizing care after discharge and improve  functional outcomes at three months. | Didn’t report outcome of interest |
| Louis et al., 2000^42^  USA | Total (96)  Cerebellar hemorrhage present (45)  Cerebellar hemorrhage absent (51) | Single center  Retrospective | Spontaneous cerebellar hemorrhage was  confirmed by CT scan (n = 93) or autopsy (n = 1). The medical  records were retrospectively reviewed. We reviewed the medical  records of all Mayo Clinic Patients with intracerebral  hemorrhage from 1975-1993 (n = 2,783). We excluded 102  patients with cerebellar hematomas due to underlying causes. | In hospital mortality, anisocoria, pinpoint pupils, abnormal corea reflex, abnormal coulocephalic reflex, skew deviation, facial palsy, episodic bradycardia, systolic blood pressure >200 | Hypertension, anticoagulation, syncope at onset | Not stated | Cerebellar | Comorbidities, GCS, motor response, abnormal CR, abnormal OCR, hydrocephalus, IVH | A tree-based analysis model  using binary recursive partitioning showed that cornea reflex, hydrocephalus, doll’s eyes, age, and size were the most important  discriminating factors. Absent corneal reflexes on admission highly predicts poor outcome (86 percent, confidence limits 67-96 percent).  When a cornea reflex is present, acute hydrocephalus predicts poor outcome but only when doll’s eyes are additionally absent | Not reporting outcome of interest |
| Mahta et al., 2016^43^ | Total (164)  ICH (72)  ICH + IVH (31)  ICH + IVH + HC (61) | Single center  Retrospective | This was a retrospective cohort study of patients with ICH who  were admitted to the Neuroscience Intensive Care Unit at a tertiary  referral center from 2008 to 2010.  We included patients with primarily  spontaneous intra-parenchymal hematoma without any obvious  radiographic evidence of underlying vascular malformation or neoplastic lesion.  These patients were classified as ‘‘hypertensive”  bleed or ICH with undetermined etiology in the medical records.  The exclusion criteria consisted of primary epidural or subdural  hematomas, aneurysmal subarachnoid hemorrhages, ischemic  infarcts with hemorrhagic conversions, any underlying neoplastic  lesions or vascular malformations, because pathophysiology is different in each category. | ICH score, hematoma volume, mRS, poor outcome (mRS 3-6)  Mortality  GCS | Not stated | Antiplatelet, EVD placement | Lobar, thalamus, putamen, pons, cerebellum | Age, sex, presenc of IVH, hydrocephalus, GCS, hematoma volume, location, treatment | Only hydrocephalus (p = 0.002) and hematoma volume (p = 0.006) were significantly associated with mortality or  poor functional outcome (mRS of 3 to 6). In contrast, the presence of intraventricular hematoma was  not independently associated with poor functional outcome. The presence of intraventricular extension  of ICH in the absence of hydrocephalus may not increase mortality or disability. | Follow up ranging from 3 months to 7 years |
| Mansouri et al., 2013^44^  Italia | Total (120)  Survivors (90)  Non-survivor (30) | Single center  Prospective | One hundred and twenty consecutive patients with nontraumatic and non-aneurysmatic ICH who presented to  Emergency Department of Imam Hossein Medical Center  between January and July 2012.  Patients with  secondary etiologies of cerebral hemorrhage (trauma,  ruptured aneurysm, structural lesions and tumor); and those  who were referred from outlying centers were excluded. | 30-day mortality  3-month mortality | Hypertension, DM  Hyperlipidemia  Smoker | Antiplatelet | Not stated | Age, sex, pas medical history, temperature during admission, pulse rate, systolic arterial pressure, respiratory rate, GCS, NIHSS, ICH, laboratory data  IVH, midline shift, hydrocephalus | Moreover, the parameters on the initial computed tomography scan significantly increased 30-day fatality rate and  was correlated with increase in the discharge mRS score of  survivors. |  |
| Marti-Fabregas et al., 1999^45^ | Total (13) | Single center  Retrospective | We diagnosed PIVH when computed tomography  (CT) revealed hyperdensity restricted to the ventricular system.  We excluded patients with parenchymatous hemorrhages, even if  the hemorrhage was small or very close to the ventricular system,  and also patients with a history of head trauma, and new-born infants. | 9 years mortality | Hypertension, vascular malformation, tumour | Not stated | Ventricle | Age, gender, graeb index, ventricular ratio | We defined PIVH as hemorrhage detected by CT in the ventricular system only. The major symptoms included headache (n = 13), decreased  level of consciousness (n = 9), and  nausea/vomiting (n = 7). The cause  was unknown in five patients; and was associated with arterial hypertension in five, vascular malformations in two, and tumor in one, although arteriography was performed  in only five patients. Outcomes were  death in three, asymptomatic in six,  mild disability in three, and moderate disability in one. Prognosis was  not related to clinical or CT data.  Clinical features can suggest the diagnosis of PIVH, but cerebral CT is  required for confirmation. | Primary IVH |
| Mase et al., 1995^46^ | Total (138)  Alive (100)  Dead (38) | Single center  Retrospective | The medical records of all the patients with a diagnosis of PICh admitted to the Department of Neurology, University of Trieste over the years 1989 to 1993 were carefully reviewed. The inclusion criteria were: 1) admission < 24 h after stroke onset; 2) CT demonstration of PICH; 3) absence of a definite cause of hemorrhage other than arterial hypertension (i.e. head injury, anticoagulant therapy, aneurysm, or arteriovenous malformation, hemorrhagic infarction, tumor; 4) supratentorial site. | 30-day mortality | Hypertension, left cardiac hypertrophy, diabetes mellitus, coronary heart disease, chronic obstructive broncopathy, cigarette smoking, alcohol consumption  Dyslipidemia | Not stated | Deep, lobar | Age, gender, comorbidities, blood pressure, blood sugar, GCS score, limb paresis, pupillary abnormalities, gaze deviation, hemorrhage volume, IVS of blood, midline shift | Using univariate analysis we found eight significant of 30-day mortality: intraventricular spread (IVS) of blood, volume of the hemorrhage, Glasgow Coma Scale (GCS) score, midline shift, hyperglycemia, pupillary abnormalities, limb paresis, and gaze deviation. | Uncertain case of IVH without hydrocephalus |
| Masehlaty et al., 2012^47^ | Total (556)  Surgery (556)  Conservative (261) | Single center retrospective | In the current study, we excluded patients  with hemorrhage located infratentorially from  the analysis. Due to an essential impact of the  underlying cause of the hemorrhage on performed therapy, patients with immediately  diagnosed vascular malformations as the  source of the hemorrhage and hemorrhage  related to cerebral ischemic stroke were  excluded from the study as well.  ICH was divided into five categories: uni-lobar, bi-lobar,  multi-lobar, ventricular hemorrhage and hemorrhage in the basal ganglia. | Glasgow Outcome scale | Hypertension, diabetes, Coronary heart disease, cerebrovascular disease | Surgery vs conservative | Uni lobar bi lobar, multililobar, basal gangliang, basal + lobar | GCS, Age, Comorbidities, Etiology, complications (re-bleeding, HCP, ischemia) | The results of our study show that ICH remains  a multifarious disease and challenges neurosurgeons repeatedly. Selection of the treatment modality and prediction for neurofunctional outcome underlies various parameters.  Treatment recommendations of ICH remain an  unsolved issue. The consideration of the GCS  grade at admission is the most important predictive factor. Old age is not an absolute contraindication for surgery, but cumulative  multi-morbidity, especially cerebrovascular  and cardiovascular diseases and oral anticoagulant therapy should be regarded critically in  view of surgical treatment. | Uncertain cause of IVH (primary or secondary to ICH)  Included tumor cases |
| Mayfrank et al., 2001^48^ | Total (219)  Dead (30)  Alive (189) | Single center  Retrospective | All patients with their first aneurysmal SAH admitted to our Department of Neurosurgery between January 1990 and February  1996 were enrolled in the study if they fulfilled the following criteria: admission within 72 h from onset of symptoms, availability  of a preoperative CT scan performed within 72 h from SAH and  prior to ventricular or spinal cerebrospinal fluid drainage, and aneurysmal origin of SAH proven by four-vessel arterial digital subtraction angiography. Patients were excluded if they had known  residual neurological or neuropsychological symptoms secondary  to previous ischemic or hemorrhagic stroke, brain injury, or intracranial neoplasms. | Mortality at 14 days after SAH, 6-month unsatisfactory outcome (Glasgow outcome scale) | Not stated | Not state | Subarachnoid | Gender, Hunt and Hess score, Fisher score, SAh score, IVH score, ICH volume, bicaudate index, age | According to  logistic regression analyses, the severity of IVH was an  independent predictor of mortality and functional outcome. The clinical outcome after aneurysm rupture is at  least in part determined by the severity of IVH. Knowledge of the effect of IVH may help guide physicians in  the care of patients with aneurysmal bleeding. | Rupture intracranial aneurysm  Subarachnoid hemorrhage |
| Mustanoja et al., 2015^49^  Finland | Total (967)  IVH (398)  Non-IVH (569) | Single center  Retrospective | All consecutive ICH patients from January  2005 to March 2010 are included, with data retrospectively  retrieved from medical records, including province-wide electronic patient records and imaging databases. | 90-days mortality | Not stated | EVD, MV, ICU | Deep, lobar, infratentorial, hydrocephalus, herniation, midline shift | Age, gender, NIHSS, ICH volume, IVH, infratentorial location, etiology | The presence of intraventricular hemorrhage was  independently associated with increased mortality, and all the  intraventricular hemorrhage scores were strong predictors of  three-month mortality. | Uncertain case of IVH without hydrocephalus |
| Nishikawa et al., 2009^50^ | Total (100)  IVH (35)  No IVH (65) | Single center retrospective | The medical records of 129 patients with spontaneous supratentorial ICH or pure IVH treated in our hospital between January  2005 and December 2006 were retrospectively reviewed. Those  with other underlying critical or previous symptomatic cerebral  diseases (13 patients) were excluded. | Karnofsky Performance Status (KPS), hospital stays | Not stated | Surgical ICH removal, EVD | Thalamus, putamen, lobe, pure IVH | Age, gender, GCS, ICH location, ICH volume, Graeb score, hydrocephalus, surgical, EVD | IVH severity influenced the occurrence of acute hydrocephalus and initial level of consciousness, which was significantly associated with prognosis. Our results suggest that priority treatment of  the IVH should be given to those ICH patients with IVH admitted with a Graeb score of 6 or more. | Mixed with pure IVH cases  The etiologies for ICH not stated |
| Oh et al., 2008^51^ | Total (26) | Single center  Retrospective |  |  |  |  |  |  | A low initial GCS score, a high Graeb's  score, the absence of a pupil reflex, a high VCR and the presence of obstructive hydrocephalus are associated with a poor outcome in patients  with primary intraventricular hemorrhage. | Written in Korean |
| Pai et al., 2020^52^ | Total | Single center  Retrospective | We conducted a retrospective review of patients enrolled  in our prospectively maintained intracerebral hemorrhage register from February 2015 to February 2019. Exclusion criteria were age <18 years patients with associated intraparenchymal, subarachnoid, or subdural  hemorrhage, primary or secondary brain tumors with  hemorrhage, hemorrhagic transformation of an ischemic  stroke on initial computed tomography (CT) scan. | Favorable outcome (mRS >3)  Poor outcome (mRS <3) | Hypertension, antiplatelet, anticoagulants | Not stated | Not stated | Age, sex, GCS initial, comorbidities, Graeb score, acute hydrocephalus, etiology | PIVH is an uncommon entity but carries a better long-term prognosis  than SICH angiography helps in diagnosing surgically remediable underlying vascular  anomalies and is indicated in all cases of PIVH. | Primary IVH |
| Parry-Jones et al., 2013^53^  UK | Total (1175) | Single center retrospective | National Health Service (NHS) Research Ethics Committee approval was obtained for our study. We identifed 1364 patients referred between January 1, 2008, and October 17, 2010, whose  diagnosis had been recorded as ICH.  Cases  were included in the analysis if the frst CT brain scan after onset  could be obtained for review. If there was a clear history of a major head injury before presentation, patients were assumed to have  sustained a traumatic ICH and were excluded. Cases in which the  diagnosis was of hemorrhage into other intracranial compartments  without ICH, no hemorrhage at all, or hemorrhagic transformation  of an infarct were excluded. | 30-Day mortality | Hypertension, diabetes, hyeprcholesterolemia | Anticoagulant, antiplatelet, surgery | Deep, lobar, supratentorial, infratentorial | Gender, age, medical history, medication, GCS, location, ICH volume, IVH, surgery | Although existing grading scores are highly predictive of 30-day mortality, GCS alone was as predictive in  our cohort, but age was not. | May include tumor and vascular malformation cases  No information whether IVH was caused by ICH or pure IVH |
| Phan et al., 2000^54^  USA | Total (52) | Single center  Retrospective | We reviewed the medical records and nonenhanced CT. Deep cerebral hemorrhage was divided into 2  groups: putaminal hemorrhage (lateral group) and thalamic  and caudate hemorrhage (medial group). | 30-day mortality | Not stated | Not stated | Thalami and caudate, putaminal | GCS<8, hydrocephalus | Obstructive hydrocephalus on admission in a comatose patient with a putaminal hemorrhage predicts 30-day  mortality | The causes of hemorrhage were not stated |
| Pong et al., 2012^55^  China | Total (72)  Patients died within 30 days (12)  Patients survived the first 30 days (60) | Single center, retrospective | From May 1996 to February 2010, 691 consecutive patients  with first episode of spontaneous ICH admitted to our  neurosurgical unit were recruited. Patients were excluded  if they had ICH secondary to arteriovenous malformation,  aneurysm, cavernoma, other vascular malformations, tumor,  trauma, hemorrhagic transformation of cerebral infarctions,  or if they were aged <18 years. | 30-days mortality  6-month functional outcome (mRS score) | Hypertension, diabetes, atrial fibrillation, congestive heart failure | Hematoma evacuation, EVD, conservative | Cerebellar only | Gender, smoking history, alcohol history, comorbidities, pre-morbied modified Rankin scale <2, GCS, IVH, hydrocephalus, hematoma evacuation | Of 440 patients with  primary ICH, 72 (16.4%) had primary cerebellar hemorrhage. The mean age was 67.5±12.3 years and patients were  predominantly male (54%). The 30-day mortality was  16.7% with Glasgow coma scale ≤8 as the only predictor.  At 6 months, 56.7% of patients who survived the first  30 days had a persistently poor functional status with modified Rankin scale score >2. After a mean follow-up of 4.7 years, 3.3% of patients had recurrent ICH, a recurrence  rate of 7.3 per 1,000 patient-years. Ischemic stroke occurred  in 12% of patients, an incidence of 25.5 per 1,000 patientyears. This study provided data on the long-term outcome of  post-cerebellar hemorrhage in Chinese patients | Uncertain case of IVH without hydrocephalus |
| Portenoy et al., 1987^56^  USA | Total (52) | Single center  Retrospective | The hospital charts and CT scans of all patients admitted to the Bronx Municipal Hospital Center of the Albert Einstein College of Medicine with the diagnosis of non-traumatic, non-neoplastic, supratentorial ICH during the years 1981-1983  ICH location, size, and the presence of midline shift, hydrocephalus, or extension of blood into the ventricles or subarachnoid space were recorded. | Good outcome, poor outcome | Not stated | Not stated | Supratentorial | GCS score, alertness, coma, ataxic respirations, abnormal pupils, acute hypertensions, hemorrhage size, intraventricular spread | Multivariate analysis using the technique of logistic regression identified three variables, the Glasgow Coma Scale score, hemorrhage size, and intraventricular extension of blood, which were most predictive outcome. | Included cases of aneurysm or vascular malformations and subarachnoid hemorrhage  Standard for functional outcome wasn’t stated  No defined period of assessment |
| Qureshi et al., 1995^57^  USA | Total (182) | Single center, retrospective | All black Americans admitted to Grady Memorial Hospital in Atlanta, Ga, with the diagnosis of SICH confirmed by CT scan between January 1990 and July 1993 were evaluated for inclusion. We excluded patients in whom intracerebral hemorrhage was known or suspected to be secondary to infection, brain tumor, vasculitis, trauma, arteriovenous malformation, rupture of berry aneurysm, or hemorrhagic transformation of prior cerebral infarction. | Early mortality (24h) | Not stated | Not stated | Supratentorial  Infratentorial | Age, sex, blood pressure, site of hemorrhage, presence of IVH, ICH volume, initial GCS score | Hemorrhage volume and ventricular extension are the best predictors of early deterioration and mortality in black Americans with SICH | The mortality end-point was too short |
| Razzaq et al., 1998^58^  Pakistan | Total () | Single center, | The study was conducted at the Aga Khan University Hospital, a large tertiary care hospital in Karachi, Pakistan. The medical records of all patients  admitted with a diagnosis of ICH over a 2-year period (January 1990–December 1991) were obtained  from the medical records department of the hospital. Cases were defined and included in the study on  the basis of admission diagnosis of spontaneous  ICH with no previous history of stroke. Cases with  either subarachnoid hemorrhage or ICH secondary  to trauma were not included. | 30-days mortality | Hypertension | Not stated | Putamen/thalamic, lobar, posterior fossa, other | Age, gender, hypertension, pulse pressure, GCS score, limb paresis, pupillary dilatation, gaze deviation, speech deficit, motor power, UMN facial nerve palsy, size, site, IVH, midline shift, edema | The 30-day mortality rate and prognostic predictors for  spontaneous intracerebral hemorrhage were found to be  similar to those reported in the Western hemisphere.  However, the correlation of incremental increase in pulse pressure with deteriorating prognosis was a new and  significant finding. | The only excluded cases were trauma and subarachnoid hemorrhage  Could include cases of vascular malformation and tumor |
| Rodrigues et al., 2020^59^ | Total (402)  Alive (178)  Dead (224) | Single center  Prospective | We prospectively identified all incident cases of spontaneous ICH  in adults (aged ≥16 years) between June 1, 2010 and May  31, 2013 inclusive using multiple overlapping sources of  case ascertainment.5 For this study, we excluded patients  with ICH secondary to an underlying macrovascular or  structural cause other than SVDs, patients with a previous  symptomatic ICH, and patients without a diagnostic noncontrast brain CT.  Exclusion: secondary ICH due to malignancy, arterial aneurysm, arteriovenous malformation, carvenous malformation, intracranial venous thrombosis, dural arteriovenous fistula, abscess | 1 year mortality | Hypertension, ischemic stroke, transient ischemic attack, dementia, diabetes, atrial fibrillation, myocardial infarction, hyperlipidemia | Not stated | Lobar, deep infratentorial | ICH location, ICH volume, IVH, SAH, subdural hemorrhage, fingerlike projections, number of lacunes, Anterior WMLs | In conclusion, SVD biomarkers on the diagnostic  brain CT are associated with 1-year death and dependence  after ICH, independent of age, features of the acute ICH,  and other known prognostic factors. Future studies should  assess whether the associations of CT SVD biomarkers  with outcome after ICH can be reproduced in larger  cohorts and participants of different ethnicities. | 1 year mortality  Cases of traumatic brain injury |
| Roeder et al., 2019^60^  Germany | Total (1112)  No IVH (651)  IVH (461)  IVH w/o EVD (191)  IVH EVD (270) | Single center  Retrospective | All consecutive patients with spontaneous ICH admitted in  the Department of Neurology, University Hospital Erlangen,  Germany, were included in our prospective institutional registry  (UKER-ICH; NCT03183167) over a 10-year period (2006–2015).  Patients with secondary ICH (i.e., hemorrhage due to tumor,  trauma, ischemic stroke, arteriovenous malformations, ruptured  aneurysm or secondary to thrombolysis) were excluded as described previously | 3-months mortality  3-months functional outcome | Cardiac comorbidity, arterial hypertension, diabetes mellitus, hyperchloesterolemia, history of stroke, antiplatelet therapy, OAC | EVD vs no EVD | Deep, lobar, cerebellar, brainstem | No confounding factors analyzed for mortality | Small  amounts of intraventricular blood (Graeb score ≤2) not leading to obstructive hydrocephalus are not associated with unfavorable outcome or death after ICH. Thus, IVH per se should  not be considered a binary variable in outcome prediction  for ICH patients. |  |
| Shimoda et al., 2017^61^  Japan | Total 241  Good outcome: 67  Poor: 174 | Single center, retrospective | A total of 257 patients with spontaneous ICH were admitted to  the Neurosurgery Department at South Miyagi Medical Center between April 2013 and December 2015.  Patients who underwent  nonenhanced CT scans within 12 h of symptom onset were included in this study. Patients with ICH caused by brain tumor,  vascular anomalies (arteriovenous malformation, aneurysm, and  cavernous angioma), and trauma were excluded from this study. | Functional outcome (mRS score) | Hypertension, diabetes mellitus, hyperlipidemia, arterial fibrillation, prior stroke, hemodialysis, antiplatelets, anticoagulants | Not stated | Deep, brainstem, lobar, cerebellum | Gender, clinical history, medications, blood pressure, laboratory data, location, hemorrhage size, IVH | The presence of a satellite sign in the  initial CT scan is associated with a significantly worse functional outcome in ICH patients. | End-point of outcome was not determined |
| Sloan et al., 1998^62^  Multicenter (15 countries) | Total: 268  Died: 160  Survived: 108 | Multicenter  Retrospective | Patients presenting to a participating hospital less than 6 hours after the onset of symptoms, with chest pain lasting at least 20 minutes and accompanied by electrocardiographic signs of ≥ 0.1 mV of ST-segment elevation in two or more limb leads or ≥ 0.2 mV in two or more contiguous precordial leads were eligible for enrollment. The criteria for exclusion were previous stroke, active bleeding, previous treatment with streptokinase or anistreplase, recent trauma or major surgery, previous participation in the trial, or noncompressible vascular punctures. | 30-days mortality | Hypertension, diabetes, smoker, hypercholesterolemia, previous infarction, previous angina, cerebrovascular disease, | Thrombolytic: Sterptokinase-SQ heparin, streptokinase-IV heparin, alteplase-IV heparin, combined | Anterior, inferior, other | Comorbidities, gender, location, killip class, thrombolytic strategy, time to even, polatelet count, GCS, blood pressure, type of hemorrhage, total volume, blood fluid present, mass effect, herniation, hydrocephalus | This model provides excellent discrimination between patients who are likely to live and those who are  likely to die after thrombolytic-related intracranial hemorrhage; this may aid in making decisions about the appropriate  level of care for such patients. | Also include infarction and trauma cases  Also include subarachnoid hemorrhage |
| Staykov et al., 2011^63^  Germany | Total: 50 | Single center, retrospective | Patients with spontaneous hypertensive ganglionic  ICH <40 ml, severe ventricular involvement and  acute obstructive hydrocephalus due to obstruction  of the third and fourth ventricles were included.  Exclusion criteria were oral anticoagulant therapy,  international normalised ratio on admission >1.4,  coagulopathy, ICH due to trauma, tumours or  vascular malformation, infratentorial ICH, hydrocephalus due to mass effect of the parenchymal ICH with blockage of the foramen of Monro, enrolment >48 h  after symptom onset and age <18 years. | mRS value | Not stated | IV fibrinolysis | Putamen, caudate, thalamus | GCS, ICH volume, age, NIHSS on admission, IVH vol 3^rd^ ventricle, IVH vol 4^th^ ventricle, Graeb score | Despite rapid clot removal, initial IVH  volume in the third ventricle was a strong and  independent negative predictor. This is possibly  explained by irreversible damage of brainstem structures  by the initial mass effect of IVH. | Number of patients with poor vs good outcome was not written |
| Stein et al., 2010^64^  Germany | Total: 104  Dead: 31  Survive: 73 | Single center, retrospective | This study focused on patients with deep-seated supratentorial hemorrhage with ventricular extension. Hospital charts of patients who were  admitted with SICH to the Department of Neurosurgery of the University  Hospital Giessen between January 1995 and December 2002 were retrospectively reviewed. Inclusion criteria were intracerebral hemorrhage (ICH)  with ventricular extension, with an age range of 18 to 90 years. Only patients  with at least 1 external ventricular CSF drain were included.  Exclusion  criteria were ventricular hemorrhage caused by cerebral trauma, rupture of an  aneurysmal or vascular malformation, spontaneous subarachnoid hemorrhage, primary IVH, infratentorial origin of the hemorrhage, SICH with  extension into the brainstem, tumor hemorrhage, or ischemic stroke. Lobar  hemorrhage was excluded because it may be associated with better outcome  and may benefit from early surgery compared with deep-seated lesions. | 30-days mortality  6 month functional outcome | Not stated | Not stated | Putamen, thalamus, caudate, total basal ganglia | Age, gender, body temperature, blood pressure, serum glucose, localization, site of hemorrhage, GCS score, hematoma volume, Graeb score, hydrocephalus | Severe hydrocephalus is an independent predictor of 30-day mortality in  SICH with ventricular extension. The IVH score is a simple and reliable tool for predicting  30-day mortality |  |
| Stein et al., 2011^65^  Germany | Total: 161` | Single center, retrospective | Inclusion criteria were the confrmation of SICH and secondary IVH by computerized tomography (CT) and patient  age of 18–90 years.  All included patients received at least one external ventricular drain (EVD) to relieve obstructive hydrocephalus.  All EVDs were placed in the frst 24 h after ictus at our  department after the initial CT scan had been reviewed.  Patients with traumatic hemorrhage, ischemic stroke,  brain tumor, subarachnoid hemorrhage due to aneurysm or  malformation, infratentorial origin of hemorrhage, SICH  spreading into the brainstem, strict lobar hematoma, and  therapeutic anticoagulation were excluded. Decompressive  craniotomy and evacuation of the hematoma were also  defned as exclusion criteria | 30 days mortality  6- months functional outcome | Not stated | Not stated | Putamen, thalamus, caudate, total basal ganglia | Age, GCS, hematoma side, localization, volume of hematoma, HC, Graeb score | The scores that include the quantification of IVH or the  grading of hydrocephalus show good accuracy in the prediction of 30-day mortality and functional outcome at 6 months  in SICH with secondary IVH. | No description of number of patients |
| Takahashi et al., 2006^66^  Japan | Total: 347  Dead: 70  Survive: 277 | Single center, retrospective | We excluded  traumatic or subarachnoid haemorrhages.  Information from August 1998 to December 2001  was extracted from a computerized database  (SHIMANE: Integrated Intelligent Management  System). | In hospital mortality | Hypertension, diabetes, ischamic heart disease, heart failure, stroke | Operation vs non operation | Lobar, central, cerebellar, brainstem | Alert/disoriented at admission, IVH, hematoma site, operation | ICH patients can easily be stratified  for mortality risk, based on three predictors  available on admission. This simple decision tree  model provides clinicians with a reliable and  practical tool | May also include tumor cases  Not defined period of in hospital mortality |
| Togha-Bakhtavar et al., 2004^67^  Iran | Total: 122  Deceased: 59  Survive: 63 | Single center, retrospective | For each patient,  demographic data (age and sex), vascular risk factors,  MAPs and GCSs upon arrival and neuroimaging findings  were recorded. Anamnestic findings consisted of the history of hypertension, diabetes mellitus, smoking (>20 cigarettes/day), and previous cerebral infarction. | In hospital mortality | Hypertension, diabetes mellitus, smoking, ischemic heart disease | Not stated | Not stated | Gender, age, comorbidities, GCS score, mean arterial pressure, midline shift, IVH, hydrocephalus, hematoma volume, length of hospital stay | Higher rate of mortality were observed during the first two weeks of hospitalization  following ICH. Neuroimaging features along with GCS score can help the clinicians in developing  their prognosis. | Etiologies not stated |
| Trifan et al., 2019^68^  Spain | Total: 210 |  | We included patients aged older than 18 with spontaneous sICH,  which was defined as parenchymal hemorrhage that was not  related to tumor, vascular malformations, or trauma. Exclusion  criteria were hemorrhagic infarction, anticoagulation associated  ICH, and intraventricular treatment with recombinant activated  tissue plasminogen activator | mRS score at hospital discharge | Diabetes, hypertension, previous ICH, ischemic stroke | VP shunt, clot evacuation | Deep lobar | Age, gender, history of HTN, history of DM, antithrombotic use, GCS score, sICH volume, IVH, hydrocephalus, hematoma location, VP shunt, clot evacuation | Higher IVH severity (defined as Graeb score ≥5) is associated with worse  outcome at time of hospital discharge, while lower IVH severity (Graeb scores 1–4) has  similar outcomes to patients without IVH. IVH severity should be used in favor of IVH  presence for prognostication purposes. | Undefined end point period  No number of patients available, data was presented as risk ratio |
| Tshikwela et al., 2012^69^  South Africa | Total: 185  Died: 68  Survive 117 | Single center, retrospective | Patients with hemorrhage secondary to brain tumors,  trauma, hemorrhagic transformation of cerebral  infarction, anticoagulant therapy, aneurismal, or  vascular malformations were excluded. | 30-day mortality | Smoking | Not stated | Deep vs lobar | ICH volume, midline shift (mm), gender, moderate alcohol intake, ICH vol > 25, IVH, left hemisphere side, midline shift > 7mm, smoking, coma | In this study,  the Kinshasa ICH score seems to be an accurate method for distinguishing those ICH patients who need continuous  and special management. It needs to be validated among large African hypertensive populations with a high rate of  30-day in–hospital mortality | Included primary cause of primary IVH |
| Wang et al., 2014^70^  China | Total: 198  MIPD: 84  Hematoma evacuation: 114 | Single center, prospective | Patients were eligible for the study if they had a hypertensive spontaneous ICH in the basal ganglia with a  hematoma volume (HV) ≧30 mL, the hematoma evacuation could start within 24 hours of ictus (if the onset was  unobserved, it was considered to be at the last time the  patient was definitely normal), and the informed consent  for the operation could be obtained from patient’s relative  or guardian.  Patients with ICH located in the cerebral lobes, infratentorial or subarachnoid areas of the brain; ICH caused by  trauma, aneurysms, arteriovenous malformation; ICH  secondary to an ischemic infarction or coagulopathy; or  patients with previous neurological defects or without  definite hypertension were excluded. | 30-days mortality  1 year mortality  1 year good outcome | Diabetes | Minimally invasive puncture and drainage vs hematoma evacuation | Not stated | Age, gender, GCS, NIHSS, HV, IVH grade, complications (rebleed, pulmonary infection, upper gastrointestinal bleeding) | For patients with hypertensive spontaneous ICH (HV≧30 mL in basal ganglia), MIPD may be a more  effective treatment than DC, as assessed by a higher rate of functional independence at 1 year after onset as well as  reduced mortality in patients ≦60 years of age, NIHSS < 15 or HV≦60 mL | Uncertain case of IVH without hydrocephalus |
| Woo et al., 2012^71^  South Korea | Total: 263  Prophylatic anti-convulsant: 216  Non prophylatic anticonvulsant: 47 | Single center, retrospective | Patients with histories of seizure, stroke,  infection of the central nervous system, traumatic hemorrhage,  metabolic disorders, brain tumor, hemorrhage due to arteriovenous malformation, subarachnoid hemorrhage, cerebellar hemorrhage or brain stem hemorrhage were excluded | Odds ratio of factors associated with seizure after spontaneous ICH | Smoking, alcohol abuse | Craniotomy, prophylactic anticonvulsant | Lobar vs deep | GCS, smoking, alcohol, surgery, communicating HCP, obstructive HCP, postoverpative hemorrhage, rebleeding, ICH volume, cortical, IVH, lobar hemorrhage, prophylactic | Cortical involvement may be a factor for provoked seizures. Although the incidence of early seizures tended to decrease in patients  prescribed prophylactic anticonvulsants, no statistical difference was found. | No number of patients written |
| Wu et al., 2012^72^  Taiwan | Total: 70  Survive: 55  Mortlity: 15 | Single center, retrospective | Seventy patients diagnosed with acute spontaneous CH without  prior disability were admitted to our hospital between 2004  and 2011 within 48 h after the onset of symptoms. None of  the patients showed complications caused by other hemorrhagic lesions or hemorrhagic transformation of ischemic  stroke. | First-week mortality | Smoking, alcohol, hypertension, diabetes mellitus, coronary artery disease, dyslipidemia, coagulopathy | Surgical decompression, brainstem compression | Not stated | Gender, comorbidities, initial GCS, blood pressure, lesion site, vermis, max diameter, HC, IVH, brainstem compression, surgical decompression | This is the first study showing that GCS scores ≤8 on  arrival and brainstem compression are strong predictive  factors for first-week mortality in patients with CH. These  patients were about 32 and 14.5 times, respectively, more  likely to die within 7 days. | First week mortality, not outcome of interest |
| Yaghi et al., 2014^73^  USA | Total: 200  Expansion: 28  No expansion 172 | Single center, retrospective | Patients with ICH secondary to arteriovenous malformations, trauma, tumor,  aneurysm, infarction, cerebral venous thrombosis, moyamoya disease, infective endocarditis and patients who  underwent surgical evacuation were excluded. | Predictors of hematoma expansion in sICH | Smoking, alcohol, hypertension, stroke, diabetes, hypercholesterolemia, coronary artery disease, end stage renal disease | Not stated | Lobar, capsule-ganglionic | Comorbidities, use of cocaine, statin, aspirin, clopidrogel, Coumadin, lobar vs capsule-gangionic hemorrhage, IVH, HC, troponin leak, admission GCS, hematoma volume, blood pressure, blood glucose, intubation | Patient with prior Warfarin use and IVHs are at risk of hematoma  expansion. Aggressive measures to prevent hematoma growth are important in these patients. | No comparison between IVH and IVH +ICH  Not outcome of interest  Undefined period of in hospital mortality |
| Yang et al., 2009^74^  Taiwan | Total: 243  Immediate: 4  Early: 4  Late: 11  w/o seizure: 223 | Single center, retrospective | All patients underwent brain CT scanning. Based on its  location, a lobar hemorrhage abutting onto the cortex is  defned as cortical, and a deep subcortical hemorrhage is  defned as subcortical. Exclusion criteria included: 1) nonspontaneous ICH, such as traumatic ICH; 2) brainstem  and cerebellar hemorrhage, subarachnoid hemorrhage, arteriovenous vascular malformations, subdural hematoma,  hemorrhagic infarct, or inﬂammatory vascular disease;  3) spontaneous ICH caused by a primary or metastatic  brain tumor; 4) preexisting neurological conditions with  various neurological defcits (such as stroke, head trauma,  and hypoxic encephalopathy); and 5) a history of epilepsy  prior to ICH. | Immediate, early, late seizure | Not stated | Craniostomy w or w/o EVD, VP shunt/EVD, conservative | Basal ganglia, thalamus, corpus | Location of ICH, hydrocephalus, rebleeding of ICH, postop hemorrhage, complications after sICH: HC, pneumonia, rebleeding, postop ICH, hyponatremia, arrhythmia, postneurosurgical meningitis, diabetes insipidus | Higher mean ICH volumes at presentation were predictive of seizure, and the presence of late  seizures was predictive of developing epilepsy. Most seizures occurred within 2 years of spontaneous ICH over a  minimum of 3 years of follow-up. | Not outcome of interest |
| Ye et al., 2017^75^  China | Total: 67  Parenchymal hemorrhage: 48  w/o parenchymal hemorrhage: 19 | Single center, retrospective | We included the patients following the inclusion criteria:  diagnosed with AVMs by computed tomography angiography  (CTA), magnetic resonance angiography, or digital subtraction  angiography (DSA); initial computed tomography (CT) upon  admission showed the extension of hemorrhage into ventricles;  and with or without parenchymal hemorrhage. The patients with  IVH caused by other factors, such as tumors, trauma, and  moyamoya disease, were excluded. | Factors related to parenchymal hemorrhage and hydrocephalus  6 m.o. functional outcome | Hypertension, stroke | Surgery, gamma knife, embolization, EVD | Brain lobe, basal ganglia, infratentorial | Gender, intial GCS, SAH, Graeb score, aneurysm, parenchymal hemorrhage, hematoma volume, sup-SM score, MAP, hydrocephalus, brain ischemia, occurrence of hernia | IVH was common in ruptured AVMs and increased the poor outcomes in patients with the ruptured AVMs. The AVM-related IVH  patients had a high incidence of hydrocephalus, which was associated with brain ischemia and SAH. Patients with lower Glasgow  coma scale, lower sup-SM score, and smaller parenchymal hematoma had better long-term outcomes. | AVM cases |
| Yuan et al., 2016^76^  China | Total: 132  IVH: 71  Non-IVH: 61 | Single center, retrospective | The patients were included for  analysis based on the following criteria: (1) they were at  least 18 years old at ICH onset; (2) they met the World  Health Organization definition of stroke18 with subsequent computed tomography (CT) and/or magnetic  resonance (MR) to confirm the hemorrhage; and (3) they  were diagnosed with VSARICH, defined as aneurysm,  arteriovenous malformation, Moyamoya disease, or cavernous malformation at bleeding site verified by  radiological or pathological findings.17 Patients were excluded based on the following criteria: (1) they had a  modified Rankin Scale (mRS) score higher than 2 points  before ICH; (2) they underwent the first CT scan more  than 48 hours from symptom onset; (3) they had primary  IVH without parenchymal involvement; and (4) they had  any kind of surgical intervention (hematoma evacuation, decompressive craniectomy, or ventricular drainage)  before admission. | Three-month death, 3 month poor outcome  Factors associated with IVH | Hypertension, hyperlipidemia, diabetes mellitus, smoking, alcohol | Hematoma evacuation/decompression,  EVD | Lobar, supratentorial deep, brainstem, cerebellar, multiple | Comorbidities, initial GCS and NIHSS score, blood pressure, ICH location, ICH volume, SAH presence, HC, midline shift, etiology: aneurysm, AVM, moyamoya, cavernous malformation, surgical intervention | IVH severity measured by mGraeb score independently predicts death and poor functional outcome in patients with VSARICH. Key Words:  Intracerebral hemorrhage—vascular structural abnormalities—intraventricular  hemorrhage—severity—prognosis. | AVM cases |
| Zahunarec et al., 2007^77^  USA | Total: 270  Early C-DNR: 93  No-early C-DNR:177 | Single center, retrospective | All cases of nontraumatic ICH in Nueces County, TX, were  identified from January 1, 2000, through December 31, 2003  44 years old with possible  stroke among patients presenting with stroke symptoms or  diagnosis in hospital emergency department or admissions  logs.  only the first event was included in this analysis | Factors associated with early C-DNR  Discharge disposition: home, rehabilitation hospital, nursing home, hospice, deceased | Not stated | Not stated | Infratentorial vs supratentorial hemorrhage | Gender, race, median initial GCS, ICh vol (cc), infratentorial, IVH, HC | Early care limitations are independently associated with both short- and long-term  all-cause mortality after intracerebral hemorrhage (ICH) despite adjustment for expected predictors of  ICH mortality. Physicians should carefully consider the effect of early limitations in aggressive care to  avoid limiting care for patients who may survive their acute illness. | No comparison between ICH, ICH+IVH, ICH+IVH+Hydrocephalus |
| Zaidi et al., 2015^78^  USA | Total: 471  Shunt: 324  No-shunt: 147 | Multicenter, prospective RCT | age 18 to 80  years and acute nontraumatic subarachnoid hemorrhage (SAH), confirmed  by computed tomography (CT) or lumbar puncture |  |  |  |  |  | There is no difference in shunt dependency after SAH among patients  treated by endovascular or microsurgical means. Patients in whom shunt-dependent  hydrocephalus does not develop after SAH tend to have improved long-term functional  outcomes. | Subarachnoid hemorrhage  Shunt dependent hydrocephalus, no comparison between ICH, ICH IVH, ICH+IVH+Hydrocephalus |
| Zhang et al., 2016^79^  China | Total:65  Vascular structural abnormality PIVH:34  Idiopathic: 31 | Single center, retrospective | adult patients who had  intraventricular hemorrhage without recognizable intraparenchymal or subarachnoid hemorrhage on initial computed tomography (CT) scan underwent routine thin-slice  CT scan (2 mm)  Patients with intraparenchymal hemorrhage or subarachnoid hemorrhage on  thin-slice CT scan were excluded | Factors related to development of PIVH and idiopathich PIVH  30-days functional outcome | Hypertension, hyperlipidemia, diabetes, smoking, alcoholism | Not stated | Not stated | Comorbidities, initial GCS score, Graeb score, hydrocephalus, etiological cause | overall mortality rate was 11.9%, and 21 patients (31.3%)  had a poor outcome at the 6-month follow-up. Patients with  younger age, lower Graeb score, and a known etiology of  arteriovenous malformation might be associated with a  favorable outcome. We recommended routine thin-slice  computed tomography (CT) scan, computed tomographic  angiography (CTA), and digital subtraction angiography  (DSA) for patients with PIVH. The etiological causes and  prognostic factors of PIVH in Chinese patients were  associated with distinctive features. | Primary IVH |
| Bender et al., 2020^80^  Germany | Total: | Retrospective study | **Inclusion:**  Spontaneous Ich patients  Admitted between Fe 2008 to Dec 2017 treated for ate least 24h  Diagnosis established by Ct  **Exclusion:**  Trauma, vascular malformation, neoplasia, present of acute or chronic liver failure, age <18years | Intra hospital outcome and mortality  mRS at discharge | Hypertension  COPD  Cardiac Arrhytmia  Coronary heart disease  Heart failure  History of cardiac/cardiosurgical intervention  Chronic renal insufficiency  DM  History of ischemic stroke  History of ICH  Cancer | EVD  Evacuation  Decompressive carniectomy | Supratentorial lobar  Supratentorial deep  Infratentorial | Localization, IVH, Hydrocephalus,  Biomarkers  Cardiopulmonary parameters | A CRP/albumin ratio greater than 1.22 upon admission was significantly associated  with intra-hospital mortality in the ICH patients | Undefined period of in hospital mortality |
| Doukas et al,, 2015^81^  Germany | Total: 57 | Randomized controlled trial | **Inclusion:**  Patients with cerebral hematoma  **Exlusion:** Trauma, hemorrhage in brainstem, SAH, AVM | 6-weeks functional outcome (GOS 1-3 vs 4-5) | Hypertension  Brain ischemia  Tumor  MI  Diabetes  Thrombosis  Embolism  Intracerebral hemorrhage | Hematoma evacuation  Ventricle drainage | Cerebral | Gender, aage, GCS, blood clot volume  Diameter hemorrhage | We introduced as a new factor that is, the cerebellar hemorrhage/PF  ratio and found out that the value >35% was associated to an unfavorable outcome | Period of follow-up not matched with other studies |
| Kongwad et al., 2018^82^ | Total 455 |  | **Inclusion:**  + sICH patients admitted from Feb 2015 – Jul 2017  **Exclusion:**  + Trauma  + AVM  + Aneurysm  + Coagulation abnormalities | Discharge functional outcome  3-mo functional outcome  90-day mortality |  |  |  |  | Admission blood glucose levels was not an independent predictor of  mortality in our study when adjusted with age, GCS, and hematoma volume. The effect  of high ABG on SICH outcome is probably multifactorial and warrants further research |  |
| Lee et al., 2019^83^  Taiwan | Total 47 | Retrospectie study | **Inclusion:**  Patients with SCH admitted 2007-2011  **Exclusion:**  Traumatic cerebellar hemorrhage  Supratentorial, brainstem, SAH  Arterioenous vascular malformation  Subdural hematoma  Hemorrhagic infarct  Inflammatory vascular disese  Spontaneous cerebellar hemorrgae due to tumor  Prexisting neurological donciditon with various neurological deficits | GOS at 2 years | Not stated | Not specified | Cerebellar | Age, sex GCS on admission, pre-operative GCS, NIHSS at discharge, hematoma volume | An increase of one point in a patient’s NIHSS score at discharge following neurological surgery will  increase the probability of a poor two-year postoperative outcome by 28.5% | No data of IVH and HC to outcome |
| Moullaali 2016^84^ | Total; 1310  No IVH 854  Initial IVh 349  Delayed IVH 107 | Prospective randomized tiral | Inclusion:  sICH within 6h of onset  Elevated SBP | 90-day functional outcome (mRS) | DM  Ischemic stroke history  Hypertension  Antihypertensive  Warfarin  Spirin  Lipid lowering therapy | Not stated | Supratentorial | Age  Sex  China region  Time to diagnostic  NIHSS score  Location  ICH volume | Although linked to factors determining  greater ICH growth including poor SBP control, dIVH is  independently associated with poor outcome in acute  small to moderate-size ICH | Uncertain case of IVH without hydrocephalus |
| Oie et al., 2018^85^ | Total: 452 |  | **Inclusion:**  Patients with sICH  Age 18+  **Exclusion:**  Traumatic ICH  ICH related to tumors, extra-axial intracranial hemorrhages, thrombolytic treatment  Isolated IVH | mRS score at 12 months | HTN  Atrial fibrillation  DM  HYperlipidemia  Ischemic heart disease  Congestive heart disease  Previous ischemic/hemorrhagic stroke  Venous thromboembolism  Peripheral vascular disease  Dementia  Renal failure  CODP  Peptic ulcer | Hematoma evacuation  EVD  Hemicraniectomy | Lobar  Deep  Brainstem  Cerebellum  Strict IVH | Age  Sex  Comorbidity index  Oral antithrombotic drugs  mRS before stroke  GCS on admission  Locatin  Volume | Intracerebral hemorrhage is associated with high mortality, and more  than one third of survivors end up with severe disability or death 3 months later.  Predictors of severe disability or death were use of oral antithrombotic drugs, func‐  tional disability prior to ICH, low GCS on admission, larger hematoma volume, and  intraventricular hematoma extension. | No data on number of patients with IVH and HC (odds ratio only) |
| Peng et al., 2010^86^ | Total:  423 | Retrospective | **Inclusion**  Admitted to Taichung Veteran General Hospital during 2006-2008  sICH confirmed within 24h | 30-day mortality | HTN  DM  Ischemic heart disease  Previous stroke  Anemia  Dialysis | Not stated | Basal ganglia  Cerebellum  Pons  Thalamus  Lobar  Multiple | Age  Gender  Systolic BP  Diastolic BP  Arterial pressure  Location  Site  Ich volume  Pineal shift  Hb  Glucose | The RF provided the best predictive performance amongst all of the  tested models. We believe that the RF is a suitable tool for clinicians to use in predicting the 30-day mortality of patients after SICH. |  |
| Safatli et al., 2016^87^ | Total: 342 | Retrospective | **Inclusion**  Patients admitted with primary sICH between 2005-2013  **Exclusion:**  Secondary Ich by trauma, tumors, AVM, aneurysm  Insufficient medical records  Absence of initial brainstem reflexes | 30-day mortality | HTN  DM  Smoking  Alcohol | Surgery  Conservative | Basal ganglia  Lobular  Cerebellum  Brainstem | Gender  Comorbidities  AC/AP  Location  Presence of IVH  Presence of SAH  Midline shift  Dept | GCS score on admission together with the baseline volume and  localization of the hemorrhage are strong predictors for 30‑day mortality in patients  with spontaneous primary intracerebral hemorrhage, and by relying on them it is  possible to identify high‑risk patients with poor short‑term outcome. The ICH score  and the ICH‑GS accurately predict the 30‑day mortality. | Uncertain case of IVH without hydrocephalus |
| Satopaa 2017^88^ | Total: 347 | Retrospective | **Inclusion**  Patients admitted between 2005-2010  **Exclusion:**  AVM, dural fuistulae, tumors | mRS at discharge  (Good: 1-3, Poor: 4-6) | HTN  DM  Atrial Fibrilation  Liver disease | Surgical not specified | Not specified | Ge  GCS at arrival  Volume  Diameter  Extension to brainstem  Ventricular blood | Surgical treatment of cerebellar ICH can be life‑saving but often  leads to a poor functional outcome. New studies are needed on long‑term functional  outcome after a cerebellar ICH. | Undefined period of in hospital outcome |
| Szepesi 2015^89^ | Total: 156 | Retrospectiv | Inclusion:  Primary supratentorial ICH admitted in 53-mo period  Age 18+  **Exclusion**  Traumatic Ich  SAH  Vascular malvormation  Tumour  Hemorrhagic transformation of ischaemic stroke  Postthrombolytic hemorrhage  Infratentorial Ich  Primary IVH  Already undergone neurosurgical evacuation | 30-day fatality | Alcohol  Smoking | Not stated | Supratentorial | Volume  Hematoma growth index | After validation the SUSPEKT score may be applicable in general  clinical practice for early patient selection to optimize individual management or for assessment of eligibility for treatment trials. | No data on number of patients with IVH |
| Tao 2016^90^ | Total: 77 | **Retrospective** | **Inclusion**  Admitted within 24h after onset between Sep 2010 and April 2015  **Exclusion:**  Head trauma  Coagulopathy  Warfarin therapy  Cerebral venous thrombosis  Hemorrhagic transformation from ischemic stroke  AVM  Aneurysm  Tumor apoplexy | 6-moth functional outcome (mRS <2 good, >2 poor) | HTN  DM  Drinking  Smoking | Surgery | Supratentorial | Presence of SAH  IVH  FVC  BSC  Hydrocephalus  Hematoma size | To the best of our knowledge, this is the first study focusing on the relationship between hyperglycemia and  long-term functional outcome after CH. The study combined with previous pertinent reports definitely indicates the poor effect of hyperglycemia on both supra- and infratentorial ICH independent of hemorrhage site.  Therefore, further controlled trials are urgently needed to evaluate the benefits of glucose-lowing treatment. |  |
| Weimar 2006^91^ | Total: | Prospective | **Inclusion:**  Admitted within 6h after onset  Prior rankin grade <2 | 100-days mortality, Barthel Index <70, moderate dependence BI 70-90, Independence BI 95 | Prior stroke  Coronary heart disease  HTN  DM  Coagulation | Not state | Thalamic  Putaminal  Cerebellar  Brainstem  Lobar  Lenticulostriate | Age  GD  Aial diameter  Location  Bleeding or hydrocephalus | Our  study provides a validated prognostic model for prediction of  complete recovery following ICH  which could be very useful for the  design of clinical studies. | No data on number of patients (model only) |
| Yang 2000^92^ | Total: | Prospective | The inclusion criteria were: (1) a CT scan  was performed within 6 h after symptoms onset; and (2)  a follow-up CT scan was performed within 36 h after  baseline CT. Patients were excluded from the study if the  hemorrhage belongs to primary IVH, warfarin-associated  bleeding, secondary ICH due to trauma or tumor | 30-day mortality  90-day mortality  90-day mRS  (0-3 good, 4-6 poor) | Alcohol  Smoking  DM  HTN  Antiplatelet  Renal Dysfunction | Surgical  Decompression  EVD | Basal ganglia  Thalamic  Lobar  Infratentorial | Age  Sex  Alcohol  SSmoking  Comorbidities  Time from onset  ICH volume  Hematoma expansion  TVH  FVH  IVH volume  IVH growth  HC score  Presence of initial HC | Hydrocephalus growth is defned by strongly predictive of short‑ or long‑term mortality and poor  outcome at 90 days, and might be a potential indicator for assisting clinicians for clinical decision‑making | Uncertain case of IVH without hydrocephalus |
| Zis 2014^93^ | Total: 191 | Retrospective | **Inclusion:**  Admitted to Departmen of Neurology between Jan 2011 and June 2013  Primary inoperable ICH | 30-day fatality | HTN  Smoking  Alcohol  Anticoagulant | Not stated | Basal ganglia  Lobar  Cerebellar  Thalamic  Brainstem  Infratentorial  IVE | Gender  Age  Comorbidities  GCS  Location  Laboratory findings | GCS score on admission, infratentorial location of the hematoma, intraventricular  extension of the hematoma, INR on admission, and maximum diameter of the hematoma are the 5 variables that are independently associated with 30-day case fatality  of primary inoperable ICH. EDICH is introduced as a new grading scale, which includes laboratory and clinical findings at the ED and has predicting value of the  30-day case fatality. | Uncertain case of IVH without hydrocephalus |
| Di Napoli 2012^94^ | Total 223 | Retrospective study | **Inclusion:**  + sICh patients admitted to ICU 2010 international center  + Absence of trauma  + Absence of structural lesion  **Exclusion:**  + History of acute or chronic infections  + Evidence of acquired in hospital infection | 30-day mortality  30-day poor outcome | + Arterial hypertension  + Diabetes mellitus  + Alcohol abuse  + Smoking  + Hypercholesterolemia  + Anticoagulant |  | Supratentorial infratentorial | + Location  + IVH  + Midline shift  + Hydrocephalus  + Surgery  + CRP admission + CRP 24h  + CRP 48h  + CRP 72 h  + Blood glucose  + WBC |  | Uncertain case of IVH without hydrocephalus |
| Biller et al., 1987^95^ | total 95 | Retrospective study | **inclusion**  + Non traumatic SAH  + Age 15-45  + Identified by CT | Number of patients with misdiagnoses  Site of ruptured aneurysm  CT classification  Mortality |  |  | Cerebral  Cerebellar |  | The overall mortality was 8.4% with deaths due to neurological causes | SAH |
| van Gijn et al., 1985^96^ | Total 174 patients | Prospective cohort | Inclusion:  + Symptoms and signs of SAH  + Clinical examination and CT scanning performed within 72h  + Presence of aneurysm proven by angiography or autopsy  Ex:  + Died within 24h | Incidence of acute hydrocephalus  Consiousness |  |  | All |  | Hydrocephalus was found in 34 of 174 prospectively studied patients with SAH who survied the first 24h. The occurrence of acute hydrocephalus was related to the presence of IVC blood, and not to extent of cisternal hemorrhage. Death from infarction could not be attributed to the extent of cisternal hemorrhage, the use of antifibrinolytic drugs, failure to apply surgical drainage | SAH |
| Hyun et al., 1997^97^ | Total 419 | Retrospective | **Inclusion:**  + Patients with SAH due to aneurysmal rupture | Mortality rate  Mental status | Functional Outcome Survival and Independent Risk Factors in Patients with Spontaneous Intracerebral Hemorrhage from Chronic Arterial Hypertension |  | All |  | Functional Outcome Survival and Independent Risk Factors in Patients with Spontaneous Intracerebral Hemorrhage from Chronic Arterial Hypertension | SAH |
| Kang et al., 2010^98^ | Total 33 | Prospective | **Inclusion**  + patients with initial fisher grade (3/4) SAH who had undergone conversion from external EVD to VP  Exclusion  Patients died of SHA complications after VP shunt placement | Perioperative data  Shunt malfunction rate |  |  | All |  | Based on our data, earlier EVD weaning and shunt placement can effectively treat subarachnoid hemorrhage–induced hydrocephalus in patients with severe subarachnoid hemorrhage. This procedure resulted in no shunt-related infections and a 6.1% revision rate. There were fewer adverse effects of IVH and protein on shunt performance. Therefore, weaning from an EVD and conversion to a permanent VP shunt need not be delayed because of IVH or proteinaceous CSF. | SAH |
| Lagares et al., 2001^99^ | 294 | Retrospective | **Inclusion**  Patients diagnosed with aneurysmal SAH | Functional outcome 1 month after discharge | Glasgow outcome scale |  |  | +Gender  +HTN  + loss of consciousness  + seizure  +Cranial nerve palsy  + WFNS grade  + Fisher grade  + CT brain hypodensity  + IVH  + Intraparenchymal blood  + Hydrocephalus | Age and clinical grade on admission are the most important factors influencing the final outcome of patients suffering aneurysmal SAH. A reappraisal of the WFNS grading scale should be considered as no significant differences in outcome were found between some of its grades. | SAH |
| Navi et al., 2010^100^ | 181 ICH  46 SAH | Retrospective | **Inclusion:**  Adult cancer patients with intracranial hemorrhage | 3-mo mortality  30-day mortality  Discharge functional outcome |  |  |  | + Tumor type  + Etiology | Sixty-eight percent of patients had solid tumors, 16% had primary brain tumors, and 16% had hematopoietic tumors. Hemiparesis and headache were the most common symptoms. Intratumoral hemorrhage (61%) and coagulopathy (46%) accounted for the majority of hemorrhages, whereas hypertension (5%) was rare. Median survival was 3 months (95% confidence interval [CI] 2-4), and 30-day mortality was 31%. However, nearly one-half of patients were completely or partially independent at the time of discharge. Patients with primary brain tumors had the longest median survival (5.9 months, 95% CI 2.9-11.8, p = 0.05). Independent predictors of 30-day mortality were not having a primary brain tumor, impaired consciousness, multiple foci of hemorrhage, hydrocephalus, no ventriculostomy, and treatment of increased intracranial pressure. | SAH |
| Paisan et al., 2017^101^ | 116 | Retrospective | **Inclusion**  Patients with aSAh and had shunt-dependent hydrocephalus | Functional outcome | mRS  0-2 good  3-6 bad |  |  | + Shunt complications  + Presence of IVH  _ Gender  + Age  + GCS  + Fisher grade  + Hunt Hess Grade  + WFNS grade  + Aneurysm location  + Treatment | aSAH patients with shunt-dependent hydrocephalus have significantly poorer long-term functional outcomes. Patients with risk factors for post-aSAH shunt dependence may benefit from increased surveillance, although the effect of such measures is not defined in this study. | SAH |
| Pasqualin et al., 1986^102^ | 38 patients | Retrospective | **Inclusion:**  patients with symptomatic cerebral aneurysm or spontaneous SAH | Mortality  Results of treatment |  |  |  | + Location  + Hematoma  + rebleeding  _Hydrocephalus  + Spasm  + Treatment  + Time to surgery  Etiology |  | SAH |
| Sheikazadi et al., 2009^103^ | 85 women 60 ment | Retrospective | **Inclusion:**  Cadavers who were diagnosed with aneurysmal SAH between 2001-2005 | Incidence of sudden death and remnant |  |  |  | +Gender  +HTN  + Smoking  + Family history of aneurysmal SAH  + Alcohol  + Arteriosclerosis  + IVH  + Edema  + Location  +ICH | In our population, the frequency of sudden death from aneurysmal SAH has not changed during the last 5 years. The typical clinical profile of sudden death in SAH includes intraventricular hemorrhage, pulmonary edema, and a ruptured posterior circulation aneurysm. Intracerebral hemorrhage is rarely connected to sudden death from aneurysmal SAH. | SAH |
| Wang et al., 2012^104^ | 168 | Retrospectiv | **Inclusion:**  Patients with aneurysmal SAH with hydrocephalus shunt-dependency | Functional score | Glasgow outcome scale |  |  | + GCS  + Fisher SAH grade  + WFNS grade  + Location  + Shape of aneurysm  + Complications  + | The presence of intra-ventricular hemorrhage, lower mean Glasgow Coma Scale score, and higher mean scores of the modified Fisher SAH and World Federation of Neurosurgical grading on admission imply risk of shunt-dependent hydrocephalus in patients without initial hydrocephalus. These patients have worse short- and long-term outcomes and longer hospitalization. | SAH |
| Zhao et al., 2015^105^ | 47 | Retrospective | **Inclusion:**  Patients with WFNS grade IV and V underwent surgical treatment  ultra early surgery (within 24h) vs delayed surgery (>24h) | Functional outcome | Glasgow outcome scale |  |  |  | Although patients with WFNS grade V and brain herniation more often undergo ultra-early surgery, postoperative complications and outcomes in selected patients were similar in the two groups. Patients of younger age, WFNS grade IV, absence of intraventricular haemorrhage, absence of brain herniation and MCA aneurysms are more likely to have a good outcome. Ultra-early surgery could improve outcomes in carefully selected patients with poor-grade aSAH. | SAH |

**Supplemental Table II.** Newcastle Ottawa Scale for Included Studies

| **Name of Study (Year)** | **Selection** | | | | **Comparability** | **Outcome** | | | **Total Score** |
| --- | --- | --- | --- | --- | --- | --- | --- | --- | --- |
|  | Representativeness of the exposed cohort  (a) truly representative of the average ICH patients in the community, national or multicenter studies*  (b) somewhat representative for the ICH patients in the community, single center studies*  (c) selected group of users  (d) no description of the derivation of the cohort | Selection of the non-exposed cohort  (a) drawn from the same community as the exposed cohort*  (b) drawn from different source  (c) no description of the derivation of the non-exposed cohort | Ascertainment of exposure  (a) secure record*  (b) structured interview*  c) written self report  (d) no description | Demonstration that outcome of interest was not present at start of study  (a) yes*  (b) no | Comparability of cohort on the basis of the design or analysis | Assessment of outcome  (a) Independent assessment*  (b) Record linkage*  (c) Self-report  (d) No description | Was follow-up long enough for outcomes to occur  (a) yes (1-month minium follow-up)* (b) no | Adequacy of follow-up of cohort  (a) complete follow-up, all subjects accounted for*  (b) subjects lost to follow-up are unlikely to introduce bias – small number lost <20%  (c) follow-up rate <80% and no description of those lost  (d) no description *or unclear* |  |
| Al Safatli 2017 | This is a single‑center retrospective study (b)*  LOW | We retrospectively analyzed data from 50 consecutive patients treated in our tertiary academic centre between January 2005 and December 2014, all diagnosed with a first episode of isolated SCH. (a)*  LOW | The initial imaging data, mainly head computed tomography (CT) scans (LightSpeed VCT, GE Medical Systems, Milwaukee, WI, USA), were reviewed to determine the location and dimension of each SCH (a)*  LOW | This is a single‑center retrospective study (a)*  LOW | Not applicable  This is a single‑center retrospective study  UNCERTAIN | The major end‑points in our study were death within 30 days, functional status at follow‑up (a maximum of 30 days after ictus) and recurrent cerebellar hemorrhage or ischemic stroke. Neurological outcome was measured using the modified Rankin scale (mRS). The initial neurologic status was determined according to the Glasgow Coma Scale (GCS)  score at the time of arrival of the patient in our emergency department and before the start of any treatment.  LOW | No follow up, retrospective study  UNCERTAIN | No follow up, retrospective study  UNCERTAIN | 5 |
| Appleboom 2011 | This is a single‑center prospective study (b)*  LOW | Between February 2009 and September 2010, patients with spontaneous ICH diagnosed by admission CT scan were admitted to the Columbia University Medical Center Neurological Intensive Care Unit and prospectively enrolled in the Intracerebral Hemorrhage Outcomes Project (ICHOP) (a)*  LOW | Patients with spontaneous ICH diagnosed by admission CT scan (a)*  LOW | This is a single‑center retrospective study (a)*  LOW | Not applicable as the main purpose of the study is to find predictors of mortality and functional outcome  UNCERTAIN | Admission blood glucose was defined as the first intravenous blood glucose level drawn at the time of the initial emergency department or in-hospital evaluation for ICH. Admission CT scan was evaluated for hematoma volume and location, presence and severity of IVH as assessed by the IVH score,11 degree of midline shift, and presence of hydrocephalus.  LOW | The secondary end points included 3-month mortality and the state of functional recovery at hospital discharge and 3 months post-ICH according to mRS.  LOW | (a) complete follow-up, all subjects accounted for*  LOW | 7 |
| Asadollahi 2016 | Consecutive patients with spontaneous, non-traumatic and non-neoplastic ICH admitted to the emergency department (n = 324) were prospectively studied. (b)*  LOW | Between January 2011 and May 2012, 324 consecutive patients with clinical signs of SICH were identified. After a comprehensive initial evaluation, 228 patients were included in the study. (a)*  LOW | Baseline CT examinations were conducted within 12 hours of initial evaluation using a multidetector row scanner (a)*  LOW | The post-SICH outcomes were determined as follows: 30-day mortality as the early outcome and level of disability and functional status at 36 months as the long-term outcomes evaluated using mRS and BI, respectively. (a)*  LOW | Not applicable as the main purpose of the study is to find predictors of mortality and functional outcome  UNCERTAIN | CT scans were then independently reviewed by two experienced neuroradiologists who were blinded to all patients’ clinical information.  Neurological death was defined as mortality caused by direct consequence of a diagnosed SICH or a new haemorrhage in the absence of other intervening causes.  Functional scales were scored using **telephone interview** according to the patients’ or their caregivers’ report. Telephone interviews were performed by two physicians who were trained and certified for data collection on disability and quality-of-life (c)  HIGH | The post-SICH outcomes were determined as follows: 30-day mortality as the early outcome and level of disability and functional status at 36 months as the long-term outcomes evaluated using mRS and BI, respectively. (a)*  LOW | During 3 months follow up, 122/132 30-days survivors participated (b)  LOW | 6 |
| Bhatthairi 2016 | By the end of recruitment in February, 2003, 107 centres were registered with the trial and 1033 patients had been recruited.  Multinational study (a)*  LOW | Both groups were drawn from the same hospital with randomization (a)*  LOW | Patients were eligible for inclusion if they had CT evidence of a spontaneous supratentorial intracerebral haemorrhage that had arisen within 72 h  Secure records (a)*  LOW | Primary outcome was death or disability using the extended Glasgow outcome scale 6 months after ictus. (a)*  LOW | The groups were well matched at baseline. (a)*  LOW | **Questionnaires** were sent directly to the surviving patients or carers for completion at 6 months as a technique of masking surgeons to the outcome (c)*  HIGH | Questionnaires were sent to patients at 5 months for completion by the patient, relative, or carer, and a reminder was sent at 6 months (a)*  LOW | Another 43 patients were lost between the 2-week follow-up and 6-month followup. For a further 17 patients, their status at 6 months was not recorded but they were known to have died after 6 months.  LOW | 7 |
| Di Napoli  2011 | We prospectively recruited all consenting patients admitted to 2 intensive care units (a)*  LOW | All patients were drawn from the same hospital  LOW | sICH was defined as the sudden and spontaneous intraparenchymal bleeding confirmed by head CT scan (a)*  LOW | Outcome was assessed as mortality at 30 days after SICH  Outcome not present during admission of study (a)*  LOW | Not applicable as the main purpose of the study is to find predictors of mortality and functional outcome  UNCERTAIN | Investigators blinded to the results of biomarker testing collected all clinical, laboratory, and radiographic information, as well as the functional outcome category. (a)*  LOW | Outcome was assessed as 30-day mortality  LOW | We were able to obtain current information on all included patients.(a)*  LOW | 7 |
| Hedge 2020 | ­The study was approved by the Institute Ethics Committee, Manipal University (Approval No IEC 209/2015). All patients above the age of 18 years who were presented to the Emergency Department between 1st January 2015 and 31st December 2018 (b)*  LOW | All patients were drawn from the same hospital  LOW | … with computerised tomography (CT) evidence of SICH were included in the study (a)*  Secure records  LOW | ­The outcome determinants were mortality and morbidity as measured using the modified Rankin Scale (mRS) at discharge and three months  Outcome not present during admission of study (a)*  LOW | Not applicable as the main purpose of the study is to find predictors of mortality and functional outcome  UNCERTAIN | Ninety day mRS was recorded in the **outpatient clinic**. In cases where the patient failed to visit the clinic, a telephonic mRS was recorded  (a) Independent assessment*  LOW | Ninety day mRS was recorded in the outpatient clinic. In cases where the patient failed to visit the clinic, a telephonic mRS was recorded (a)*  LOW | Data were available for all 619 surviving patients  (a) complete follow-up, all subjects accounted for*  LOW | 7 |
| Liliang 2000 | We retrospectively reviewed medical records of all patients who had a diagnosis of spontaneous ICH at Koahsiung Chang Gang Memorial Hospital during the period January 1995 to December 1999 (b)*  LOW | All patients were drawn from the same hospital  LOW | We diagnosed caudate hemorrhage when CT scan revealed a highdensity area mainly at the head of the caudate nucleus contiguous to the anterior horn of the lateral ventricle with or without intraventricular extension. Thirty-six consecutive cases with hypertensive caudate hemorrhage were included in this study.  Secure records (a)*  LOW | Neurological outcome for the survivors was determined at 6 months after hemorrhage.  Outcome not present during admission of study (a)*  LOW | Not applicable as the main purpose of the study is to find predictors of mortality and functional outcome  UNCERTAIN | Outcome was categorized by use of the Glasgow Outcome Scale as asymptomatic, mild disability, moderate disability, major disability, vegetative status, and death  Some patients were followed by the outpatient department after discharge, and others were interviewed by telephone to identify neurological outcome.  Most likely most of the patients were interviewed (c)  HIGH | Neurological outcome for the survivors was determined at 6 months  LOW | (a) complete follow-up, all subjects accounted for*  LOW | 6 |
| Mansouri 2013 | One hundred and twenty consecutive patients with nontraumatic and non-aneurysmatic ICH who presented to Emergency Department of Imam Hossein Medical Center between January and July 2012 (b)*  LOW | All patients were drawn from the same hospital  LOW | All cases were diagnosed prospectively based on full neurologic examination, computed tomographic (CT) findings of the brain within 24 h compatible with ICH  Secure records (a)*  LOW | The primary outcome variable was the 30-day mortality for neurological cause.  The secondary end points included 3-month mortality and the state of functional recovery at hospital discharge and 3 months post-ICH according to mRS.  Outcome not present during admission of study (a)*  LOW | Not applicable as the main purpose of the study is to find predictors of mortality and functional recovery  UNCERTAIN | The mRS is one of the most frequently used scale to measure outcome in patients with ICH and demonstrates level of disabilities with a special emphasis on the patient’s physical ability to walk. All outcome variables were **validated by a study physician**.  (a) Independent assessment*  LOW | The secondary end points included 3-month mortality and the state of functional recovery at hospital discharge and 3 months post-ICH according to mRS.  LOW | (a) complete follow-up, all subjects accounted for*  LOW | 7 |
| Roeder 2019 | All consecutive patients with spontaneous ICH admitted in the Department of Neurology, University Hospital Erlangen, Germany, were included in our prospective institutional registry (b)*  LOW | All patients were drawn from the same hospital  LOW | Baseline characteristics were retrieved from medical charts and electronic patient records as described previously. Laboratory findings were extracted from the institutional laboratory database.  Secure records (a)*  LOW | We assessed mortality and functional outcome after 3 months  Outcome not present during admission of study (a)*  LOW | In light of these imbalances in baseline clinical characteristics we performed a one-to-one propensity score matching after which there were 173 evenly balanced patients in each cohort with no relevant discrepancies in baseline characteristic  Propensity score matching were done for ICH vs IVH (a)*  LOW | We assessed mortality and functional outcome after 3 months either by mailed questionnaires or semi-structured telephone interview performed by physicians certified for stroke outcome assessments.  (c) Self-report  HIGH | We assessed mortality and functional outcome after 3 months (a)*  LOW | (a) complete follow-up, all subjects accounted for*  LOW | 7 |
| Stein 2010 | Hospital charts of patients who were admitted with SICH to the Department of Neurosurgery of the University Hospital Giessen between January 1995 and December 2002 (b)*  LOW | All patients were drawn from the same hospital  LOW | Initial CT scans were available for all patients.  Secure records (a)*  LOW | Outcome was examined as 30-day mortality and 6-month functional outcome.  Outcome not present during admission of study (a)*  LOW | Not applicable as the main purpose of the study is to find predictors of mortality and Glasgow outcome scale  UNCERTAIN | Outcome was examined as 30-day mortality and 6-month functional outcome. Functional outcome was assessed by the modified Rankin Scale (mRS).  (d) Method of assessment for mRS was not stated  UNCERTAIN | Outcome was examined as 30-day mortality and 6-month functional outcome. (a)*  LOW | After 3 and 6 months, only 2 and 4 of 110 SICH patients were lost to follow-up, respectively. (b)*  LOW | 6 |
| Kongwad 2018 | This was a retrospective observational study conducted at Kasturba Hospital Manipal, a tertiary care center in the small coastal town in of Udupi, Karnataka, India (b)*  LOW | All patients were drawn from the same hospital  LOW | Secure records (a)*  LOW | The primary outcome was mortality and functional outcome at 90 day was not present during admission(a)*  LOW | Not applicable as the main purpose of the study is to find predictors of mortality and Glasgow outcome scale  UNCERTAIN | Telephone assessment mRS, shown to have good inter-rater reliability was used for patients who did not present to the clinic at 3 months  (a) Independent assessment  LOW | Functional independence was assessed at 90day using modified Rankin Scale (a)*  LOW | 12 out of 510 patients lost to follow up  (b) subjects lost to follow-up are unlikely to introduce bias – small number lost <20%*  LOW | 7 |
| Peng et al., 2010 | We retrospectively studied 423 patients admitted to the Taichung Veterans General Hospital during a 3-year period (b)*  LOW | All patients were drawn from the same hospital  LOW | Secure records (a)*  LOW | The primary outcome was mortality at 30 day was not present during admission(a)*  LOW | Not applicable as the main purpose of the study is to find predictors of mortality  UNCERTAIN | Information on these patients was collected exclusively from the database of electronic health records.  (b) Record linkage  LOW | No follow up, retrospective study  UNCERTAIN | No follow up, retrospective study  UNCERTAIN | 7 |
| Tao et al. 2016 | The patients were consecutively admitted to our institution between September 2010 and April 2015 (b)*  LOW | All patients were drawn from the same hospital  LOW | Secure records (a)*  LOW | The primary outcome was functional outcome at 6 month was not present during admission(a)*  LOW | Not applicable as the main purpose of the study is to find predictors of mortality and Glasgow outcome scale  UNCERTAIN | (b) Record linkage  LOW | No follow up, retrospective study  UNCERTAIN | No follow up, retrospective study  UNCERTAIN | 7 |

**Supplemental Table III**. Summary of meta-analysis statistics

| **Outcome** | **Risk Ratio (95% CI),**  **p-value** | **Heterogeneity**  **(I^2^),**  **p-value** | **Egger**  **p-val** | **Habord p-val** | **Peter**  **p-val** | **Number of Studies** |  |
| --- | --- | --- | --- | --- | --- | --- | --- |
| **ICH + IVH vs ICH** |  |  |  |  |  |  |  |
| 30-days Mortality |  |  |  |  |  |  | |
| Summary | 2.19 [1.58, 3.04], <0.00001 | 17%, 0.31 | 0.622 | 0.212 | 0.579 | 5 | |
|  |  |  |  |  |  |  | |
| 90-days Mortality | | | | | | | |
| Summary | 1.58 [1.20, 2.09], **0.001** | 63%, **0.003** | 0.388 | 0.763 | 0.661 | 5 | |
|  |  |  |  |  |  |  | |
| 3-Month Good Functional Outcome | | | | | | | |
| Summary | 0.83 [0.60, 1.14], 0.24 | 80%, **0.007** | 0.425 | 0.873 | 0.694 | 3 | |
|  |  |  |  |  |  |  | |
| 6-Month Good Functional Outcome | | | | | | | |
| Summary | 0.62 [0.47, 0.81], **0.0005** | 51%, 0.150 | NA | NA | NA | 2 | |
|  |  |  |  |  |  |  | |
| **ICH + IVH + HC vs ICH** |  |  |  |  |  |  | |
| **30-days Mortality** |  |  |  |  |  |  | |
| **Summary** | 4.26 [2.35, 7.72], **<0.00001** | 80%, **0.002** | 0.657 | 0.437 | 0.127 | 4 | |
|  |  |  |  |  |  |  | |
|  |  |  |  |  |  |  | |
| **90-days Mortality** |  |  |  |  |  |  | |
| **Summary** | 2.30 [1.99, 2.67], **<0.00001** | 37%, 0.19 | 0.111 | 0.854 | 0.854 | 4 | |
|  |  |  |  |  |  |  | |
| **3-Months Good Functional Outcome** | | | | | | | |
| **Summary** | 0.66 [0.36, 1.19], 0.16 | 91%, **0.0010** | NA | NA | NA | 2 | |
|  |  |  |  |  |  |  | |
| **6-Months Good Functional Outcome** | | | | | | | |
| **Summary** | 0.38 [0.28, 0.53], **<0.00001** | 0%, 0.71 | NA | NA | NA | 2 | |
|  |  |  |  |  |  |  | |
| **ICH + IVH + HC vs ICH + IVH** | | | | | | | |
| **30-days Mortality** |  |  |  |  |  |  | |
| **Summary** | 1.96 [1.47, 2.61], **<0.00001** | 6%, 0.37 | 0.373 | 0.399 | 0.091 | 5 | |
|  |  |  |  |  |  |  | |
| **90-days Mortality** |  |  |  |  |  |  | |
| **Summary** | 1.53 [1.29, 1.82], **<0.00001** | 69%, **0.002** | 0.554 | 0.817 | 0.825 | 4 | |
|  |  |  |  |  |  |  | |
| **3-Months Good Functional Outcome** | | | | | | | |
| **Summary** | 0.76 [0.62, 0.95], **0.01** | 0% 0.76 | NA | NA | NA | 2 | |
|  |  |  |  |  |  |  | |
| **6-Months Good Functional Outcome** | | | | | | | |
| **Summary** | 0.54 [0.40, 0.74], **<0.0001** | 0%, 0.40 | 0.254 | 0.795 | 0.664 | 4 | |
|  | | | | | | | |

NA = Not Applicable

**Supplemental Table IV.** GRADE Summary of Findings for ICH+IVH vs ICH

| **Quality assessment** | | | | | | | **Summary of Findings** | | | | |
| --- | --- | --- | --- | --- | --- | --- | --- | --- | --- | --- | --- |
| **Participants (studies) Follow up** | **Risk of bias** | **Inconsistency** | **Indirectness** | **Imprecision** | **Publication bias** | **Overall quality of evidence** | **Study event rates (%)** | | **Relative effect** (95% CI) | **Anticipated absolute effects** | |
|  |  |  |  |  |  |  | **With ICH** | **With ICH+IVH** |  | **Risk with ICH+IVH** | **Risk difference with ICH** (95% CI) |
| **30-day mortality** | | | | | | | | | | | |
| 781 (5 studies) | no serious risk of bias | no serious inconsistency | no serious indirectness | no serious imprecision | undetected | ⊕⊕⊕⊝ **MODERATE**^1^ due to plausible counfounding would change the effect | 78/616  (12.7%) | 44/165  (26.7%) | **RR 2.19**  (1.58 to 3.04) | **Study population** | |
|  |  |  |  |  |  |  |  |  |  | **127 per 1000** | **151 more per 1000** (from 73 more to 258 more) |
|  |  |  |  |  |  |  |  |  |  | **Moderate** | |
|  |  |  |  |  |  |  |  |  |  | **118 per 1000** | **140 more per 1000** (from 68 more to 241 more) |
| **90-day mortality** | | | | | | | | | | | |
| 1611 (4 studies) | no serious risk of bias | no serious inconsistency | no serious indirectness | serious^2^ | undetected | ⊕⊕⊝⊝ **LOW**^1,2^ due to imprecision, plausible counfounding would change the effect | 262/1128  (23.2%) | 156/483  (32.3%) | **RR 1.41**  (1.19 to 1.68) | **Study population** | |
|  |  |  |  |  |  |  |  |  |  | **232 per 1000** | **95 more per 1000** (from 44 more to 158 more) |
|  |  |  |  |  |  |  |  |  |  | **Moderate** | |
|  |  |  |  |  |  |  |  |  |  | **248 per 1000** | **102 more per 1000** (from 47 more to 169 more) |
| **3-mo Functional Outcome** | | | | | | | | | | | |
| 1547 (3 studies) | no serious risk of bias | serious^3^ | no serious indirectness | no serious imprecision | undetected | ⊕⊕⊝⊝ **LOW**^1,3^ due to inconsistency, plausible counfounding would change the effect | 495/1084  (45.7%) | 165/463  (35.6%) | **RR 0.78**  (0.68 to 0.9) | **Study population** | |
|  |  |  |  |  |  |  |  |  |  | **457 per 1000** | **100 fewer per 1000** (from 46 fewer to 146 fewer) |
|  |  |  |  |  |  |  |  |  |  | **Moderate** | |
|  |  |  |  |  |  |  |  |  |  | **398 per 1000** | **88 fewer per 1000** (from 40 fewer to 127 fewer) |
| **6-mo Functional outcome** | | | | | | | | | | | |
| 725 (2 studies) | no serious risk of bias | no serious inconsistency | no serious indirectness | serious^2^ | undetected | ⊕⊕⊝⊝ **LOW**^1,2^ due to imprecision, plausible counfounding would change the effect | 213/544  (39.2%) | 45/181  (24.9%) | **RR 0.64**  (0.48 to 0.84) | **Study population** | |
|  |  |  |  |  |  |  |  |  |  | **392 per 1000** | **141 fewer per 1000** (from 63 fewer to 204 fewer) |
|  |  |  |  |  |  |  |  |  |  | **Moderate** | |
|  |  |  |  |  |  |  |  |  |  | **447 per 1000** | **161 fewer per 1000** (from 72 fewer to 232 fewer) |

^1^ Possible confounding factors: diabetes mellitus, coagulopathy, initial GCS score <8, ICH volume >30, midline shift, and EVD insertion
^2^ Risk Ratio and/or confidence interval <1.25 or >0.75
^3^ Heterogeneity (I2) > 50%

**Supplemental Table V.** GRADE Summary of Findings for ICH+IVH+HC vs ICH

| **Quality assessment** | | | | | | | **Summary of Findings** | | | | |
| --- | --- | --- | --- | --- | --- | --- | --- | --- | --- | --- | --- |
| **Participants (studies) Follow up** | **Risk of bias** | **Inconsistency** | **Indirectness** | **Imprecision** | **Publication bias** | **Overall quality of evidence** | **Study event rates (%)** | | **Relative effect** (95% CI) | **Anticipated absolute effects** | |
|  |  |  |  |  |  |  | **With ICH** | **With ICH+VH +HC** |  | **Risk with ICH** | **Risk difference with ICH +IVH + HC** (95% CI) |
| **30-day mortality** | | | | | | | | | | | |
| 824 (4 studies) | no serious risk of bias | very serious^1^ | no serious indirectness | no serious imprecision | undetected | ⊕⊕⊕⊝ **MODERATE**^1,2,3^ due to inconsistency, large effect, plausible counfounding would change the effect | 76/602  (12.6%) | 103/222  (46.4%) | **RR 4.26**  (2.35 to 7.72) | **Study population** | |
|  |  |  |  |  |  |  |  |  |  | **126 per 1000** | **412 more per 1000** (from 170 more to 848 more) |
|  |  |  |  |  |  |  |  |  |  | **Moderate** | |
|  |  |  |  |  |  |  |  |  |  | **116 per 1000** | **378 more per 1000** (from 157 more to 780 more) |
| **90-day mortality** | | | | | | | | | | | |
| 1473 (4 studies) | no serious risk of bias | no serious inconsistency | no serious indirectness | no serious imprecision | undetected | ⊕⊕⊕⊝ **MODERATE**^3^ due to plausible counfounding would change the effect | 249/1052  (23.7%) | 218/421  (51.8%) | **RR 2.3**  (1.99 to 2.67) | **Study population** | |
|  |  |  |  |  |  |  |  |  |  | **237 per 1000** | **308 more per 1000** (from 234 more to 395 more) |
|  |  |  |  |  |  |  |  |  |  | **Moderate** | |
|  |  |  |  |  |  |  |  |  |  | **251 per 1000** | **326 more per 1000** (from 248 more to 419 more) |
| **3-mo functional outcome** | | | | | | | | | | | |
| 1286 (2 studies) | no serious risk of bias | very serious^1^ | no serious indirectness | no serious imprecision | undetected | ⊕⊝⊝⊝ **VERY LOW**^1,3^ due to inconsistency, plausible counfounding would change the effect | 431/923  (46.7%) | 106/363  (29.2%) | **RR 0.66**  (0.36 to 1.19) | **Study population** | |
|  |  |  |  |  |  |  |  |  |  | **467 per 1000** | **159 fewer per 1000** (from 299 fewer to 89 more) |
|  |  |  |  |  |  |  |  |  |  | **Moderate** | |
|  |  |  |  |  |  |  |  |  |  | **463 per 1000** | **157 fewer per 1000** (from 296 fewer to 88 more) |
| **6-mo Functional Outcome** | | | | | | | | | | | |
| 775 (2 studies) | no serious risk of bias | no serious inconsistency | no serious indirectness | no serious imprecision | undetected | ⊕⊕⊕⊝ **MODERATE**^3^ due to plausible counfounding would change the effect | 213/544  (39.2%) | 35/231  (15.2%) | **RR 0.38**  (0.28 to 0.53) | **Study population** | |
|  |  |  |  |  |  |  |  |  |  | **392 per 1000** | **243 fewer per 1000** (from 184 fewer to 282 fewer) |
|  |  |  |  |  |  |  |  |  |  | **Moderate** | |
|  |  |  |  |  |  |  |  |  |  | **447 per 1000** | **277 fewer per 1000** (from 210 fewer to 322 fewer) |

^1^ Heterogeneity (I2) >75%
^2^ Risk Ratio > 4
^3^ Possible confounding factors: diabetes mellitus, coagulopathy, initial GCS score <8, ICH volume >30, midline shift, and EVD insertion

**Supplemental Table VI.** GRADE Summary of Findings for ICH+IVH+HC vs ICH+IVH

| **Quality assessment** | | | | | | | **Summary of Findings** | | | | |
| --- | --- | --- | --- | --- | --- | --- | --- | --- | --- | --- | --- |
| **Participants (studies) Follow up** | **Risk of bias** | **Inconsistency** | **Indirectness** | **Imprecision** | **Publication bias** | **Overall quality of evidence** | **Study event rates (%)** | | **Relative effect** (95% CI) | **Anticipated absolute effects** | |
|  |  |  |  |  |  |  | **With ICH + IVH** | **With ICH+IVH +HC** |  | **Risk with ICH + IVH** | **Risk difference with ICH + IVH + HC**(95% CI) |
| **30-day mortality** | | | | | | | | | | | |
| 482 (5 studies) | no serious risk of bias | no serious inconsistency | no serious indirectness | no serious imprecision^1^ | undetected | ⊕⊕⊕⊝ **MODERATE**^1,2^ due to plausible counfounding would change the effect | 42/168  (25%) | 133/314  (42.4%) | **RR 1.96**  (1.47 to 2.61) | **Study population** | |
|  |  |  |  |  |  |  |  |  |  | **250 per 1000** | **240 more per 1000** (from 118 more to 402 more) |
|  |  |  |  |  |  |  |  |  |  | **Moderate** | |
|  |  |  |  |  |  |  |  |  |  | **256 per 1000** | **246 more per 1000** (from 120 more to 412 more) |
| **90-day mortality** | | | | | | | | | | | |
| 752 (4 studies) | no serious risk of bias | serious^3^ | no serious indirectness | no serious imprecision | undetected | ⊕⊕⊝⊝ **LOW**^2,3^ due to inconsistency, plausible counfounding would change the effect | 114/331  (34.4%) | 218/421  (51.8%) | **RR 1.38**  (1 to 1.91) | **Study population** | |
|  |  |  |  |  |  |  |  |  |  | **344 per 1000** | **131 more per 1000** (from 0 more to 313 more) |
|  |  |  |  |  |  |  |  |  |  | **Moderate** | |
|  |  |  |  |  |  |  |  |  |  | **329 per 1000** | **125 more per 1000** (from 0 more to 299 more) |
| **3-mo Functional Outcome** | | | | | | | | | | | |
| 656 (2 studies) | no serious risk of bias | no serious inconsistency | no serious indirectness | very serious^1^ | undetected | ⊕⊝⊝⊝ **VERY LOW**^1,2^ due to imprecision, plausible counfounding would change the effect | 112/293  (38.2%) | 106/363  (29.2%) | **RR 0.76**  (0.62 to 0.95) | **Study population** | |
|  |  |  |  |  |  |  |  |  |  | **382 per 1000** | **92 fewer per 1000** (from 19 fewer to 145 fewer) |
|  |  |  |  |  |  |  |  |  |  | **Moderate** | |
|  |  |  |  |  |  |  |  |  |  | **385 per 1000** | **92 fewer per 1000** (from 19 fewer to 146 fewer) |
| **6-mo Functional Outcome** | | | | | | | | | | | |
| 551 (4 studies) | no serious risk of bias | no serious inconsistency | no serious indirectness | serious^1^ | undetected | ⊕⊕⊝⊝ **LOW**^1,2^ due to imprecision, plausible counfounding would change the effect | 63/205  (30.7%) | 58/346  (16.8%) | **RR 0.54**  (0.4 to 0.74) | **Study population** | |
|  |  |  |  |  |  |  |  |  |  | **307 per 1000** | **141 fewer per 1000** (from 80 fewer to 184 fewer) |
|  |  |  |  |  |  |  |  |  |  | **Moderate** | |
|  |  |  |  |  |  |  |  |  |  | **355 per 1000** | **163 fewer per 1000** (from 92 fewer to 213 fewer) |

^1^ Risk Ratio and/or confidence interval ~1.25 or 0.75
^2^ Possible confounding factors: diabetes mellitus, coagulopathy, initial GCS score <8, ICH volume >30, midline shift, and EVD insertion
^3^ Heterogeneity (I2) > 50%

**REFERENCES**

1. Al Safatli D, Guenther A, McLean AL, Waschke A, Kalff R, Ewald C. Prediction of 30-day mortality in spontaneous cerebellar hemorrhage. *Surg Neurol Int*. 2017;8:282. Published 2017 Nov 20. doi:10.4103/sni.sni_479_16
2. Asadollahi S, Vafaei A, Heidari K. CT imaging for long-term functional outcome after spontaneous intracerebral haemorrhage: A 3-year follow-up study. *Brain Inj*. 2016;30(13-14):1626-1634. doi:10.1080/02699052.2016.1199909
3. Appelboom G, Piazza MA, Hwang BY, et al. Severity of intraventricular extension correlates with level of admission glucose after intracerebral hemorrhage. *Stroke*. 2011;42(7):1883-1888. doi:10.1161/STROKEAHA.110.608166
4. Bakhshayesh B, Hosseininezhad M, Seyed Saadat SM, Hajmanuchehri M, Kazemnezhad E, Ghayeghran AR. Predicting in-hospital mortality in Iranian patients with spontaneous intracerebral hemorrhage. *Iran J Neurol*. 2014;13(4):231-236.
5. Bhatia R, Singh H, Singh S, et al. A prospective study of in-hospital mortality and discharge outcome in spontaneous intracerebral hemorrhage. *Neurol India*. 2013;61(3):244-248. doi:10.4103/0028-3886.115062
6. Bhattathiri PS, Gregson B, Prasad KS, Mendelow AD; STICH Investigators. Intraventricular hemorrhage and hydrocephalus after spontaneous intracerebral hemorrhage: results from the STICH trial. *Acta Neurochir Suppl*. 2006;96:65-68. doi:10.1007/3-211-30714-1_16
7. Buensuceso AM. Predictors of mortality based on CT Scan findings of patient admitted due to hypertensive intracerebral hemorrhage at the Philippine Heart Center. *Phil Heart Center J.* 2007; 13(2): 155-160
8. Celikbilek A, Goksel BK, Zararsiz G, Benli S. Spontaneous intra-cerebral hemorrhage: A retrospective study of risk factors and outcome in a Turkish population. *J Neurosci Rural Pract*. 2013;4(3):271-277. doi:10.4103/0976-3147.118770
9. Chan E, Anderson CS, Wang X, et al. Significance of intraventricular hemorrhage in acute intracerebral hemorrhage: intensive blood pressure reduction in acute cerebral hemorrhage trial results. *Stroke*. 2015;46(3):653-658. doi:10.1161/STROKEAHA.114.008470
10. Chaturbedi A, Thakur J. Functional outcome survival and independent risk factors in patients with spontaneous intracerebral hemorrhage from chronic arterial hypertension. *J Neurol Neurophys*. 2020; 11(7): 001-005
11. Chen G, Ping L, Zhou S, et al. Early prediction of death in acute hypertensive intracerebral hemorrhage. *Exp Ther Med*. 2016;11(1):83-88. doi:10.3892/etm.2015.2892
12. Cheung RT, Zou LY. Use of the original, modified, or new intracerebral hemorrhage score to predict mortality and morbidity after intracerebral hemorrhage. *Stroke*. 2003;34(7):1717-1722. doi:10.1161/01.STR.0000078657.22835.B9
13. Chuang YC, Chen YM, Peng SK, Peng SY. Risk stratification for predicting 30-day mortality of intracerebral hemorrhage. *Int J Qual Health Care*. 2009;21(6):441-447. doi:10.1093/intqhc/mzp041
14. Daverat P, Castel JP, Dartigues JF, Orgogozo JM. Death and functional outcome after spontaneous intracerebral hemorrhage. A prospective study of 166 cases using multivariate analysis. *Stroke*. 1991;22(1):1-6. doi:10.1161/01.str.22.1.1
15. Di Napoli M, Godoy DA, Campi V, et al. C-reactive protein level measurement improves mortality prediction when added to the spontaneous intracerebral hemorrhage score. *Stroke*. 2011;42(5):1230-1236. doi:10.1161/STROKEAHA.110.604983
16. Diringer MN, Edwards DF, Zazulia AR. Hydrocephalus: a previously unrecognized predictor of poor outcome from supratentorial intracerebral hemorrhage. *Stroke*. 1998;29(7):1352-1357. doi:10.1161/01.str.29.7.1352
17. El-Saadany WF, Hassan T. Adult intraventricular hemorrhage: presentations, management, and analysis of outcome. *Neurosurg Q.* 2012; 22(1): 30-7.
18. Eslami V, Tahsili-Fahadan P, Rivera-Lara L, et al. Influence of Intracerebral Hemorrhage Location on Outcomes in Patients With Severe Intraventricular Hemorrhage. *Stroke*. 2019;50(7):1688-1695. doi:10.1161/STROKEAHA.118.024187
19. Giray S, Sen O, Sarica FB, et al. Spontaneous primary intraventricular hemorrhage in adults: clinical data, etiology and outcome. *Turk Neurosurg*. 2009;19(4):338-344.
20. Godoy DA, Piñero G, Di Napoli M. Predicting mortality in spontaneous intracerebral hemorrhage: can modification to original score improve the prediction?. *Stroke*. 2006;37(4):1038-1044. doi:10.1161/01.STR.0000206441.79646.49
21. Grand W, Leonardo J, Chamczuk AJ, Korus AJ. Endoscopic Third Ventriculostomy in 250 Adults With Hydrocephalus: Patient Selection, Outcomes, and Complications. *Neurosurgery*. 2016;78(1):109-119. doi:10.1227/NEU.0000000000000994
22. Guo R, Ma L, Shrestha BK, Yu Z, Li H, You C. A retrospective clinical study of 98 adult idiopathic primary intraventricular hemorrhage cases. *Medicine (Baltimore)*. 2016;95(42):e5089. doi:10.1097/MD.0000000000005089
23. Hameed B, Khealani BA, Mozzafar T, Wasay M. Prognostic indicators in patients with primary intraventricular haemorrhage. *J Pak Med Assoc*. 2005;55(8):315-317.
24. Hegde A, Menon G, Kumar V, et al. Clinical Profile and Predictors of Outcome in Spontaneous Intracerebral Hemorrhage from a Tertiary Care Centre in South India. *Stroke Res Treat*. 2020;2020:2192709. Published 2020 Jan 27. doi:10.1155/2020/2192709
25. Hemphill JC 3rd, Bonovich DC, Besmertis L, Manley GT, Johnston SC. The ICH score: a simple, reliable grading scale for intracerebral hemorrhage. *Stroke*. 2001;32(4):891-897. doi:10.1161/01.str.32.4.891
26. Herrick DB, Ullman N, Nekoovaght-Tak S, et al. Determinants of external ventricular drain placement and associated outcomes in patients with spontaneous intraventricular hemorrhage. *Neurocrit Care*. 2014;21(3):426-434. doi:10.1007/s12028-014-9959-x
27. Hughes JD, Puffer R, Rabinstein AA. Risk factors for hydrocephalus requiring external ventricular drainage in patients with intraventricular hemorrhage. *J Neurosurg*. 2015;123(6):1439-1446. doi:10.3171/2015.1.JNS142391
28. Huttner HB, Köhrmann M, Berger C, Georgiadis D, Schwab S. Influence of intraventricular hemorrhage and occlusive hydrocephalus on the long-term outcome of treated patients with basal ganglia hemorrhage: a case-control study. *J Neurosurg*. 2006;105(3):412-417. doi:10.3171/jns.2006.105.3.412
29. Hwang BY, Bruce SS, Appelboom G, et al. Evaluation of intraventricular hemorrhage assessment methods for predicting outcome following intracerebral hemorrhage. *J Neurosurg*. 2012;116(1):185-192. doi:10.3171/2011.9.JNS10850
30. Inagawa T, Shibukawa M, Inokuchi F, Tokuda Y, Okada Y, Okada K. Primary intracerebral and aneurysmal subarachnoid hemorrhage in Izumo City, Japan. Part II: management and surgical outcome. *J Neurosurg*. 2000;93(6):967-975. doi:10.3171/jns.2000.93.6.0967
31. Ironside N, Chen CJ, Dreyer V, Christophe B, Buell TJ, Connolly ES. Location-specific differences in hematoma volume predict outcomes in patients with spontaneous intracerebral hemorrhage. *Int J Stroke*. 2020;15(1):90-102. doi:10.1177/1747493019830589
32. Kim KH. Predictors of 30-day mortality and 90-day functional recovery after primary intracerebral hemorrhage : hospital based multivariate analysis in 585 patients. *J Korean Neurosurg Soc*. 2009;45(6):341-349. doi:10.3340/jkns.2009.45.6.341
33. Kim KR, Kim YZ. Clinical Comparison of 30-Day Mortalities and 6-Month Functional Recoveries after Spontaneous Intracerebral Hemorrhage in Patients with or without End-Stage Renal Disease. *J Korean Neurosurg Soc*. 2013;54(3):164-174. doi:10.3340/jkns.2013.54.3.164
34. Kim BJ, Lee SH, Ryu WS, et al. Extents of white matter lesions and increased intraventricular extension of intracerebral hemorrhage. *Crit Care Med*. 2013;41(5):1325-1331. doi:10.1097/CCM.0b013e31827c05e9
35. Koivunen RJ, Satopää J, Haapaniemi E, et al. Predictors of early mortality in young adults after intracerebral hemorrhage. *Stroke*. 2014;45(8):2454-2456. doi:10.1161/STROKEAHA.114.006020
36. Koivunen RJ, Tatlisumak T, Satopää J, Niemelä M, Putaala J. Intracerebral hemorrhage at young age: long-term prognosis. *Eur J Neurol*. 2015;22(7):1029-1037. doi:10.1111/ene.12704
37. Lai SL, Chen ST, Lee TH, Ro LS, Hsu SP. Spontaneous intracerebral hemorrhage in young adults. *Eur J Neurol*. 2005;12(4):310-316. doi:10.1111/j.1468-1331.2004.00957.x
38. Lee SH, Park KJ, Park DH, Kang SH, Park JY, Chung YG. Factors Associated with Clinical Outcomes in Patients with Primary Intraventricular Hemorrhage. *Med Sci Monit*. 2017;23:1401-1412. Published 2017 Mar 22. doi:10.12659/msm.899309
39. Liliang PC, Liang CL, Lu CH, et al. Hypertensive caudate hemorrhage prognostic predictor, outcome, and role of external ventricular drainage. *Stroke*. 2001;32(5):1195-1200. doi:10.1161/01.str.32.5.1195
40. Lim MJR, Neo AYY, Singh GD, et al. The Evaluation of Prognostic Scores in Spontaneous Intracerebral Hemorrhage in an Asian Population: A Retrospective Study. *J Stroke Cerebrovasc Dis*. 2020;29(12):105360. doi:10.1016/j.jstrokecerebrovasdis.2020.105360
41. Liotta, E. M., Singh, M., Kosteva, A. R., Beaumont, J. L., Guth, J. C., Bauer, R. M., Prabhakaran, S., Rosenberg, N. F., Maas, M. B., & Naidech, A. M. (2013). Predictors of 30-day readmission after intracerebral hemorrhage: a single-center approach for identifying potentially modifiable associations with readmission. *Critical care medicine*, *41*(12), 2762–2769. <https://doi.org/10.1097/CCM.0b013e318298a10f>
42. Louis EK, Wijdicks EF, Li H, Atkinson JD. Predictors of poor outcome in patients with a spontaneous cerebellar hematoma. *Can J Neurol Sci*. 2000;27(1):32-36. doi:10.1017/s0317167100051945
43. Mahta A, Katz PM, Kamel H, Azizi SA. Intracerebral hemorrhage with intraventricular extension and no hydrocephalus may not increase mortality or severe disability. *J Clin Neurosci*. 2016;30:56-59. doi:10.1016/j.jocn.2015.11.028
44. Mansouri B, Heidari K, Asadollahi S, Nazari M, Assarzadegan F, Amini A. Mortality and functional disability after spontaneous intracranial hemorrhage: the predictive impact of overall admission factors. *Neurol Sci*. 2013;34(11):1933-1939. doi:10.1007/s10072-013-1410-0
45. Martí-Fàbregas J, Piles S, Guardia E, Martí-Vilalta JL. Spontaneous primary intraventricular hemorrhage: clinical data, etiology and outcome. *J Neurol*. 1999;246(4):287-291. doi:10.1007/s004150050348
46. Masè G, Zorzon M, Biasutti E, Tasca G, Vitrani B, Cazzato G. Immediate prognosis of primary intracerebral hemorrhage using an easy model for the prediction of survival. *Acta Neurol Scand*. 1995;91(4):306-309. doi:10.1111/j.1600-0404.1995.tb07011.x
47. Maslehaty H, Petridis AK, Barth H, Doukas A, Mehdorn HM. Treatment of 817 patients with spontaneous supratentorial intracerebral hemorrhage: characteristics, predictive factors and outcome. *Clin Pract*. 2012;2(3):e56. Published 2012 May 17. doi:10.4081/cp.2012.e56
48. Mayfrank L, Hütter BO, Kohorst Y, et al. Influence of intraventricular hemorrhage on outcome after rupture of intracranial aneurysm. *Neurosurg Rev*. 2001;24(4):185-191. doi:10.1007/s101430100160
49. Mustanoja S, Satopää J, Meretoja A, et al. Extent of secondary intraventricular hemorrhage is an independent predictor of outcomes in intracerebral hemorrhage: data from the Helsinki ICH Study. *Int J Stroke*. 2015;10(4):576-581. doi:10.1111/ijs.12437
50. Nishikawa T, Ueba T, Kajiwara M, Miyamatsu N, Yamashita K. A priority treatment of the intraventricular hemorrhage (IVH) should be performed in the patients suffering intracerebral hemorrhage with large IVH. *Clin Neurol Neurosurg*. 2009;111(5):450-453. doi:10.1016/j.clineuro.2009.01.005
51. OH HM, Rhee DY, Park HS, Song JS, Heo W, Lee CJ, Joung SH. Clinical analysis of prognostic factors in primary intraventricular hemorrhage. *Kor J Cerebrovas Surg*. 2008; 10(3): 419-23.
52. Pai A, Hegde A, Nair R, Menon G. Adult Primary Intraventricular Hemorrhage: Clinical Characteristics and Outcomes. *J Neurosci Rural Pract*. 2020;11(4):623-628. doi:10.1055/s-0040-1716770
53. Parry-Jones AR, Abid KA, Di Napoli M, et al. Accuracy and clinical usefulness of intracerebral hemorrhage grading scores: a direct comparison in a UK population. *Stroke*. 2013;44(7):1840-1845. doi:10.1161/STROKEAHA.113.001009
54. Phan TG, Koh M, Vierkant RA, Wijdicks EF. Hydrocephalus is a determinant of early mortality in putaminal hemorrhage. *Stroke*. 2000;31(9):2157-2162. doi:10.1161/01.str.31.9.2157
55. Pong V, Chan KH, Chong BH, et al. Long-term outcome and prognostic factors after spontaneous cerebellar hemorrhage. *Cerebellum*. 2012;11(4):939-945. doi:10.1007/s12311-012-0371-9
56. Portenoy RK, Lipton RB, Berger AR, Lesser ML, Lantos G. Intracerebral haemorrhage: a model for the prediction of outcome. *J Neurol Neurosurg Psychiatry*. 1987; 50:976-9.
57. Qureshi AI, Safdar K, Weil J, et al. Predictors of early deterioration and mortality in black Americans with spontaneous intracerebral hemorrhage. *Stroke*. 1995;26(10):1764-1767. doi:10.1161/01.str.26.10.1764
58. Razzaq AA, Hussain R. Determinants of 30-day mortality of spontaneous intracerebral hemorrhage in Pakistan. *Surg Neurol*. 1998;50(4):336-343. doi:10.1016/s0090-3019(98)00089-5
59. Reddy GK. Ventriculoperitoneal shunt surgery and the incidence of shunt revision in adult patients with hemorrhage-related hydrocephalus. *Clin Neurol Neurosurg*. 2012;114(9):1211-1216. doi:10.1016/j.clineuro.2012.02.050
60. Roeder SS, Sprügel MI, Sembill JA, et al. Influence of the Extent of Intraventricular Hemorrhage on Functional Outcome and Mortality in Intracerebral Hemorrhage. *Cerebrovasc Dis*. 2019;47(5-6):245-252. doi:10.1159/000501027
61. Shimoda Y, Ohtomo S, Arai H, Okada K, Tominaga T. Satellite Sign: A Poor Outcome Predictor in Intracerebral Hemorrhage. *Cerebrovasc Dis*. 2017;44(3-4):105-112. doi:10.1159/000477179
62. Sloan MA, Sila CA, Mahaffey KW, et al. Prediction of 30-day mortality among patients with thrombolysis-related intracranial hemorrhage. *Circulation*. 1998;98(14):1376-1382. doi:10.1161/01.cir.98.14.1376
63. Staykov D, Volbers B, Wagner I, et al. Prognostic significance of third ventricle blood volume in intracerebral haemorrhage with severe ventricular involvement. *J Neurol Neurosurg Psychiatry*. 2011;82(11):1260-1263. doi:10.1136/jnnp.2010.234542
64. Stein M, Luecke M, Preuss M, Boeker DK, Joedicke A, Oertel MF. Spontaneous intracerebral hemorrhage with ventricular extension and the grading of obstructive hydrocephalus: the prediction of outcome of a special life-threatening entity. *Neurosurgery*. 2010;67(5):1243-1252. doi:10.1227/NEU.0b013e3181ef25de
65. Stein M, Hamann GF, Misselwitz B, Uhl E, Kolodziej M, Reinges MHT. In-Hospital Mortality and Complication Rates in Surgically and Conservatively Treated Patients with Spontaneous Intracerebral Hemorrhage in Central Europe: A Population-Based Study. *World Neurosurg*. 2016;88:306-310. doi:10.1016/j.wneu.2015.11.075
66. Takahashi O, Cook EF, Nakamura T, Saito J, Ikawa F, Fukui T. Risk stratification for in-hospital mortality in spontaneous intracerebral haemorrhage: a Classification and Regression Tree analysis. *QJM*. 2006;99(11):743-750. doi:10.1093/qjmed/hcl107
67. Togha M, Bakhtavar K. Factors associated with in-hospital mortality following intracerebral hemorrhage: a three-year study in Tehran, Iran. *BMC Neurol*. 2004;4:9. Published 2004 Jun 14. doi:10.1186/1471-2377-4-9
68. Trifan G, Arshi B, Testai FD. Intraventricular Hemorrhage Severity as a Predictor of Outcome in Intracerebral Hemorrhage. *Front Neurol*. 2019;10:217. Published 2019 Mar 12. doi:10.3389/fneur.2019.00217
69. Tshikwela ML, Longo-Mbenza B. Spontaneous intracerebral hemorrhage: Clinical and computed tomography findings in predicting in-hospital mortality in Central Africans. *J Neurosci Rural Pract*. 2012;3(2):115-120. doi:10.4103/0976-3147.98205
70. Wang GQ, Li SQ, Huang YH, et al. Can minimally invasive puncture and drainage for hypertensive spontaneous Basal Ganglia intracerebral hemorrhage improve patient outcome: a prospective non-randomized comparative study. *Mil Med Res*. 2014;1:10. Published 2014 Jun 1. doi:10.1186/2054-9369-1-10
71. Woo KM, Yang SY, Cho KT. Seizures after spontaneous intracerebral hemorrhage. *J Korean Neurosurg Soc*. 2012;52(4):312-319. doi:10.3340/jkns.2012.52.4.312
72. Wu YT, Li TY, Chiang SL, Chu HY, Chang ST, Chen LC. Predictors of first-week mortality in patients with acute spontaneous cerebellar hemorrhage. *Cerebellum*. 2013;12(2):165-170. doi:10.1007/s12311-012-0410-6
73. Yaghi S, Dibu J, Achi E, Patel A, Samant R, Hinduja A. Hematoma expansion in spontaneous intracerebral hemorrhage: predictors and outcome. *Int J Neurosci* 2014; 124(12): 890-3. Doi: 10.3109/00207454.2014.887716
74. Yang TM, Lin WC, Chang WN, et al. Predictors and outcome of seizures after spontaneous intracerebral hemorrhage. Clinical article. *J Neurosurg*. 2009;111(1):87-93. doi:10.3171/2009.2.JNS081622
75. Ye Z, Ai X, Hu X, Fang F, You C. Clinical features and prognostic factors in patients with intraventricular hemorrhage caused by ruptured arteriovenous malformations. *Medicine (Baltimore)*. 2017;96(45):e8544. doi:10.1097/MD.0000000000008544
76. Yuan R, Lei C, Wu S, et al. Prognostic Significance of Intraventricular Hemorrhage in Vascular Structural Abnormality-Related Intracerebral Hemorrhage. *J Stroke Cerebrovasc Dis*. 2017;26(3):636-643. doi:10.1016/j.jstrokecerebrovasdis.2016.11.012
77. Zahuranec DB, Brown DL, Lisabeth LD, et al. Early care limitations independently predict mortality after intracerebral hemorrhage. *Neurology*. 2007;68(20):1651-1657. doi:10.1212/01.wnl.0000261906.93238.72
78. Zaidi HA, Montoure A, Elhadi A, et al. Long-term functional outcomes and predictors of shunt-dependent hydrocephalus after treatment of ruptured intracranial aneurysms in the BRAT trial: revisiting the clip vs coil debate. *Neurosurgery*. 2015;76(5):608-614. doi:10.1227/NEU.0000000000000677
79. Zhang S, Jia B, Li H, You C, Hanley DF, Jiang Y. Primary intraventricular hemorrhage in adults: etiological causes and prognostic factors in Chinese population. *J Neurol*. 2017;264(2):382-390. doi:10.1007/s00415-016-8367-x
80. Bender M, Haferkorn K, Friedrich M, Uhl E, Stein M. Impact of Early C-Reactive Protein/Albumin Ratio on Intra-Hospital Mortality Among Patients with Spontaneous Intracerebral Hemorrhage. *J Clin Med*. 2020;9(4):1236. Published 2020 Apr 24. doi:10.3390/jcm9041236
81. Doukas A, Maslehaty H, Barth H, Hedderich J, Petridis AK, Mehdorn HM. A novel simple measure correlates to the outcome in 57 patients with intracerebellar hematomas. Results of a retrospective analysis. *Surg Neurol Int*. 2015;6:176. Published 2015 Nov 23. doi:10.4103/2152-7806.170246
82. Kongwad LI, Hegde A, Menon G, Nair R. Influence of Admission Blood Glucose in Predicting Outcome in Patients With Spontaneous Intracerebral Hematoma. *Front Neurol*. 2018;9:725. Published 2018 Aug 28. doi:10.3389/fneur.2018.00725
83. Lee TH, Huang YH, Su TM, et al. Predictive Factors of 2-Year Postoperative Outcomes in Patients with Spontaneous Cerebellar Hemorrhage. *J Clin Med*. 2019;8(6):818. Published 2019 Jun 8. doi:10.3390/jcm8060818
84. Moullaali TJ, Sato S, Wang X, et al. Prognostic significance of delayed intraventricular haemorrhage in the INTERACT studies. *J Neurol Neurosurg Psychiatry*. 2017;88(1):19-24. doi:10.1136/jnnp-2015-311562
85. Øie LR, Madsbu MA, Solheim O, et al. Functional outcome and survival following spontaneous intracerebral hemorrhage: A retrospective population-based study. *Brain Behav*. 2018;8(10):e01113. doi:10.1002/brb3.1113
86. Peng SY, Chuang YC, Kang TW, Tseng KH. Random forest can predict 30-day mortality of spontaneous intracerebral hemorrhage with remarkable discrimination. *Eur J Neurol*. 2010;17(7):945-950. doi:10.1111/j.1468-1331.2010.02955.x
87. Safatli DA, Günther A, Schlattmann P, Schwarz F, Kalff R, Ewald C. Predictors of 30-day mortality in patients with spontaneous primary intracerebral hemorrhage. *Surg Neurol Int*. 2016;7(Suppl 18):S510-S517. Published 2016 Aug 1. doi:10.4103/2152-7806.187493
88. Satopää J, Meretoja A, Koivunen RJ, et al. Treatment of intracerebellar haemorrhage: Poor outcome and high long-term mortality. *Surg Neurol Int*. 2017;8:272. Published 2017 Nov 9. doi:10.4103/sni.sni_168_17
89. Szepesi R, Széll IK, Hortobágyi T, et al. New prognostic score for the prediction of 30-day outcome in spontaneous supratentorial cerebral haemorrhage. *Biomed Res Int*. 2015;2015:961085. doi:10.1155/2015/961085
90. Tao C, Hu X, Wang J, You C. Effect of Admission Hyperglycemia on 6-Month Functional Outcome in Patients with Spontaneous Cerebellar Hemorrhage. *Med Sci Monit*. 2017;23:1200-1207. Published 2017 Mar 8. doi:10.12659/msm.900202
91. Weimar C, Roth M, Willig V, Kostopoulos P, Benemann J, Diener HC. Development and validation of a prognostic model to predict recovery following intracerebral hemorrhage. *J Neurol*. 2006;253(6):788-793. doi:10.1007/s00415-006-0119-x
92. Yang WS, Shen YQ, Zhang XD, et al. Hydrocephalus Growth: Definition, Prevalence, Association with Poor Outcome in Acute Intracerebral Hemorrhage. *Neurocrit Care*. 2021;35(1):62-71. doi:10.1007/s12028-020-01140-w
93. Zis P, Leivadeas P, Michas D, Kravaritis D, Angelidakis P, Tavernarakis A. Predicting 30-day case fatality of primary inoperable intracerebral hemorrhage based on findings at the emergency department. *J Stroke Cerebrovasc Dis*. 2014;23(7):1928-1933. doi:10.1016/j.jstrokecerebrovasdis.2014.02.006
94. Di Napoli M, Godoy DA, Campi V, et al. C-reactive protein in intracerebral hemorrhage: time course, tissue localization, and prognosis. *Neurology*. 2012;79(7):690-699. doi:10.1212/WNL.0b013e318264e3be
95. Biller J, Toffol GJ, Kassell NF, Adams HP Jr, Beck DW, Boarini DJ. Spontaneous subarachnoid hemorrhage in young adults. *Neurosurgery*. 1987;21(5):664-667. doi:10.1227/00006123-198711000-00011
96. van Gijn J, Hidjra A, Wijdicks EFM, Vermeulen M, van Crevel H. Acute hydrocephalus after aneurysmal subarachnoid hemorrhage. *J Neurosurg* 1985; 63:355-62.
97. Hyun MH, Kim KM, Bak KH, Shink HS, Kim JM, Kim YS. Clinical analysis of subarachnoid hemorrhage with intraventricular hemorrhage due to aneurysmal rupture. *J Kor Neurosurg Soc*. 1997; 26:191-195.
98. Kang DH, Park J, Park SH, Kim YS, Hwang SK, Hamm IS. Early ventriculoperitoneal shunt placement after severe aneurysmal subarachnoid hemorrhage: role of intraventricular hemorrhage and shunt function. *Neurosurgery*. 2010;66(5):904-909. doi:10.1227/01.NEU.0000368385.74625.96
99. Lagares A, Gómez PA, Lobato RD, Alén JF, Alday R, Campollo J. Prognostic factors on hospital admission after spontaneous subarachnoid haemorrhage. *Acta Neurochir (Wien)*. 2001;143(7):665-672. doi:10.1007/s007010170044
100. Navi BB, Reichman JS, Berlin D, et al. Intracerebral and subarachnoid hemorrhage in patients with cancer. *Neurology*. 2010;74(6):494-501. doi:10.1212/WNL.0b013e3181cef837
101. Paisan GM, Ding D, Starke RM, Crowley RW, Liu KC. Shunt-Dependent Hydrocephalus After Aneurysmal Subarachnoid Hemorrhage: Predictors and Long-Term Functional Outcomes. *Neurosurgery*. 2018;83(3):393-402. doi:10.1093/neuros/nyx393
102. Pasqualin A, Mazza C, Cavazzani P, Scienza R, DaPian R. Intracranial aneurysms and subarachnoid hemorrhage in children and adolescents. *Childs Nerv Syst*. 1986;2(4):185-190. doi:10.1007/BF00706808
103. Sheikhazadi A, Gharehdaghi J. Survey of sudden death from aneurysmal subarachnoid hemorrhage in cadavers referred to Legal Medicine Organization of Tehran, 2001-2005. *Am J Forensic Med Pathol*. 2009;30(4):358-361. doi:10.1097/PAF.0b013e3181bfcd64
104. Wang YM, Lin YJ, Chuang MJ, et al. Predictors and outcomes of shunt-dependent hydrocephalus in patients with aneurysmal sub-arachnoid hemorrhage. *BMC Surg*. 2012;12:12. Published 2012 Jul 5. doi:10.1186/1471-2482-12-12
105. Zhao B, Zhao Y, Tan X, et al. Factors and outcomes associated with ultra-early surgery for poor-grade aneurysmal subarachnoid haemorrhage: a multicentre retrospective analysis. *BMJ Open*. 2015;5(4):e007410. Published 2015 Apr 15. doi:10.1136/bmjopen-2014-007410
